# Gap Analysis of Metabolic Conversions of Off‐Flavors and Antinutrients in Plant‐Based Substrates

**DOI:** 10.1111/1541-4337.70449

**Published:** 2026-03-31

**Authors:** Robin I. Kuijpers, Isabel O. de Moya Clark, Tomás Cavaco, Vivian Nemanič, Beatrice Tagliabue, Ainhoa Abad Valero, Wiebe M. Wennekers, Mengqiu Zhang, Koen Van Zwet, Sanne Abeln, Sofia Moco, Caroline E. Paul, Halima Mouhib, Richard A. Notebaart, Eddy J. Smid, Bas Teusink, Herwig Bachmann

**Affiliations:** ^1^ Systems Biology Lab, A‐LIFE, AIMMS Vrije Universiteit Amsterdam Amsterdam the Netherlands; ^2^ Department of Computer Science, VU Bioinformatics Group Vrije Universiteit Amsterdam Amsterdam the Netherlands; ^3^ Food Microbiology Wageningen University & Research Wageningen the Netherlands; ^4^ Biocatalysis Section, Department of Biotechnology Delft University of Technology Delft the Netherlands; ^5^ Chemistry and Pharmaceutical Sciences, AIMMS Vrije Universiteit Amsterdam Amsterdam the Netherlands; ^6^ Department of Computer Science, AI Technology for Life Universiteit Utrecht Utrecht the Netherlands; ^7^ NIZO Food Research Ede the Netherlands

**Keywords:** antinutritional factors, biocatalysis, enzymes, fermentation, flavor, lactic acid bacteria, metabolism, plant‐based food, yeast

## Abstract

To drastically reduce the carbon footprint of the food production chain, a major shift towards alternatives to conventional meat and dairy products is required. The use of plant‐based proteins is a promising route, but it also comes with challenges: Plant‐based proteins often contain antinutritional factors and off‐flavors, which can negatively impact consumer acceptance. Fermentation is broadly used to improve the quality of these products. However, how these unwanted molecules are synthesized and degraded is poorly understood, but this knowledge is essential for fermentation‐based strategies to improve the sensory and nutritional value of plant‐based products. This review provides a comprehensive overview of synthesis and degradation pathways of key antinutritional factors and off‐flavor compounds in plant‐based substrates, including aldehydes, furans, sulfur compounds, pyrazines, glycoalkaloids (GAs), pyrimidine glycosides, polyphenols, saponins, glucosinolates (GSLs), phytic acid (PA), oxalates, lectins, and protease and amylase inhibitors. With this we identified the research gaps in the field, which can be divided into three types: (i) degradation pathways that are unknown (furans, alkyl‐methoxypyrazines, and dimethyl trisulfide), (ii) well‐characterized pathways but typically not found in food‐grade organisms (dimethyl sulfide, dimethyl disulfide, and isothiocyanates derived from GSLs), and (iii) pathways that are only described partially (GAs, saponins, polyphenols, PA, and pyrimidine glycosides). Other molecule classes, like aldehydes, alcohols, and oxalate, have well‐characterized degradation pathways in food‐grade organisms. Focusing future research on compounds with poorly understood degradation pathways will help to accelerate the development of more rationally designed cultures for producing healthy and sustainable plant‐based foods.

## 1 Introduction

Climate change is one of the most pressing challenges of our time, and the food supply chain is responsible for 26% of global greenhouse gas emissions (Poore and Nemecek 2018). Plant‐based products in general have a lower carbon footprint than animal‐based products; for example, greenhouse gas emissions of protein from beef are 48 times higher compared to pea protein (Poore and Nemecek 2018). Transitioning towards a more plant‐based diet is therefore considered essential for making the food supply system more sustainable. However, there remains a high demand for dairy and meat products, creating a market for plant‐based alternatives (OECD/FAO 2025; Tangyu et al. 2023). These are often produced using purified plant proteins, but many of them come with challenges, as they contain off‐flavors and antinutrients (Roland et al. 2017; Samtiya et al. 2020).

Volatiles, such as aldehydes, alcohols, ketones, furans, sulfur compounds, and pyrazines, can contribute to unpleasant aromas like grassy, beany, or sulfurous notes. Nonvolatiles, such as polyphenols, glycoalkaloids (GAs), saponins, and glucosinolates (GSLs), can negatively impact taste, contributing to bitterness or astringency (Leonard et al. 2023; Roland et al. 2017). In addition, nonvolatiles can function as antinutritional factors by reducing mineral bioavailability or inhibiting digestive enzymes. These include GAs, saponins, GSLs, phytic acid (PA), oxalate, lectins, protease, and amylase inhibitors (AIs) (Popova and Mihaylova 2019; Samtiya et al. 2020).

During fermentation, off‐flavors and antinutrients can be degraded or converted, which improves the sensory and nutritional profiles of plant‐based proteins (Tangyu et al. 2023). Microorganisms with a qualified presumption of safety (QPS) status are considered safe to use in food applications. Many of these microbes, particularly lactic acid bacteria (LAB) and yeasts, have a long history of use in traditional food fermentations and show potential in improving plant‐based substrates. However, due to limited understanding of the underlying biochemical reactions, current fermentation strategies for novel plant‐based products rely on trial‐and‐error, through, for example, varying microorganisms, processing conditions, and plant‐based substrates (El Youssef et al. 2020; Engels et al. 2022; Nugroho et al. 2024). To develop targeted fermentation strategies, a better understanding of these metabolic pathways is needed.

Although other reviews focused on the challenges of off‐flavors and antinutrients (Leonard et al. 2023), processing strategies to control off‐flavor and protein interactions (Saffarionpour 2024), or metabolic conversions of off‐flavors and antinutrients by LABs (Molina et al. 2025), this review aims to reconstruct the known enzymatic pathways involved in the biosynthesis and degradation of off‐flavors and antinutrients. Instead of focusing on degradation pathways within a single organism, we collected all the reactions described in literature to illustrate the types of enzymes required to degrade these compounds. Although the degradation pathways are obviously relevant to fermentation design, the inclusion of biosynthetic pathways is potentially relevant for the identification of enzymes. To cover biodiversity in a broader sense, the review is not limited to QPS‐listed microorganisms. However, to explore the potential of food‐grade organisms, such as LABs and yeasts, we carried out sequence‐based searches for the presence of these enzymes in selected species. From this, we identified key knowledge gaps in the field.

## 2 Methods

### 2.1 Biochemical Synthesis and Degradation Pathways

UniProt and BRENDA databases were searched to find enzymes responsible for the synthesis and degradation of the compounds of interest. Enzymes were included only if their catalytic activity towards the substrates of interest was experimentally verified. Only these enzymes are shown in the figures with their corresponding EC number. Reactions with known intermediates but unverified enzymes are shown using two parallel dashed arrows, indicating that both the intermediate steps and the catalysts (chemical or enzymatic) are unknown. Only chemical reactions that occur spontaneously or during processing conditions of our plant‐based substrates, such as heating (∆), were included. The EC number, gene name, protein name, UniProt ID, RHEA, genome accession, and protein accession are given in Table S1. The final schemes were created with ChemDraw. Compound names were collected from PubChem.

### 2.2 Bioinformatic Analysis

Sequences of degradation enzymes were retrieved from UniProt (Bateman et al. 2025). Enzymes were included only if they were annotated on UniProt with evidence at the protein or transcript level or if there was a paper available experimentally validating the function of the enzyme. Only proteins from either Bacteria (taxid:2) or Ascomycota (taxid: 4890) or from both whenever possible were included. For each enzyme, the proteome of its source organism and the NCBI protein accession number were retrieved. A species list was made covering relevant LAB (Qiao et al. 2022; Rossi 2023), yeast, and filamentous fungi relevant for food fermentation (Table S2). Species from the QPS list (Allende et al. 2025) were also included. The proteome of the listed species was retrieved from NCBI (O'Leary et al. 2024) (v18.1.0). The proteomes of all species and a species phylogenetic tree generated using PhyloT (Ivica Letunić n.d.; Schoch et al. 2020) were provided to OrthoFinder (Emms and Kelly 2015) (v3.0.1b1) as input. The following parameters were used: ‐S diamond ‐M msa ‐A mafft ‐T fasttree. The N0.tsv orthologous group output file was filtered to retain only the orthogroups containing the NCBI protein accession number of enzymes in our list. The final figure was generated in R using ggplot2 (Hadley Wickham 2016) (v3.5.2) and ggtree (Yu et al. 2017) (v3.17.1.1). A more detailed description of the method can be found in Section S2 and Figure S1.

## 3 Aldehydes, Ketones, and Alcohols

### 3.1 Biosynthesis of Aldehydes, Alcohols, and Ketones

Aldehydes, alcohols, and ketones are the main volatile compounds responsible for “green” and “beany” off‐flavors in plant‐based food (Leonard et al. 2023; Vatansever et al. 2024). These molecules are synthetized in plants in response to different stresses, but they also play important roles as signaling molecules during growth and development (Dudareva et al. 2013; Liang et al. 2022).

Aldehydes such as hexanal and nonanal contribute “green,” “grassy,” and “fatty” notes at odor thresholds that can be as low as 0.05 ppb. (*E*,*E*)‐2,4‐decadienal is linked to “fatty” and “oily” odors at concentrations below 0.1 ppb. Alcohols such as 1‐hexanol and 1‐octen‐3‐ol provide “floral,” “grassy,” or “mushroom‐like” notes, typically at higher detection thresholds ranging from 0.19 to 250 ppb. All odor thresholds of these compounds were measured in water. Ketones, including 2‐heptanone and 2‐nonanone, contribute “fruity,” “sweet,” or “cheesy” aromas and are generally detectable at higher ppb ranges compared to those found for aldehydes (Akkad et al. 2021; Molina et al. 2025; Tangyu et al. 2023; Z. Wang, Gao, et al. 2022; Zhang et al. 2020).

The main pathway leading to the formation of many aldehydes and the corresponding alcohols is the lipid oxidation pathway (lipoxygenase [LOX] pathway) (Sarang et al. 2021; Vincenti et al. 2019). The biosynthetic process typically begins with the hydrolysis of triglycerides by lipases such as triacylglycerol acylhydrolase/lipase (EC 3.1.1.3), releasing free polyunsaturated fatty acids (PUFAs), mainly linoleic and linolenic acid (Karolkowski et al. 2021). Linoleic and linolenic acids can then be oxidized by LOXs. In plants, peroxidation typically occurs at the C9 position catalyzed by 9‐LOX (EC 1.13.11.58) or C13 position catalyzed by 13‐LOX (EC 1.13.11.12) of the fatty acid, leading to the conversion of linoleic acid into (9*S*)‐hydroperoxy octadecadienoic acid or (13*S*)‐hydroperoxy octadecadienoic acid. Hydroperoxide lyases (EC 4.2.99.‐) cleave these hydroperoxides, producing different products depending on the substrate. From 9‐hydroperoxides, the reaction yields C9 aldehydes, such as (*Z*)‐non‐3‐enal or (3*Z*,6*Z*)‐nona‐3,6‐dienal, along with 9‐oxo‐nonanoic acid. From 13‐hydroperoxides, C6 aldehydes, such as hexanal and (*Z*)‐hex‐3‐enal, are produced together with 12‐oxo‐nonanoic acid. Aldehydes can be further reduced to the corresponding alcohols by NAD(P)H‐dependent alcohol dehydrogenase (ADH, EC 1.1.1.1) (Bate et al. 1998; Duan et al. 2005; Schiller et al. 2015; Vincenti et al. 2019; Viswanath et al. 2020), or oxidized to their corresponding acids by NAD(P)‐dependent aldehyde dehydrogenases (ALDHs, EC 1.2.1.4) (Molina et al. 2025). The expression of ADHs in plants is highly regulated and tissue specific, and the conversion of C6 and C9 aldehydes occurs in specific stages of development, such as fruit ripening (Garabagi et al. 2005; Jin et al. 2016). Biosynthesis of ketones remains less understood, but they are likely synthesized from intermediates of the fatty acid biosynthetic pathway in plants or from the β‐oxidation pathway in fungi. In plants, a 3‐ketoacyl‐ACP thioesterase (EC 3.1.2.14) catalyzes the formation of 3‐ketoacids from a key intermediate in the fatty acid synthesis, 3‐ketoacyl‐acyl carrier protein. 3‐Ketoacids can then be decarboxylated to methyl ketones by a 3‐ketoacid decarboxylase (EC 4.1.1.‐). Fungi and bacteria can also produce ketones, such as 2‐heptanone and 2‐undecanone, but the specific enzymatic steps have not been elucidated (Forney and Markovetz 1971; Xu et al. 2025).

### 3.2 Formation of Aldehydes, Alcohols, and Ketones in Plant‐Based Products

The oxidation of fatty acids is one of the main causes for the formation of hexanal (Fischer et al. 2022). This oxidation process happens not only through the described LOX pathway but can also occur non‐enzymatically via radical oxidation in the presence of oxygen or through photooxidation. The production of these volatiles also increases during storage of the untreated plant materials due to light exposure, tissue disruption, high temperatures, and frost damage (Grebenteuch et al. 2021; Karolkowski et al. 2021; W. Wang, Du, et al. 2022).

### 3.3 Degradation of Aldehydes, Alcohols, and Ketones

Aldehydes are highly reactive and can be harmful to cells (Singh et al. 2013). For this reason, ALDHs (EC 1.2.1.4, EC 1.2.1.3) act as a primary detoxification system during stress responses, catalyzing their oxidation to the corresponding carboxylic acids (Islam and Ghosh 2022; Singh et al. 2013). ADHs (EC 1.1.1.1) also contribute to the degradation of aldehydes and ketones by catalyzing the reversible reduction to the corresponding alcohols (Nugroho et al. 2024).

### 3.4 Degradation of Aldehydes, Alcohols, and Ketones in Plant‐Based Products

Different processing techniques have been successful for the degradation of these compounds in plant‐based products. Soaking and cooking have been shown to decrease the concentration of volatile compounds in plant material. Dehulling of the whole seed is linked to a decrease in the alcohol content (Azarnia et al. 2011). Germination of faba beans led to a decrease in the aldehyde content, while increasing the alcohol and ketone content led to a better overall flavor profile (Akkad et al. 2021). Controlled enzymatic hydrolysis of protein isolates with a protease has also been linked to a decrease in the aldehydes content (Li et al. 2025). Fermentation with microbes that have a high expression levels of ADHs, like yeast and heterofermentative LABs, effectively improve flavor by reducing aldehydes into their corresponding alcohols (El Youssef et al. 2020; Fischer et al. 2022; Tangyu et al. 2023; Z. Wang, Gao, et al. 2022). Defining optimal fermentation processes and predicting their outcome remains challenging due to promiscuity of the described enzymes. As an example, ADHs from *Saccharomyces cerevisiae* show a broad substrate scope, spanning from acetaldehyde to longer chain aldehydes (pentanal, hexanal, and heptanal). In addition, some strains may even increase certain off‐flavors, such as hexanal, highlighting the importance of careful strain selection for fermentation treatments (Nugroho et al. 2024). To conclude, the biosynthesis and degradation of the off‐flavors aldehydes and alcohols are well understood.

## 4 Furans

### 4.1 Formation of Furans in Plant‐Based Products

**FIGURE 1:**
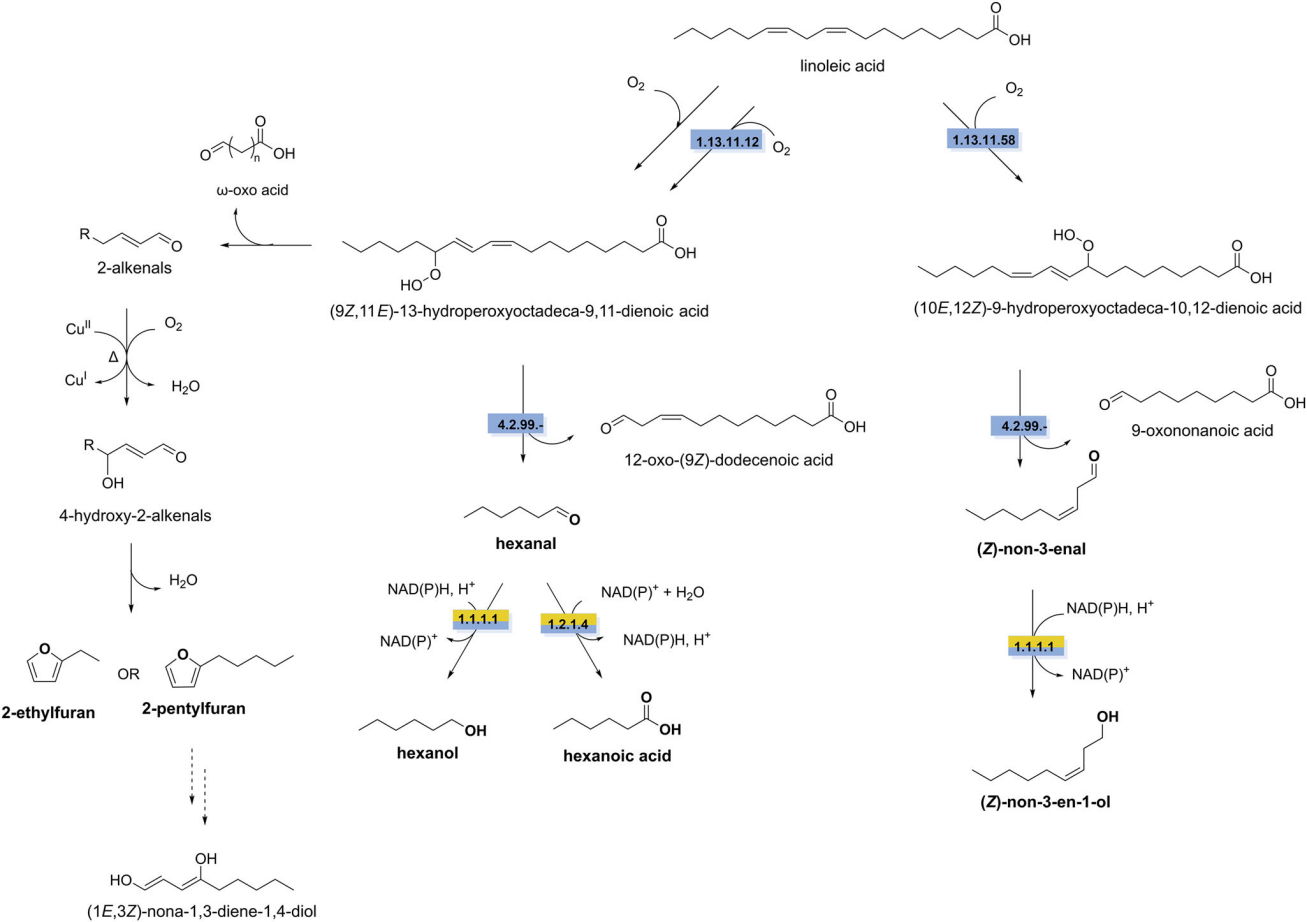
The synthesis and degradation pathways of off‐flavor furans, aldehydes, alcohols, and acids (in bold) are shown. EC numbers are given for the known enzymes involved in synthesis (blue) and degradation (yellow). Dashed arrows indicate unknown steps. EC 1.13.11.12: linoleate 13S‐lipoxygenase. EC 1.13.11.58: linoleate 9S‐lipoxygenase. EC 4.2.99.‐: hydroperoxide lyase. EC 1.1.1.1: alcohol dehydrogenase. EC 1.2.1.4: aldehyde dehydrogenase.

Furans are five‐membered heterocyclics, for which the two main off‐flavors are 2‐ethylfuran and 2‐pentylfuran, differing in their alkyl chain length from two to five carbons (Molina et al. 2025). Alkylfurans are described as having burnt like odors with odor thresholds of 2.3 and 0.6 ppb for 2‐ethylfuran and 2‐pentylfuran, respectively. The synthesis of alkylfurans in plant‐based protein isolates or concentrates is mostly due to non‐enzymatic reactions in the cell. Linoleic acid, the same compound that serves as a precursor for aldehydes, can undergo lipid peroxidation during light exposure in the presence of chlorophyll or after thermal treatments leading to the formation of hydroperoxide intermediates (Min et al. 2003). These unstable intermediates undergo oxidative cleavage to form α,β‐unsaturated aldehydes, with varying chain lengths (Adams et al. 2011). Under mild conditions these molecules are the predominant volatiles formed. However, at higher temperatures (>100°C) and in the presence of metal catalysts, the alkenals can be hydroxylated at the γ‐carbon producing 4‐hydroxy‐2‐alkenals (Perez Locas and Yaylayan 2004). Subsequently, 4‐hydroxy‐2‐alkenals undergo cyclization and dehydration to form 2‐alkylfurans. The lengths of the alkyl tails determine if the produced compounds are 2‐ethylfuran or 2‐pentylfuran (Figure 1) (Grebenteuch et al. 2021). Although linoleic acid degradation is the predominant pathway for furan formation, two additional reactions can also contribute. Maillard reactions between reducing sugars and amino acids generate dicarbonyl intermediates that can cyclize to alkylfurans. Additionally, ascorbic acid degradation produces sugar‐like dicarbonyls, which can undergo cyclization and dehydration to form furans during heating (90–120°C) (Grebenteuch et al. 2021; Limacher et al. 2007). Furans are widely present in plant‐based foods and beverages. For example, pea protein isolate had on average 283.7 ± 54.8 µg/L of 2‐pentylfuran and 3.1 ± 0.8 µg/L of 2‐methylfuran (Zipori et al. 2024). Similarly, in another study, a plant protein isolate from pea was reported to have on average 304.3 µg/L of 2‐pentylfuran and 77.7 µg/L of 2‐ethylfuran (El Youssef et al. 2020). Several other furans were also reported for other plant protein isolates, including 2‐(1‐pentenyl)‐furan, tetrahydrofuran, 2,4‐dimethylfuran, 2‐methylfuran, and 2‐butylfuran; however, sensory data is missing (Nugroho et al. 2024).

### 4.2 Degradation of Furans

Research on the biodegradation of alkylfurans is still limited. To date, to our knowledge, only one study has described the degradation of 2‐pentylfuran in *S. cerevisiae*. The authors proposed that the pathway starts with a ring‐opening reaction catalyzed by an epoxide hydrolase, forming (1*E*,3*Z*)‐nona‐1,3‐diene‐1,4‐diol (Figure 1) (Xu et al. 2022). This is followed by a sequential alcohol oxidation catalyzed by an ADH yielding 4‐oxononanal. However, this pathway was solely based on GC–MS analysis; therefore, the biocatalysts could only be inferred based on the identified volatiles. Additional research is required to confirm this pathway. Another well‐studied furan degradation pathway, the Trudgill pathway, has been reported (Kakinuma and Yamatodani 1964). However, this involves the key intermediate furan‐2‐carboxylic acid, and there is currently no evidence that alkylfurans are metabolized via this route.

### 4.3 Degradation of Furans in Plant‐Based Products

Although furan degradation has not been well studied in plant‐based products, there are examples of a decrease in furan concentration after fermentation with food‐grade organisms. For example, *S. cerevisiae* could decrease the 2‐pentylfuran concentration in fermented algae (*Bangia fusco‐purpurea*) (Xu et al. 2022). Similarly, it was reported that the concentration of 2‐pentylfuran present in pea protein isolate could decrease on average from 304.3 to 46.4 µg/L when fermented by a commercial starter culture comprised LAB species. This community included *Bifidobacterium lactis*, *Lactobacillus acidophilus*, *Lactobacillus delbrueckii* ssp*. bulgaricus*, and *Streptococcus thermophilus* (El Youssef et al. 2020). Another study showed that several *Lactiplantibacillus plantarum* and *Lacticaseibacillus casei* (El Youssef et al. 2020) strains were able to reduce the concentration of 2‐ethylfuran and 2,4‐dimethylfuran when fermenting a mixed emulsion of pea, chickpea, and mung bean protein isolates (Engels et al. 2022). Additionally, it was found that generally heterofermentative LAB can reduce the concentration of several furans in almond, oat, pea, and potato to a greater extent than yeasts or homofermentative LAB (Nugroho et al. 2024).

Overall, the synthesis of furans is well known and there are numerous reports of microbial reduction of furans in plant‐based substrates, but mechanistic knowledge of specific enzymes, pathways, and the formed reaction products is currently missing. One study reports the ring opening of alkylfurans which could be the first step of alkylfuran metabolism.

## 5 Sulfur Compounds

### 5.1 Biosynthesis of Sulfur Compounds

**FIGURE 2:**
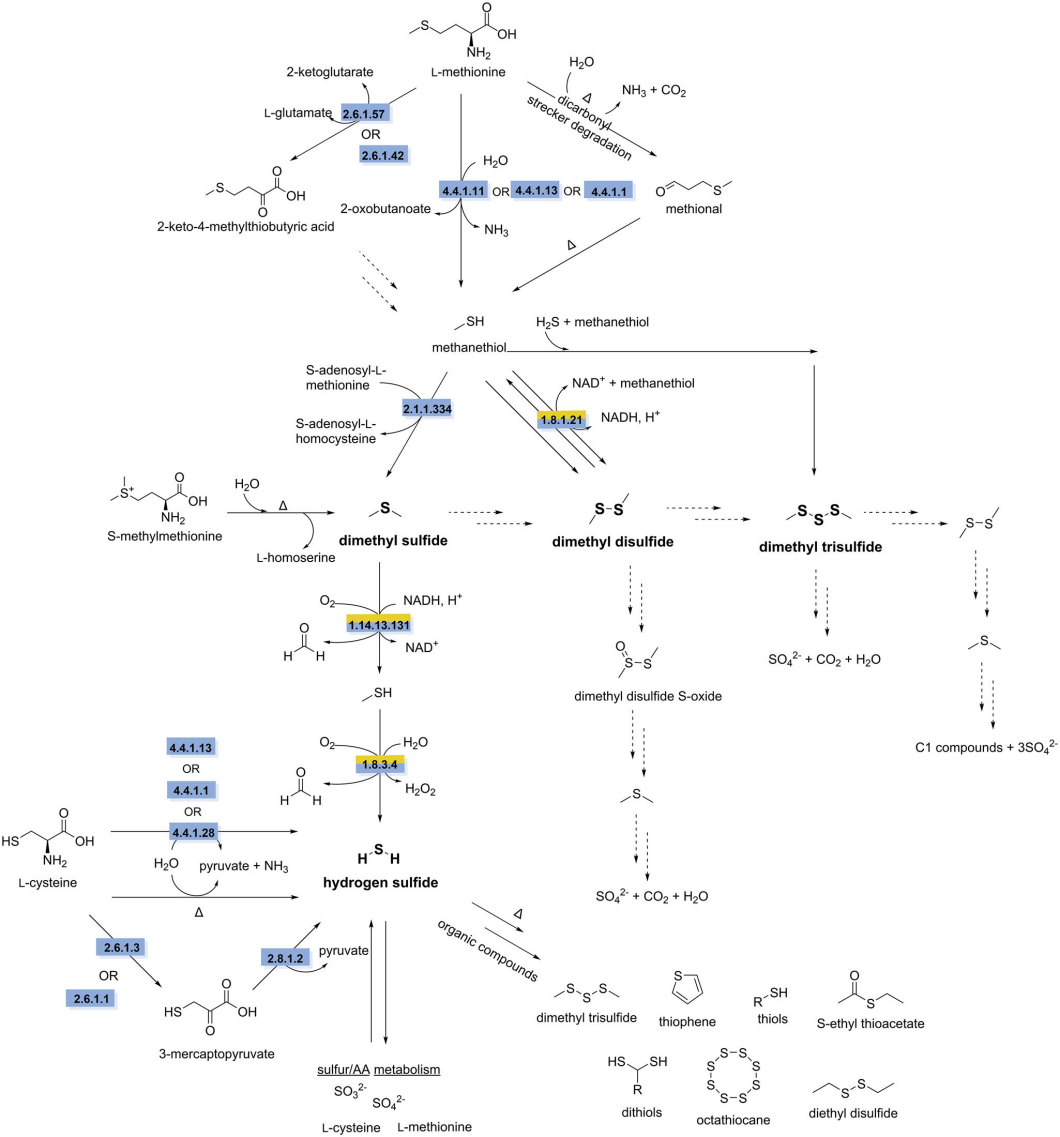
The synthesis and degradation pathways of off‐flavor sulfur compounds (in bold) are shown. EC numbers are given for the known enzymes involved in synthesis (blue) and degradation (yellow). Dashed arrows indicate unknown steps. EC 4.4.1.11: methionine γ‐lyase. EC 4.4.1.13: cysteine‐*S*‐conjugate β‐lyase. EC 4.4.1.1: cystathionine γ‐lyase. EC 2.6.1.57: aromatic‐amino‐acid transaminase. EC 2.6.1.42: branched‐chain‐amino‐acid transaminase. EC 2.1.1.334: methanethiol *S*‐methyltransferase. EC 4.4.1.28: l‐cysteine desulfidase. EC 2.6.1.1: aspartate transaminase. EC 2.6.1.3: cysteine transaminase. EC 2.8.1.2: mercaptopyruvate sulfurtransferase. EC 1.8.1.21: dimethyl disulfide reductase. EC 1.14.13.131: dimethyl sulfide monooxygenase. EC 1.8.3.4: methanethiol oxidase.

Methyl sulfides, such as dimethyl sulfide (DMS), dimethyl disulfide (DMDS), and dimethyl trisulfide (DMTS), are present in many foods, and they are important flavor molecules. For example, in cheeses they contribute to their characteristic flavor (Bonnarme et al. 2001). However, in plant‐based products, like plant‐based milk or protein isolates, these sulfur compounds are considered off‐flavors if they occur above their odor threshold of 0.005–12 ppb, contributing to a cabbage like odor (Leonard et al. 2023; Molina et al. 2025; Saffarionpour 2024). Methanethiol is the key precursor of methyl sulfides and is produced from the breakdown of methionine, which takes place via two distinct pathways. In one pathway, methionine is converted into 2‐oxobutanoate and methanethiol, catalyzed by methionine γ‐lyase (EC 4.4.1.11) (Tanaka et al. 1985). This reaction can also be catalyzed by the promiscuous activities of cysteine‐*S*‐conjugate β‐lyase (EC 4.4.1.13) and cystathionine γ‐lyase (EC 4.4.1.1), although with much lower efficiency compared to their primary reactions, the conversion of cystathionine to homocysteine and to cysteine, respectively (Alting et al. 1995; Dias and Weimer 1998; Dobric et al. 2000; Ono et al. 1992). An alternative route, and the predominant pathway for methanethiol production in cheese making, is the two‐step degradation of methionine via the intermediate 2‐keto‐4‐methylthiobutyric acid (KMBA). Conversion of methionine to KMBA is catalyzed by amino acid transaminases (EC 2.6.1.57 and EC 2.6.1.42) (Bonnarme et al. 2001; Dias and Weimer 1998; Kagkli et al. 2006). The degradation of KMBA to methanethiol is thought to occur enzymatically; however, the enzyme involved has not been identified (Amárita et al. 2001; Bonnarme et al. 2000). Methanethiol can then undergo methylation or condensation to form DMS or DMDS catalyzed by methanethiol *S*‐methyltransferase (EC 2.1.1.334) (Carrión et al. 2015) or DMDS reductase (EC 1.8.1.21) (Smith and Kelly 1988). Although the synthesis of DMDS can be enzymatic, DMDS synthesis is predominantly considered to be non‐enzymatic, occurring through spontaneous oxidation of methanethiol (Bosch et al. 2009; Chin and Lindsay 1994; van den Bosch et al. 2009). DMTS is synthesized chemically from the reaction of methanethiol with biologically produced sulfur particles (Figure 2) (Nedjma and Hoffmann 1996). Another sulfurous off‐flavor in food is hydrogen sulfide, which is known for its rotten‐egg‐like smell, with an odor threshold of 30 ppb in water. Hydrogen sulfide can be produced as a byproduct of the conversion of l‐cysteine to pyruvate catalyzed by l‐cysteine desulfidase (EC 4.4.1.28) in plants and cysteine‐*S*‐conjugate β‐lyase (EC 4.4.1.13) or cystathionine γ‐lyase (EC 4.4.1.1) in bacteria and fungi. l‐Cysteine can also be degraded through the intermediate 3‐mercaptopyruvate, catalyzed by aspartate transaminase (2.6.1.1) or cysteine transaminase (2.6.1.3). Subsequently, 3‐mercaptopyruvate sulfur transferase (EC 2.8.1.2) catalyzes the conversion of 3‐mercaptopyruvate to pyruvate, releasing hydrogen sulfide (Figure 2) (Birke et al. 2015; Kimura 2015; Nguyen et al. 2011; Yan et al. 2024).

### 5.2 Formation of Sulfur Compounds in Plant‐Based Products

Methyl sulfides and hydrogen sulfide are naturally synthesized in plants and can therefore accumulate in plant‐based products. However, food processing strategies also contribute to the accumulation of hydrogen sulfide and methyl sulfides. Fermentation of plant‐based products with LABs or yeast can lead to increased levels of methyl sulfides (Engels et al. 2022; Liu and Crow 2010; Nugroho et al. 2024). LAB like *Lacticaseibacillus casei* and *Lactococcus cremoris* (Dias and Weimer 1998), along with certain yeast species like *S. cerevisiae* (Bonnarme et al. 2001) and *Geotrichum candidum* (Berger et al. 1999), produce methanethiol, which can be further converted to methyl sulfides. Fermentation with *S. cerevisiae* is also known to increase hydrogen sulfide concentrations; however, this effect is strain dependent and has been studied primarily in wine and beer fermentations (Huang et al. 2017; Ugliano et al. 2011). Heat treatment plays an important role in the production of methyl sulfides, mainly through two different pathways. The degradation of *S*‐methyl methionine (SMM), a methionine derivative that naturally occurs in plants as part of their sulfur metabolism. SMM can break down into DMS during thermal processing of food (70–110°C) (Luo et al. 2018). Furthermore, it has been shown that heat treatment (>90°C) in soy milk promotes the Strecker degradation of l‐methionine, producing methional, which is then oxidized to methanethiol. Heating also accelerates the oxidation of methanethiol to DMDS and DMTS (Griffith and Hammond 1989; Lozano et al. 2007). Thermal processing also increases the concentration of hydrogen sulfide in products. Upon heating, cysteine degrades into pyruvate, ammonia, and hydrogen sulfide (Jo et al. 2019). The released hydrogen sulfide can react with other organic compounds to form various sulfur compounds, like DMTS, dithiol, thiophene, octathiocane, *S*‐ethyl thioacetate, diethyl disulfide, ethanethiol, and methanethiol (Figure 2) (Huang et al. 2017; Luo et al. 2024).

### 5.3 Degradation of Sulfur Compounds

The first microorganisms identified to degrade DMS and DMDS were *Thiobacillus* sp. and *Hyphomicrobium* sp. These methylotrophic bacteria can use DMS and DMDS as sole carbon and energy source, producing sulfite as an odorless end product (Boden et al. 2011; de Bont et al. 1981; Sivelã and Sundman 1975; Visscher et al. 1991). In their degradation pathway, DMDS and DMS are initially reduced to methanethiol by DMDS reductase (EC 1.8.1.21) (de Bont et al. 1981) and DMS monooxygenase (EC 1.14.13.131) (Smith and Kelly 1988). However, the gene encoding for DMDS reductase has not yet been identified. Subsequently, methanethiol is oxidized to hydrogen sulfide by methanethiol oxidase (EC 1.8.3.4) (de Bont et al. 1981; Eyice et al. 2018). An alternative pathway for DMDS degradation has been reported for *Bacillus cereus*, in which DMDS is initially oxidized to dimethyl disulfide S‐oxide (DMDSO), before further degradation into DMS yielding sulfate and carbon dioxide as end products (Figure 2) (Liang et al. 2015). Research on the degradation of DMTS is still limited. One study investigates the growth of *Pseudonocardia asaccharolytica* on DMTS as carbon source (Rappert and Müller 2005). During growth, researchers observed the accumulation of DMDS and DMS along with a stoichiometry of 3 mol sulfate per mol DMTS. This suggests that DMTS degradation proceeds through DMDS and DMS, producing sulfate as byproduct (Rappert and Müller 2005). However, the enzymes catalyzing the reactions remain unidentified. Two other bacteria have been reported to aerobically degrade DMTS. *Alcaligenes* sp. (Sun et al. 2016) oxidize DMS into DMDS and DMTS before further degradation to sulfate and carbon dioxide. *B. cereus* is reported to follow a similar pathway oxidizing DMDS to DMTS before further degradation to sulfate and carbon dioxide (Z. Liang et al. 2015). The intermediate steps and enzymes involved in these pathways are also unknown (Figure 2). Hydrogen sulfide can enter the sulfur metabolism to produce sulfite or sulfate; however, limited species are able to oxidize hydrogen sulfide, primarily sulfur‐oxidizing bacteria such as *Thiobacillus*, *Beggiatoa*, and *Paracoccus* (Pokorna and Zabranska 2015). Hydrogen sulfide can also be incorporated into the sulfur containing amino acids l‐cysteine and l‐methionine by various eukaryotic species, like *S. cerevisiae* (Huang et al. 2017).

### 5.4 Degradation of Sulfur Compounds in Plant‐Based Products

Many bacteria capable of metabolizing methyl sulfides have been isolated, but few are suitable for the food industry (Schafer et al. 2010). One study describes the degradation of DMS by the QPS‐listed bacterium *Bacillus licheniformis* (Anesti et al. 2005); however, this bacterium is not typically used in fermentations. The degradation of methyl sulfides during the fermentation of different plant‐based protein isolates has been observed for multiple yeast and LAB species, suggesting that a broader range of microbial species may be capable of breaking down or converting these compounds (Engels et al. 2022; Nugroho et al. 2024).

In summary, the synthesis of methyl sulfides and hydrogen sulfide from the amino acids l‐methionine and l‐cysteine is well characterized. The degradation of methyl sulfide into the off‐flavor hydrogen sulfide has been well characterized but not in food‐grade organisms. Enzymes that degrade methyl sulfides into sulfate are still uncharacterized but would be interesting targets to improve the odor of plant‐based products.

## 6 Alkyl‐Methoxypyrazines

### 6.1 Biosynthesis of Alkyl‐Methoxypyrazines

**FIGURE 3:**
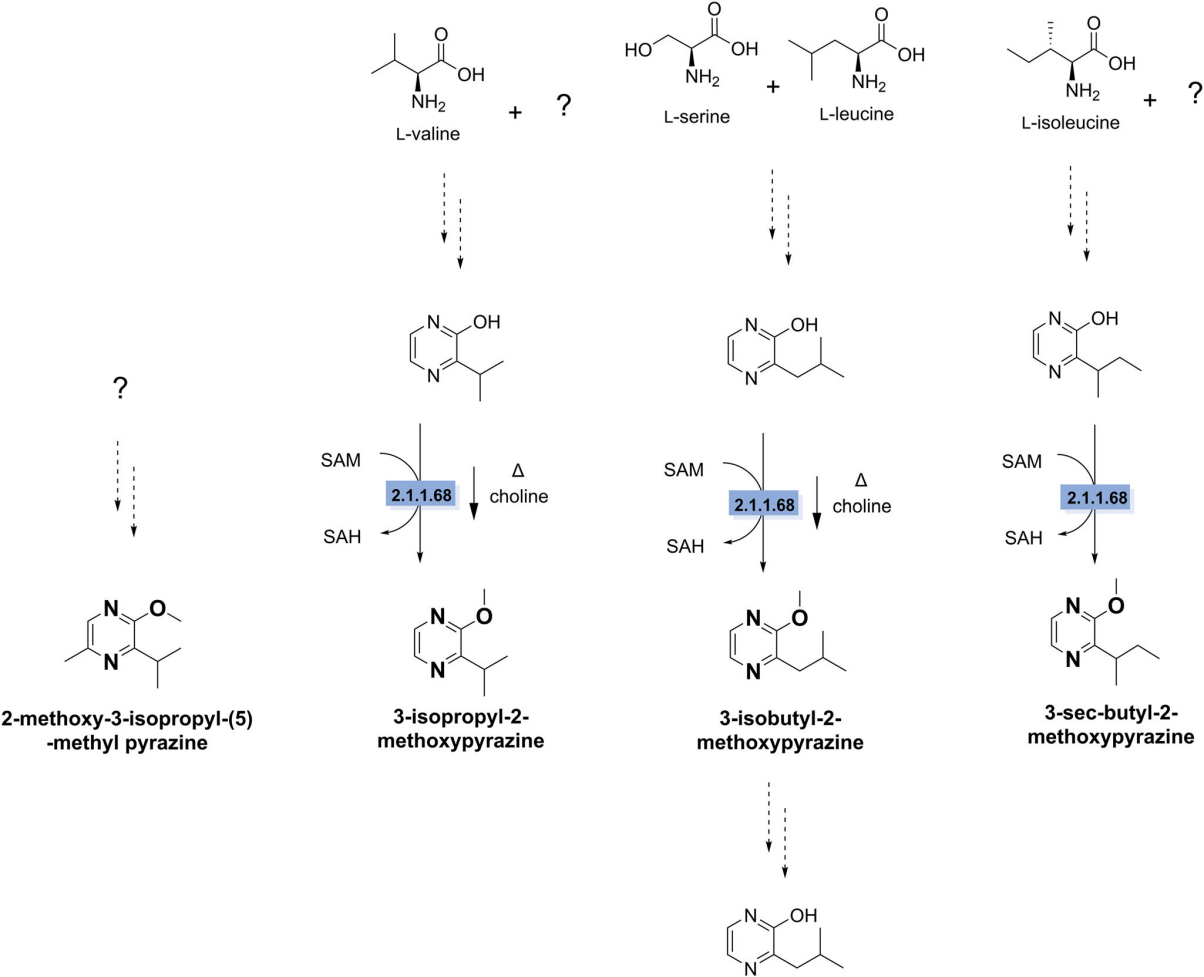
Biological synthesis of off‐flavor alkyl methoxypyrazines (in bold). The enzymes involved in synthesis (blue). Dashed arrows indicate unknown steps. Question marks indicate unknown substrates. EC 2.1.1.68: *O*‐methyltransferase.

In nature, pyrazines are known to act as signal molecules or repellents and can be synthesized by both plants and microorganisms. Although alkylpyrazines are valued for their roasted aromas produced through the Maillard reaction during heating, alkyl‐methoxypyrazines are considered off‐flavors, as they produce pea‐like, earthy, or bell pepper‐like odors. Alkyl‐methoxypyrazines are especially challenging because of their very low odor thresholds of 0.002 ppb (Saffarionpour 2024). The alkyl‐methoxypyrazines, 3‐isopropyl‐2‐methoxypyrazine (IPMP), 3‐isobutyl‐2‐methoxypyrazine (IBMP), and 3‐*sec*‐butyl‐2‐methoxypyrazine (sBMP), are the most common alkyl‐methoxypyrazines found in plant‐based products (Leonard et al. 2023; Murat et al. 2013; Roland et al. 2017; Saffarionpour 2024). Amino acids are the main precursors, and the side chain of methoxypyrazine is determined by the specific amino acid. For example, l‐valine is the precursor for IPMP, which was shown through labeling experiments in *Pseudomonas taetrolens* (Gallois et al. 1988). In bell pepper, similar labeling experiments showed that l‐leucine is the precursor for IBMP, and l‐serine is the second precursor (Zamolo and Wüst 2022). l‐isoleucine is believed to be the precursor for sBMP, as different sBMP compounds isolated from various vegetables, lady beetles, and grapes consistently have the same enantiomeric configuration as l‐isoleucine (Legrum et al. 2015). The second precursor for sBMP and IPMP has not been confirmed; however, research suggests it could be l‐glycine or l‐serine. So far, the only enzyme identified in the synthesis pathway is the one catalyzing the *O*‐methylation of the hydroxy group to form the methoxy group. This *O*‐methyltransferase (OMT, EC 2.1.1.68) has its highest activity with caffeic acid as a substrate, but it also methylates IPMP, IBMP, and sBMP in grapes (*Vitis vinifera*) (Dunlevy et al. 2010; Hashizume, Tozawa, Hiraga, et al. 2001, Hashizume, Tozawa, Endo, et al. 2001). Four OMTs (OMT1–OMT4) have been identified, each with different substrate affinities (Figure 3) (Dunlevy et al. 2010; Guillaumie et al. 2013). The synthesis of 2‐methoxy‐3‐isopropyl‐(6)‐methyl pyrazine, a pyrazine found in peas, contributed to hay‐like odor (Murat et al. 2013), has not been studied yet.

### 6.2 Formation of Alkyl‐Methoxypyrazines in Plant‐Based Products

Alkyl‐methoxypyrazines are naturally produced by plants and are co‐extracted with proteins during processing, leading to their accumulation in plant‐based protein sources (Buttery et al. 1969; Murat et al. 2013; Ryona et al. 2010). There is reason to believe that there is also a non‐enzymatic, thermal synthesis of methoxypyrazines. Research shows that roasting rapeseeds showed an increase in IPMP (Ortner et al. 2016), similar to the increase of IBMP when roasting pulses (Trikusuma et al. 2020; Vasundhara and Parihar 1981). However, limited research has been done on the non‐enzymatic route of alkyl‐methoxypyrazines in foods. One study describes the possible chemical methylation of alkyl‐hydroxypyrazines by the natural occurring methylating reagent choline; during thermal processing (180°C for 1 h), choline derived from soy lecithin can function as a methylating agent, converting hydroxypyrazines into methoxypyrazines but with a very low yield of only 0.14% (Rizzi 1990).

### 6.3 Degradation of Alkyl‐Methoxypyrazines

Currently, no biodegradation pathways have been described for methoxypyrazines. However, studies in rats suggest that IBMP is metabolized into IBHP through *O*‐demethylation after ingestion, although the enzymes responsible remain unidentified (Figure 3) (Hawksworth and Scheline 1975). Similarly, during maturation of wine grapes (*V. vinifera*), a decrease in IBMP levels corresponds proportionally with an increase in IBHP, implying that IBMP may be converted to IBHP as an initial step in the degradation process (Ryona et al. 2010). Some chemical strategies exist to reduce IPMP levels in drinking water, but they rely on nonspecific oxidation to degrade the methoxypyrazines. This approach is not ideal for food applications, as it can also break down desirable flavor compounds (Antonopoulou et al. 2020).

Thus, the information on biosynthesis and biodegradation of alkyl‐methoxypyrazines is still very limited. Because pyrazines can be perceived by humans at concentrations below the detection limits of standard analytical techniques, studying their conversion or degradation in plant‐based substrates is challenging.

## 7 Glycoalkaloids

### 7.1 Biosynthesis of GAs

GAs are composed of a nitrogen‐containing steroidal aglycone linked to at least one sugar moiety attached to the 3‐OH position (Han et al. 2025). The glycosidic side chain commonly includes monosaccharides such as glucose, galactose, rhamnose, or xylose. GAs are secondary metabolites in many plant families but are most abundant in *Solanaceae*. Over 90 structurally different GAs are found in *Solanum* spp., with potato (*Solanum tuberosum*), tomato (*Solanum lycopersicum*), and aubergine (*Solanum melongena*) being the main GA‐producing species (Osman 1983). The major GAs present in these species are α‐chaconine and α‐solanine in potato, α‐tomatine and dehydrotomatine in tomato, and α‐solamargine and α‐solasonine in aubergine (Han et al. 2025). GAs are associated with bitterness and a burning sensation in mouth and throat at concentrations of >20 mg/100 g DW in potato (Kondamudi et al. 2017). In potato, α‐chaconine is associated with highest toxicity compared to other potato GAs (Urugo and Tringo 2023); however, potato poisoning is likely caused by the combined effect of multiple GAs (Korpan et al. 2004). At lower doses, GAs may cause symptoms like vomiting and diarrhea, whereas at higher doses, they can lead to neurological disorders and can be lethal (Friedman and Levin 2016).

Cholesterol is the key precursor for GAs synthesis. Cholesterol undergoes several hydroxylation reactions catalyzed by cytochrome P450 monooxygenases and hydroxylation/oxidation of GA metabolism‐specific enzymes (GAMEs). Once the aglycone structure is formed, various sugars are attached to the 3‐OH position of the aglycone structure by UDP‐glycosyltransferases (UGTs) (Liu et al. 2024). For example, the biosynthesis of solasodine, the aglycone structure of several potato GAs such as α‐solasonine, solaradinine, solaradixine, and solashabanine, involves a series of oxidative and structural modifications of cholesterol. The enzymes GAME6/8 (EC 1.14.13.‐) and GAME11 (EC 1.14.11.‐) introduce hydroxyl groups at the C‐22, C‐26, and C‐16 positions of the steroidal backbone, respectively. These hydroxylation steps are followed by ring formation, catalyzed by GAME4 (EC 1.14.19.‐) and GAME12 (EC 2.6.1.‐), leading to the production of the aglycone solasodine. In the final step, glycosylation of the aglycone to form α‐solasonine is catalyzed by solanum SGT1, SGT2, and SGT3 (EC 2.4.1.‐) (Osman 1983; Sonawane et al. 2018).

### 7.2 Formation of GAs in Plant‐Based Products

GAs are naturally present in potatoes, with the highest concentrations found in the peel (1500–2000 mg/kg DW, green skin) and sprouts (2000–9970 mg/kg DW) (Omayio et al. 2016). GAs are often co‐extracted during the production of potato protein isolates and they end up in the final product. Moreover, processing conditions, such as exposure to light, heat, and mechanical damage, can further increase GA levels (Omayio et al. 2016). Additionally, long‐term storage at temperatures above 10°C leads to higher GA accumulation compared to cold storage (Haase 2010).

### 7.3 Degradation of GAs

**FIGURE 4:**
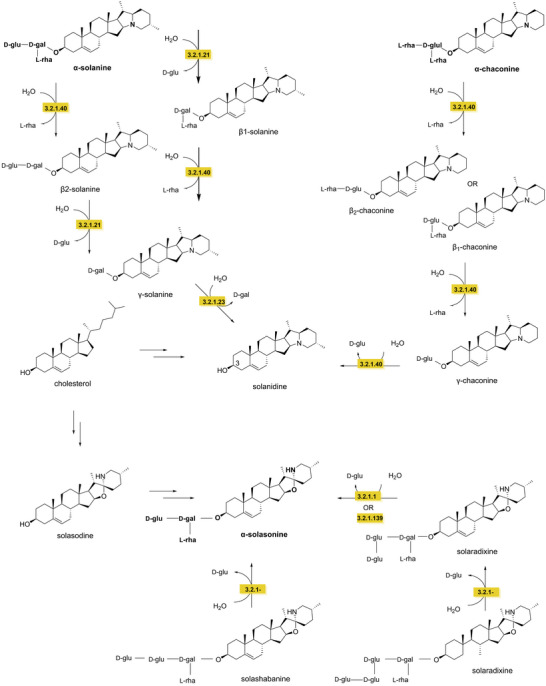
Biodegradation of the potato glycoalkaloids α‐chaconine and α‐solanine (in bold). EC numbers are given for the known enzymes involved in in biodegradation (yellow). EC 3.2.1.40: α‐rhamnosidase. EC 3.2.1.21: β‐glucosidase. EC: 3.2.1.23: β‐galactosidase. EC 3.2.1.‐: β‐glycosidase. EC 3.2.1.139: α‐glucuronidase. EC 3.2.1.1: taka‐diastase.

α‐Chaconine and α‐solanine are heat‐stable and only decompose at temperatures between 230°C and 280°C (van Gelder 1989). Detoxification of GAs typically involves the hydrolysis of glycosidic bonds, which also reduces bitterness (Cárdenas et al. 2019). α‐Rhamnosidase (EC 3.2.1.40), β‐glucosidase (EC 3.2.1.21), and β‐galactosidase (EC 3.2.1.23) characterized from *Glutamicibacter halophytocola* S2 (W. Wang, Du, et al. 2022) and *Arthrobacter* sp. S41 (Hennessy et al. 2020) degrade α‐chaconine and α‐solanine to the aglycone solanidine, via the formation of β‐/γ‐GA intermediates. Similarly, the biodegradation of solaradinine and solashabanine to α‐solasonine is catalyzed by β‐glycosidases (EC 3.2.1.‐), α‐glucuronidases (EC 3.2.1.139), and taka‐diastase (EC 3.2.1.1) (Figure 4) (Osman 1983).

### 7.4 Degradation of GAs in Plant‐Based Products

Recent research showed that the edible fungi *Flammulina velutipes* and *Pleurotus pulmonarius* can degrade both α‐chaconine and α‐solanine, offering a promising food‐grade fermentation strategy to reduce these compounds in plant‐based products. Fermentation with *F. velutipes* reduced solanidine content by 94% (Happel et al. 2025). During degradation, β‐/γ‐GA intermediates were not detected. Suggesting involvement of alternative enzymes and pathways rather than the typical sugar‐removal pathway (Happel et al. 2025).

All in all, the degradation of GAs is known to start with the cleavage of the sugar groups to from the aglycone catalyzed by glycosidases. However, glycosidases can be very specific to their substrate which means that not every organism that expresses glycosidases can degrade GAs. Additionally, the degradation of the aglycone structure remains unknown.

## 8 Pyrimidine Glycosides

### 8.1 Biosynthesis of Pyrimidine Glycosides

Faba beans contain two major pyrimidine glycosides, vicine and convicine, which are strongly associated with bitterness in faba bean products (Karolkowski et al. 2023). Vicine and convicine content ranges from 6–8 to 2–4 g/kg, respectively (Martineau‐Côté et al. 2022). Both compounds originate from the purine biosynthesis pathway, with guanosine triphosphate (GTP) as the key precursor. GTP is converted into the unstable intermediate 2,5‐diamino‐6‐ribosylamino‐4(3H)‐pyrimidinone 5′‐phosphate (DARPP) by the enzyme GTP cyclohydrolase II (encoded by *VC1*). DARPP is then deaminated to form 5‐amino‐6‐ribosylamino‐2,3(1H,3H)‐pyrimidinedione 5′‐phosphate (ARPDP). Through a series of reactions catalyzed by hydrolases, deaminases, and glycosyltransferases, vicine is synthesized from DARPP and convicine from ARPDP (Björnsdotter et al. 2021).

### 8.2 Degradation of Pyrimidine Glycosides

**FIGURE 5:**
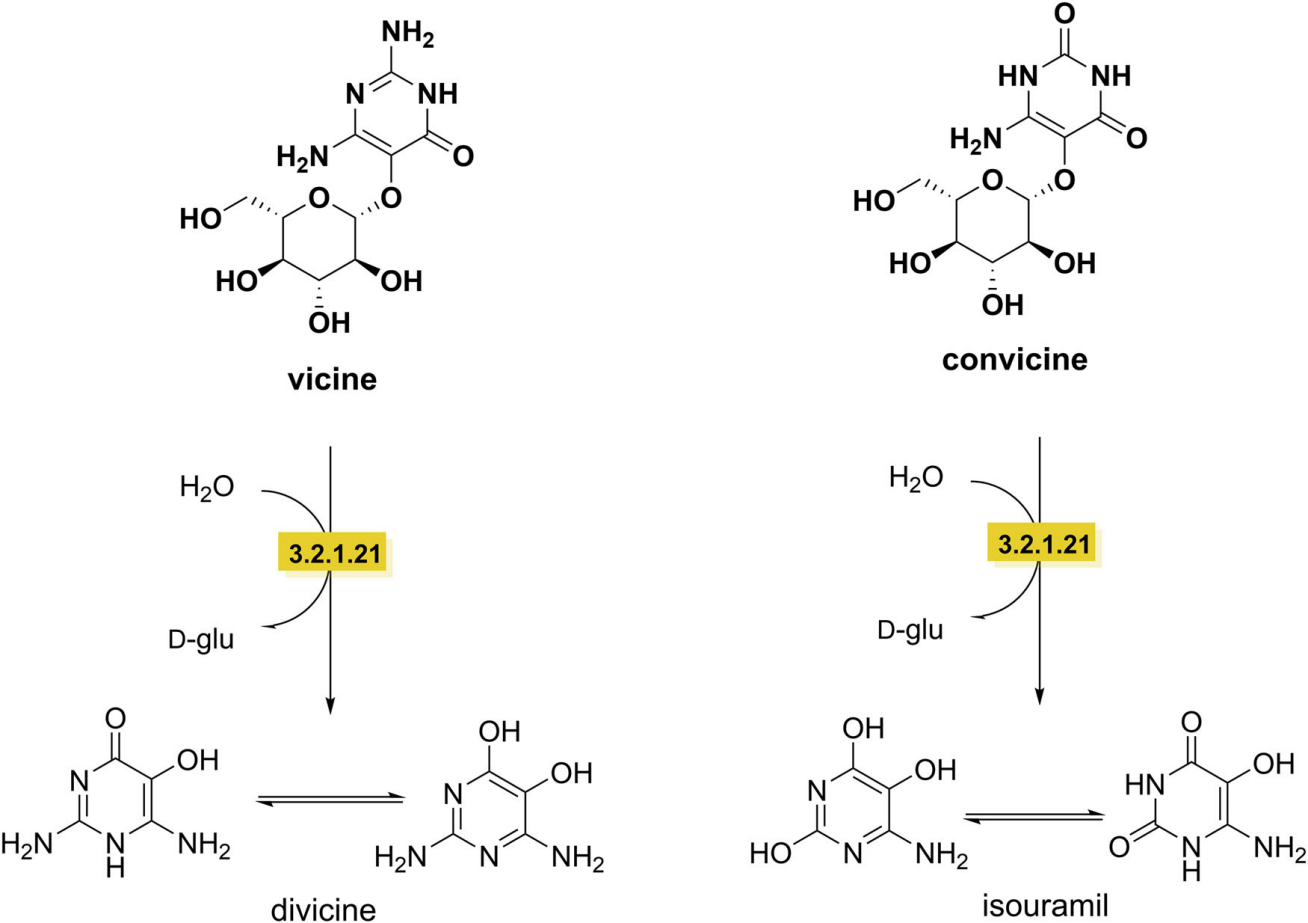
Degradation pathway of vicini and convicine (in bold) to their aglycones divicine and isouramil. EC 3.2.1.21: β‐glucosidase.

Degradation of vicine and convicine to their aglycones, divicine and isouramil, is catalyzed by β‐glucosidase (EC 3.2.1.21) in faba bean. The activity of β‐glucosidase increases from immature to ripe and decreases in older seeds. The aglycones are considered most toxic, mainly through the production of reactive oxygen species, that can cause hemolytic anemia (favism) by destroying red blood cells (Sergeant et al. 2024). The food‐grade organism *Lactobacillus plantarum* DPPMAB24W (VTT E‐133328), with β‐glucosidase activity, degrades vicine and convicine by more than 90% during fermentation, with no detection of the aglycone structure (Rizzello et al. 2016). *Aspergillus oryzae* also showed β‐glucosidase activity, which resulted in the cleavage of sugar group of vicine and convicine (McKay 1992). However, these aglycone structures are unstable during fermentation conditions; their degradation is therefore likely to occur chemically. For example, these two aglycones disappeared almost completely during heat treatment (200°C, 15 min), or after 2‐h incubation at 37°C at a pH of 5 (Pulkkinen et al. 2016). The mechanism involved in the degradation of divicine and isouramil remains unknown, but most likely involves the oxygen dependent oxidation to break the pyrimidine ring (Figure 5) (Pulkkinen et al. 2016).

## 9 Saponins

### 9.1 Biosynthesis of Saponins

Saponins are secondary metabolites in many plant species, composed of either steroidal (C27) or triterpenoidal (C30) aglycones attached to one or more saccharides (Yu et al. 2022). Saponins are nonnutritive and bitter and therefore undesirable in plant‐based products (Kim et al. 2021). Soy, oat, and faba beans contain saponins. Although soy and faba beans contain mainly soyasaponins, oats contain saponins known as avenacosides and avenacins. Soyasaponins are mainly differentiated into four groups based on different aglycone structures, groups‐A, B, E, and 2,3‐dihydro‐2,5‐dihydroxy‐6‐methyl‐4*H* (DDMP) (Wu and Kang 2011). Recently, Sg‐6 saponins have been identified in wild soybeans from Japan and South Korea. These saponins contain soyasapogenols H, I, or J, each with a single sugar chain attached at the C‐3 position of the aglycone (Takahashi et al. 2017). Both group B and DDMP saponins are largely related to a bitter and astringent taste in the final products (Clay et al. 2009). The bitterness threshold is 2 and 8 mg/L in water for soyasaponin βg(VI) and soyasaponin Bb(I), respectively (Agerbink and Olsen 2015).

**FIGURE 6:**
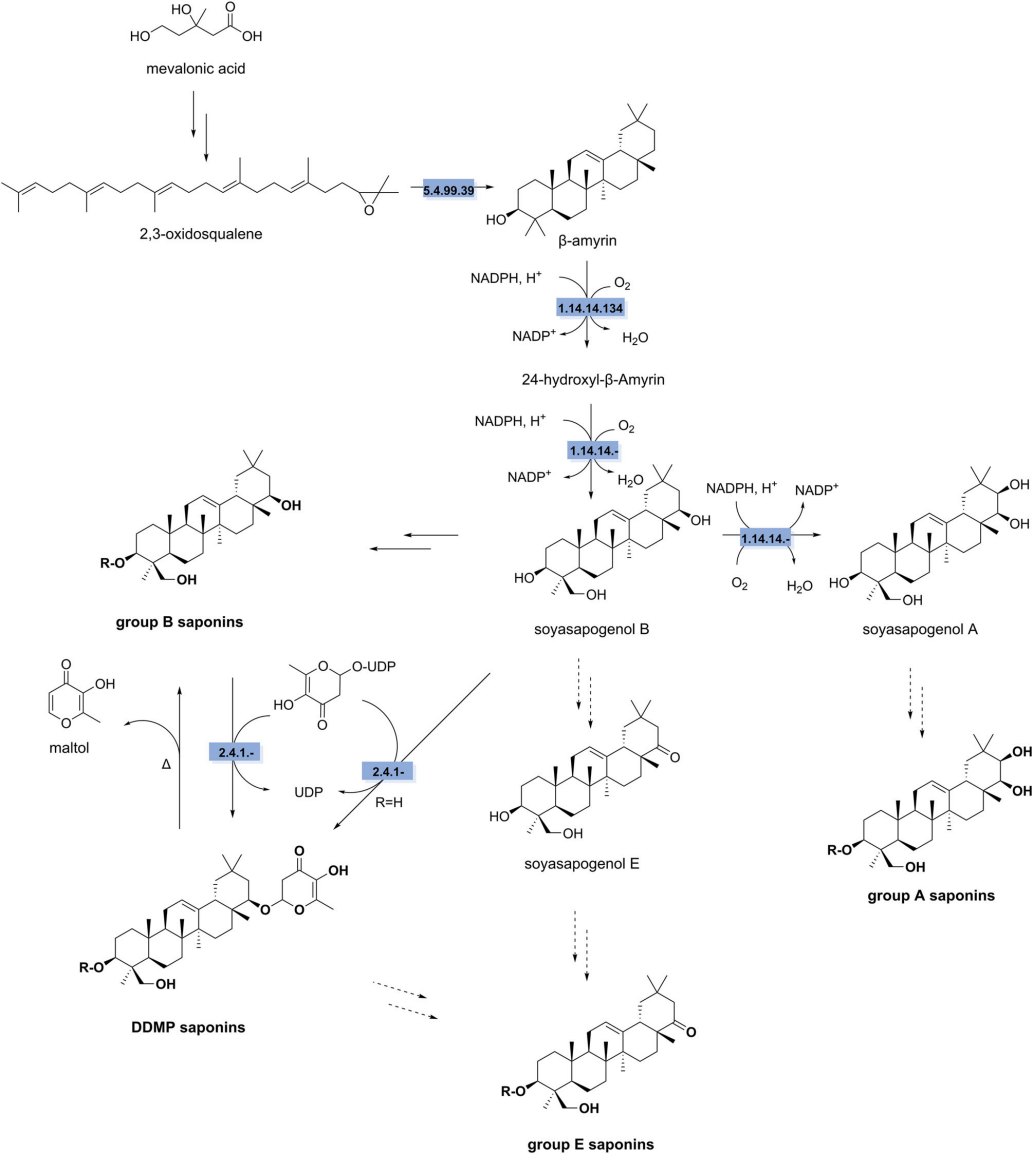
The synthesis and degradation pathways of soyasaponins (in bold) are shown. EC numbers are given for the known enzymes involved in synthesis (blue). Dashed arrows indicate unknowns steps. EC 5.4.99.39: β‐amyrin synthase. EC 1.14.14.134: β‐amyrin 24‐hydroxylase. EC 1.14.14.‐: cytochrome P450 CYP72A69. EC 2.4.1‐: UDP‐glycosyltransferase UGT73K5.

The key precursor of soyasaponins is 2,3‐oxidosqualene, which originates from the mevalonate pathway. 2,3‐Oxidosqualene is cyclized to form the base of the aglycone structures, β‐amyrin, by β‐amyrin synthase (EC 5.4.99.39). β‐Amyrin is hydroxylated by β‐amyrin 24‐hydroxylase (EC 1.14.14.134) at the C24 position to form 24‐hydroxyl‐β‐amyrin. After a second hydroxylation at the C22 position catalyzed by 11‐oxo‐β‐amyrin 30‐oxidase (EC 1.14.14.‐), soyasapogenol B is synthesized, which is the aglycone of group B saponins. Soyasapogenol A and E are synthesized from soyasapogenol B. Soyasapogenol A is synthesized by an additional hydroxylation at position C21 catalyzed by cytochrome P450 CYP72A69 (EC 1.14.14.‐). Soyasapogenol E is produced through the oxidation of the C22 hydroxy group to form a carbonyl group; however, the enzyme catalyzing this reaction remains unidentified. Sugar moieties are then attached to the aglycones by unknown UDP‐glycosyltransferases (UGTs) to form the groups A/B/E saponins. DDMP saponins are synthesized through the attachment of the DDMP group to the C21 hydroxy group, either from group B saponins or from soyasapogenol B, catalyzed by the UDP‐glycosyltransferase UGT73K5 (EC 2.4.1.‐) (Figure 6) (Chung et al. 2020; Sundaramoorthy et al. 2019; Yu et al. 2022).

**FIGURE 7:**
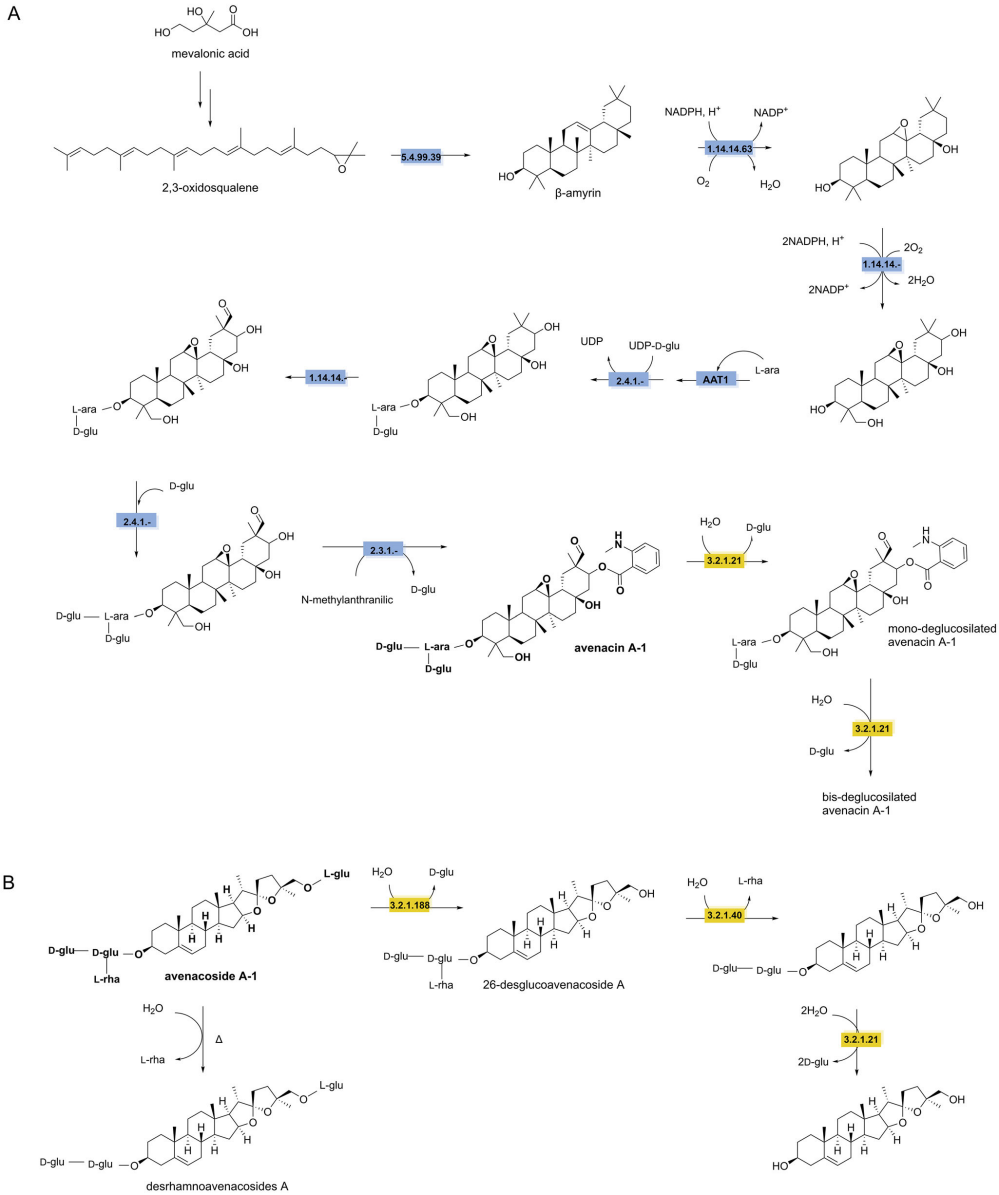
The synthesis and degradation pathways of oat saponins (in bold) are shown. EC numbers are given for the known enzymes involved in synthesis (blue) and degradation (yellow). EC 5.4.99.39: β‐amyrin synthase. EC 1.14.14.63: β‐amyrin monooxygenase. EC 1.14.14.‐: cytochrome P450 CYP72A475, CYP94D65, and CYP72A. EC 2.4.1.‐: UDP‐glycosyltransferases UGT91G16. EC 2.4.1.‐: transglucosidase. EC 2.3.1.‐: acetyltransferase. EC 3.2.1.31: β‐glucoronidase. EC 3.2.1.40: α‐rhamnosidase. EC 3.2.1.188: avenacosidase. EC 3.2.1.21: β‐glucosidase.

The synthesis of oat avenacin starts similarly to soyasaponins with the cyclization of 2,3‐oxidosqualene to β‐amyrin catalyzed by β‐amyrin synthase (EC 5.4.99.39). This is followed by the hydroxylation of the aglycone by β‐amyrin 16β‐monooxygenase (EC1.14.14.63). Cytochrome P450s (CYP72A475 and CYP94D65, EC 1.14.14.‐) catalyze the hydroxylation at the C21 and C23 positions, respectively. This is followed by the glycosylation of l‐arabinose to the 3OH position by AAT1. Subsequently, glucose is linked to l‐arabinose by a UDP‐glycosyltransferase (UGT91G16, EC 2.4.1.‐). This is followed by hydroxylation at the C30 position catalyzed possibly by cytochrome P450 (CYP72A476) and further oxidation to a carbonyl; however, these reaction steps are not well characterized. A second glucose molecule is linked to l‐arabinose by a transglucosidase (TG1/SAD3). Finally, *N*‐methylanthranilic acid is attached to C‐21 to form avenacin A (Figure 7). The synthesis of avenacosides is less understood but likely shares a similar biosynthetic pathway involving modifications of β‐amyrin to yield steroidal structures (Chung et al. 2020; Sundaramoorthy et al. 2019; Yu et al. 2022).

### 9.2 Biodegradation of Saponins

Biodegradation of soyasaponins has been described during fermentation or food processing. Fermentation has been applied to soy products to reduce saponin concentration to improve flavor and enhance bioavailability. In soy milk fermented with *Bacillus* BSNK‐5, saponin content decreased 1.8‐fold (Gao et al. 2022). *Aspergillus* strains can hydrolyze soybean saponins to their aglycones (Amin et al. 2011). Fermentation with *Lactobacillus rhamnosus* reduced group B soyasaponins while increasing soyasapogenol B due to high β‐glucosidase (EC 3.2.1.21) activity (Zhang et al. 2012).

DDMP saponins are relatively unstable and can be hydrolyzed to groups B and E saponins during food processing, extraction, or analysis. Recent studies suggest DDMP‐saponin degrades into groups B and E via heat treatment, peroxidation, and dehydrogenation, with the latter two reactions catalyzed by LOX isozymes in soybean seeds (Chitisankul et al. 2015). Cleavage of the DDMP group reduces the grassy, beany flavor of DDMP saponins while enhancing sweetness through maltol production (Figure 6). Acid treatment is the primary method for cleaving sugar moieties, but the choice of acid and solvent strongly affects the products. Prolonged hydrolysis in aqueous acid can produce soyasapogenols B1, C, D, and E, which are not naturally present in plants. However, hydrolysis with hydrochloric or sulfuric acid in anhydrous methanol selectively yields soyasapogenols A and B (Amin et al. 2011).

Oat saponins undergo biodegradation via microbial enzymes that hydrolyze sugar moieties sequentially, yielding more antifungal saponins. β‐Glucosidase (EC 3.2.1.21) characterized in *Gaeumannomyces graminis* var. *avenae* can produce mono‐/bis‐deglucosilated avenacin A‐1, through cleavage of the glucose groups (Osbourn et al. 1991). In plants during tissue disruption, endogenously expressed avenacosidase (EC 3.1.188) catalyzes the hydrolysis of avenacosides to 26‐desglucoavenacosides, which possess stronger antifungal activity (Gus‐Mayer et al. 1994; Yang et al. 2016). However, α‐l‐rhamnosidase (EC 3.2.1.40) isolated from *Stagonospora avenae*, hydrolyzes l‐rhamnose to detoxify the avenacoside (Bleddyn Hughes et al. 2004). Two different β‐glucosidase were also discovered in this species to cleave glucose groups attached to the C‐3 position (Morrissey et al. 2000). Additionally, processing conditions strongly influence oat avenacoside stability. Avenacosides A and B are stable at room temperature but are degraded (∼50% loss) at 140°C under acidic conditions, yielding desrhamnoavenacosides A and B through loss of l‐rhamnose. The sensory and toxicological properties of these degradation products remain unknown (Figure 7) (Sang and Chu 2017).

There remain several gaps in the saponin biosynthesis and biodegradation pathways. The biosynthetic pathways of specific saponins, for example avenacosides, are still unknown. Other pathways, like β‐amyrin synthesis, are well characterized; however, many of the enzymes responsible for attaching the sugar chains remain unidentified. Additionally, the biodegradation pathways of saponins are only partially understood, with limited knowledge of the final metabolic products, their taste, and toxicity. For oat saponins specifically, toxicity data are scarce (Önning and Asp 1995).

## 10 Polyphenols and Tannins

### 10.1 Biosynthesis of Polyphenols

Polyphenols are a large family of compounds consisting of at least one aromatic ring and at least one hydroxy group. Polyphenols are comprised several subclasses, such as flavonoids, phenolic acids, tannins, and smaller subclasses including curcuminoid, monolignols, and stilbenes. Polyphenols can affect the sensory profile of plant‐based foods, contributing to bitterness and astringency (Roland et al. 2017). For example, gallic, ferulic, sinapic, and coumaric acids have been linked to bitter, astringent, and sour flavor attributes (Duizer and Langfried 2016; Gaur and Gänzle 2024; Saffarionpour 2024).

**FIGURE 8:**
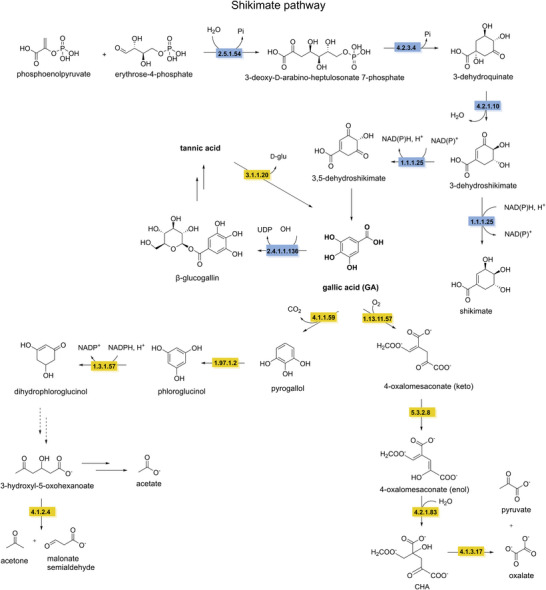
The synthesis and degradation pathways of tannic acid and gallic acid (in bold) are shown. EC numbers are given for the known enzymes involved in synthesis (blue) and degradation (yellow). Dashed arrows indicate unknown steps. EC 2.5.1.54: 3‐deoxy‐7‐phosphoheptulonate synthase. EC 4.2.3.4: 3‐dehydroquinate synthase. EC 4.2.1.10: 3‐dehydroquinate dehydratase. EC 1.1.1.25: shikimate dehydrogenase. EC 2.4.1.136: gallate 1‐β‐glucosyltransferase. EC 3.1.1.20: tannin acyl hydrolase (tannase). EC1.13.11.57: gallate dioxygenase. EC 5.3.2.8: 4‐oxalomesaconate tautomerase. EC 4.2.1.83: 4‐oxalomesaconate hydratase. EC 4.1.3.17: CHA aldolase. EC 4.1.1.59: gallate decarboxylase. EC 1.97.1.2: pyrogallol transhydroxylase. EC 1.3.1.57: phloroglucinol reductase. EC 4.1.2.4: aldolase.

Despite their high structural diversity, all polyphenols are derived from the shikimate pathway. This anabolic route starts with the condensation of phosphoenolpyruvate and erythrose‐4‐phosphate, catalyzed by 3‐deoxy‐d‐*arabino*‐heptulosonate 7‐phosphate synthase (EC 2.5.1.54) (Yokoyama et al. 2022). This condensation yields 3‐deoxy‐d‐*arabino*‐heptulosonate 7‐phosphate, which is then converted by 3‐dehydroquinate synthase (EC 4.2.3.4) into 3‐dehydroquinate. The third step in the shikimate pathway is catalyzed by 3‐dehydroquinate dehydratase (EC 4.2.1.10) producing 3‐dehydroshikimate. Subsequently, 3‐dehydroshikimate is converted to 3,5‐dehydroshikimate catalyzed by shikimate dehydrogenase (EC 1.1.1.25). Spontaneous tautomerization of 3,5‐dehydroshikimate produces gallic acid, the key building block for tannic acid. To biosynthesize hydrolyzable tannins, a glucose moiety is first added to gallic acid by gallate 1‐β‐glucosyltransferase (EC 2.4.1.136) forming β‐glucogallin. β‐Glucogallin is then used as substrate for a series of four highly regioselective galloylations, yielding 1,2,3,4,6‐pentagalloylglucose (Grundhöfer et al. 2001). The biosynthesis of tannic acid requires the addition of five more galloyl groups forming bonds with the existing galloyl groups, through several regioselective galloyl transferases (EC 2.3.1.‐) (Figure 8) (Niemetz and Gross 2005).

**FIGURE 9:**
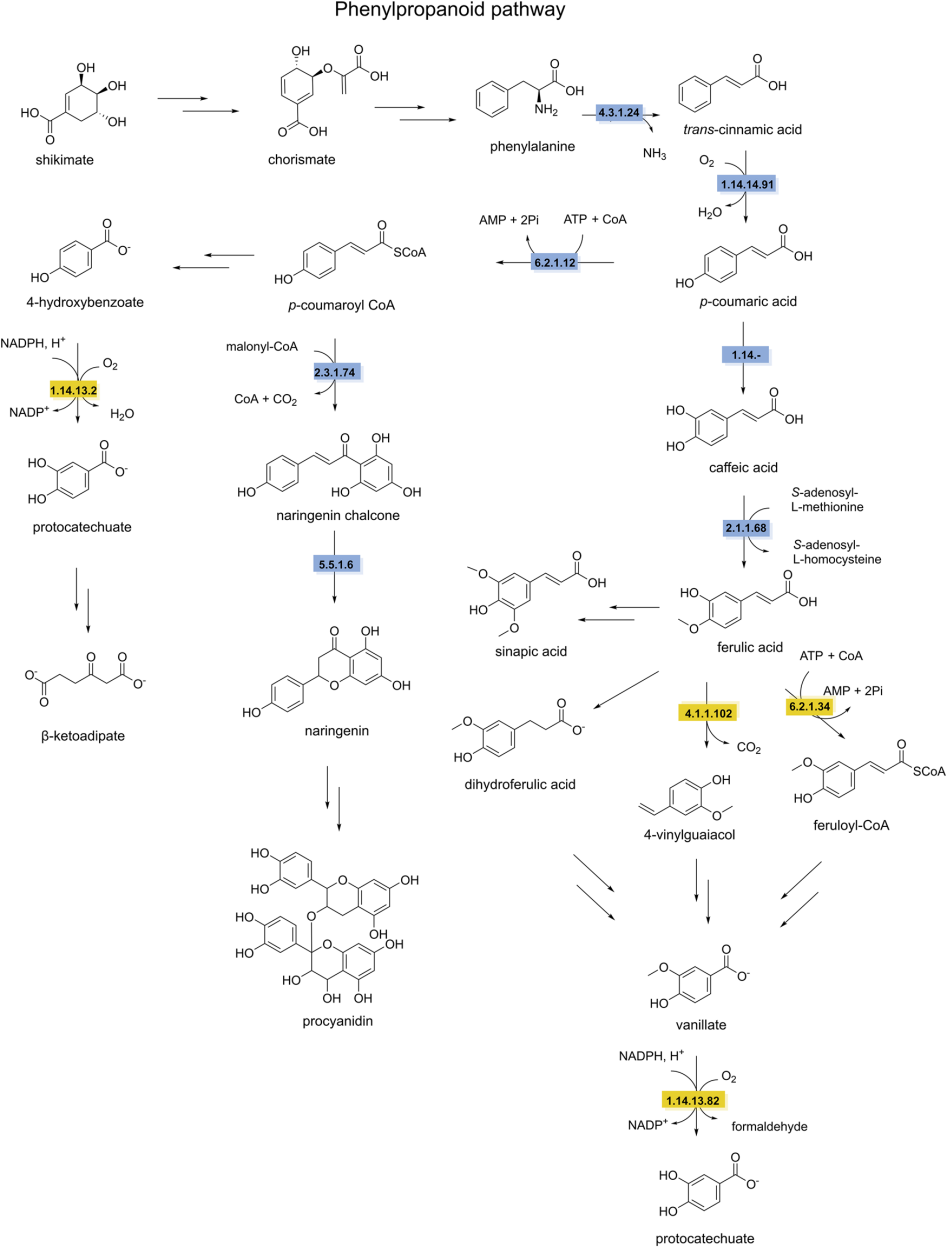
The synthesis and degradation pathways of polyphenols are shown. EC numbers are given for the known enzymes involved in synthesis (blue) and degradation (yellow). EC 4.1.3.24: phenylalanine ammonia‐lyase. EC 1.14.14.91: trans‐cinnamate 4‐monooxygenase. EC 1.14.‐: *p*‐coumarate 3‐hyrdoxylase. EC 2.1.1.68: caffeate *O*‐methyltransferase. EC 6.2.1.12: 4‐coumarate‐CoA ligase. EC 2.3.1.74: chalcone synthase. EC 5.5.1.6: chalcone isomerase. EC 1.14.13.2: 4‐hydroxybenzoate 3‐hydroxylase. EC 4.1.1.102: ferulic acid decarboxylase. EC 6.2.1.34: feruloyl‐CoA synthase. EC: 1.14.13.82: vanillate monooxygenase.

3‐Dehydroshikimate can also be reduced by shikimate dehydrogenase (EC 1.1.1.25) into shikimate. Subsequently, shikimate can be converted into chorismate through several enzymatic steps. Chorismate is an important precursor in the biosynthesis of aromatic amino acids, leading downstream to a wide array of phenolic natural products in both plants and microbes (Vogt 2010). The phenylpropanoid pathway is a second core pathway for the synthesis of polyphenols from phenylalanine. Phenylalanine is first deaminated by phenylalanine ammonia lyase (EC 4.3.1.24), generating *trans*‐cinnamic acid. Then, *trans*‑cinnamate 4‑monooxygenase (EC 1.14.14.91) hydroxylates *trans*‐cinnamic acid producing *p*‐coumaric acid. Hydroxycinnamic acids, such as caffeic, ferulic, and sinapic acids, are derived from *p*‐coumaric acid. The reactions leading to these polyphenols are catalyzed by *p*‐coumarate 3‐hydroxylase (EC 1.14.‐.‐) to introduce a hydroxyl group to yield caffeic acid, whereas caffeic acid 3‐OMT (EC 2.1.1.68) methylates the 3‐hydroxyl group of caffeic acid to produce ferulic acid. Ferulic acid can be further converted into sinapic acid. These diverse hydroxycinnamic acids serve as intermediates for the biosynthesis of a wide array of polyphenolic compounds in plants (Barros et al. 2019). Additionally, *p*‐coumaric acid can be activated, by 4‐coumarate‐CoA ligase (EC 6.2.1.12), producing *p*‐coumaroyl CoA, also a precursor for a wide range of polyphenols (Winkel 2006). In one pathway *p*‐coumaroyl CoA is converted to naringenin chalcone by chalcone synthase (EC 2.3.1.74). Followed by cyclization catalyzed by chalcone isomerase (EC 5.5.1.6), generating naringenin, a flavone (Figure 9). Several downstream enzymes lead to the synthesis of a wide array of aurones, flavones, isoflavonoids, flavonols, and anthocyanidins (Falcone Ferreyra et al. 2012).

### 10.2 Formation of Polyphenols in Plant‐Based Products

Plant‐based foods contain a diverse array of polyphenols which are described to have health benefits, varying among the subclasses (Bhuyan and Handique 2022). On the other hand, polyphenols also influence the sensory profile and color of plant‐based foods and beverages. Plants fermented by LAB tend to increase the total phenolic content, when compared with the raw material. This is due to the changes in the food matrix during fermentation, releasing polymeric and smaller monomeric polyphenols to the fermented solution (Wei et al. 2025). One of the challenges of polyphenols in plant‐based substrates is their ability to covalently bind to proteins. Phenolic compounds can be oxidized to *o*‐quinones, making them susceptible to Michael addition by nucleophilic protein groups (e.g., thiols or amino groups). The formation of *o*‐quinones can occur enzymatically or through auto‐oxidation, the latter taking place at alkaline pH or in the presence of oxidizing agents. In addition, quinone‐mediated condensation of monomeric phenolic compounds to their polymers is more reactive towards nucleophilic protein residues. The possible products of proteins and phenolic compounds interactions are endless. These complexes impact food color, techno‐functionality, and nutritional effects that are still not fully understood (Kieserling et al. 2024).

### 10.3 Degradation of Polyphenols

Biodegradation of hydrolyzable tannins differs between gallotannins and ellagitannins. The first step in the biodegradation of gallotannins is the hydrolysis of the ester bonds catalyzed by a tannin acyl hydrolase, commonly referred to as tannase (EC 3.1.1.20), resulting in the formation of gallic acid and glucose. The aerobic degradation of gallic acid has been described in *Pseudomomonas putida*, starting with the ring cleavage by a gallate dioxygenase (EC 1.13.11.57) to the keto form of 4‐oxalomesaconate. After a tautomerization step to the enol form of 4‐oxalomesaconate, carried out by a 4‐oxalomesaconate tautomerase (EC 5.3.2.8), the Zn^2+^‐dependent 4‐oxalomesaconate hydratase (EC 4.2.1.83) catalyzes the formation of 4‐carboxy‐4‐hydroxy‐2‐oxoadipic acid (CHA). The pathway converges into central metabolism through the formation of oxalate and pyruvate catalyzed by CHA aldolase (EC 4.1.3.17) (Nogales et al. 2011). In anaerobic conditions, gallate is decarboxylated to pyrogallol by gallate decarboxylases (EC 4.1.1.59). Pyrogallol is further degraded through the formation of the key phloroglucinol intermediate, catalyzed by a pyrogallol transhydroxylase (EC 1.97.1.2), which requires 1,2,3,5‐tetrahydroxybenzene as co‐substrate for the transfer of a hydroxyl group (Messerschmidt et al. 2004; Paizs et al. 2007). Its further degradation has been investigated in *Clostridia* and *Colinsella* sp. The proposed pathways start with reduction to dihydrophloroglucinol by NADPH‐dependent phloroglucinol reductase (EC 1.3.1.57), followed by ring cleavage to form (3*S*)‐3‐hydroxy‐5‐oxohexanoate that is further degraded to butyrate and then acetate through several enzymatic steps (Brune and Schink 1992; Conradt et al. 2016; Y. Li, Xu, et al. 2024; Zhou et al. 2023). An alternative pathway from (3*S*)‐3‐hydroxy‐5‐oxohexanoate has been recently proposed, starting with a C–C bond cleavage by an aldolase (EC 4.1.2.4) resulting in the production of acetone and malonate semialdehyde (Figure 8) (Y. Li, Xu, et al. 2024).

The first step in the degradation of ellagitannins is the hydrolysis of the ester bonds by tannase or ellagitannase (EC 3.1.1.124) leading to the release of hexahydroxydiphenic acid (HHDP) and glucose. HHDP then undergoes a spontaneous lactonization step forming ellagic acid following similar metabolic pathways described in the previous sections (Aguilera‐Carbo et al. 2008; De León‐Medina et al. 2025). Further degradation of ellagic acid has mainly been observed in the human gut microbiota (García‐Villalba et al. 2022). However, recent studies have shown that some enzymes involved in this degradation are expressed by the lactic acid bacterium *Limosilactobacillus fermentum* (Wang et al. 2025). The pathway begins with the cleavage of the lactone ring by a lactonase (EC 3.1.1.‐), the intermediate is then rapidly decarboxylated to form urolithin‐M5 (EC 4.1.1.‐), which then undergoes a series of dehydroxylation steps to form urolithin‐A.

The breakdown of complex polymeric structure of lignin leads to the formation phenolic alcohols and phenolic acids, such as ferulic and *p*‐coumaric acids, offering meaningful insights into possible degradation pathways for phenolic acids during fermentation of plant‐based food (Brink et al. 2019; del Cerro et al. 2021; Martim et al. 2024; Xu et al. 2019). Ferulic acid, which can follow different metabolic routes that can be (i) CoA‐dependent, with the conversion to feruloyl‐CoA by a feruloyl‐CoA synthase (EC 6.2.1.34); (ii) CoA‐independent, involving the non‐oxidative decarboxylation to 4‐vinylguaiacol, catalyzed by a ferulic acid decarboxylase (EC 4.1.1.102), or the side‐chain reduction to dihydroferulic acid, catalyzed by phenolic acid ene reductases (tentatively EC 1.3.1.‐ or related). These pathways converge towards the formation of vanillate through several enzymatic steps. Similarly, *p*‐coumaric acid, derived from H‐units degradation, can either be converted to a *p*‐hydroxycinnamoyl‐CoA intermediate by a 4‐coumaroyl‐CoA ligase (EC 6.2.1.12) or follow a CoA‐independent route involving the formation of 4‐hydroxybenzaldehyde catalyzed by a 4‐hydroxybenzaldehyde synthase, characterized in plants (EC 4.1.2.66) (Jung et al. 2016; Xu et al. 2019). *p*‐Coumaric acid degradation pathways result in the formation of *p*‐hydroxybenzoate. Both 4‐hydroxybenzoate and vanillate are further converted to protocatechuate by a 4‐hydroxybenzoate 3‐hydroxylase (EC 1.14.13.2) and a Rieske‐type vanillate‐*O*‐demethylase (EC 1.14.13.82), respectively. To enter the central metabolism, protocatechuate is further channelled into the β‐ketoadipate pathway, either directly or via an initial decarboxylation step to catechol catalyzed by a protocatechuate decarboxylase (EC 4.1.1.63) (Figure 9) (Fuchs et al. 2011; J. Li, Jiang, et al. 2024).

### 10.4 Degradation of Polyphenols in Plant‐Based Products

LAB and yeasts can catabolize tannins and release subunits of those polymeric polyphenols, such as gallic or ellagic acids (Leonard et al. 2021). This has implication in the sensory profile of the fermented products. Tannic acid is associated with astringency, whereas gallic acid, which is released during tannic acid hydrolysis, is associated with bitterness (Robichaud and Noble 1990). Therefore, the hydrolysis of tannic acid can lead to a reduction in astringency and increase in bitterness. Conversely, LAB species have been reported to reduce the content of ester‐type catechins, responsible for bitterness, whereas the other catechins related to mellow notes increased after tea fermentation (Mo et al. 2024). Likewise, ferulic acid present in a wheat sourdough can be converted into vinyl guaiacol, ethyl guaicol, and dihydroferulic acid by *L. plantarum*. The fermentation of plant‐based foods by LAB can also increase the bioavailability of polyphenols by improving the uptake of these compounds by Caco‐2 cells, when compared with unfermented plant matrixes (Zhao and Shah 2016).

As previously mentioned, gallic acid and other simple benzoic acid derivatives are key funnelling intermediates in the biodegradation of more complex polyphenols. Their central role makes them particularly relevant targets for studying complete microbial degradation pathways. Up to now, the only gallate decarboxylase (EC 4.1.1.59) characterized from a food‐grade organism is from *L. plantarum*, which catalyzes the decarboxylation of gallic acid to pyrogallol (Jiménez et al. 2013). This compound usually represents a final product, as its further degradation has not been described in food‐grade organisms (Jiménez et al. 2013).

Different Lactobacilli have shown degradation activity towards cinnamic acid derivatives such as *p*‐coumaric, caffeic, and ferulic acids (Gaur et al. 2020; Gaur and Gänzle 2024; Miyagusuku‐Cruzado et al. 2020; Mukai et al. 2010; Nogales et al. 2011). During fermentation with *S. cerevisiae* and LAB, these compounds can be decarboxylated to the corresponding vinylphenols by phenolic acid decarboxylases (EC 4.1.1.102). The resulting vinylphenols can be reduced to the corresponding ethyl phenols by vinyl phenol reductase. These compounds are generally described as having “clove” or “spicy” aromas and with less impact on profile (Leonard et al. 2023). Alternatively, the C = C double bond in the side chain of phenolic acids can be reduced by ene‐reductases (EC 1.3.1.‐), leading to the formation of the corresponding dihydroacids.

Overall, the biosynthetic pathways of polyphenols in plants are well elucidated for core subclasses. On the other hand, the biodegradation of these compounds is still in its early stages. The degradation of lignin and monolignols has been the central group of polyphenols studied, due to their economic and ecological relevance. Conversely, the degradation pathways of polyphenols relevant for the food industry have been mostly overlooked.

## 11 Glucosinolates

### 11.1 Biosynthesis of GSLs

GSLs consist of a core structure containing glucose, sulfur, and nitrogen, with a variable side chain originating from different amino acids. GSLs are responsible for an astringent and bitter taste associated with the *Brassicaceae* family, such as cabbages and oilseeds. GSL breakdown products, isothiocyanates (ITCs), are responsible for the pungent flavors or earthy aromas (Bell and Wagstaff 2017). Additionally, GSLs and their degradation products, ITCs, reduce the nutritional value of a product (Alseekh et al. 2021; Mithen et al. 2000).

**FIGURE 10:**
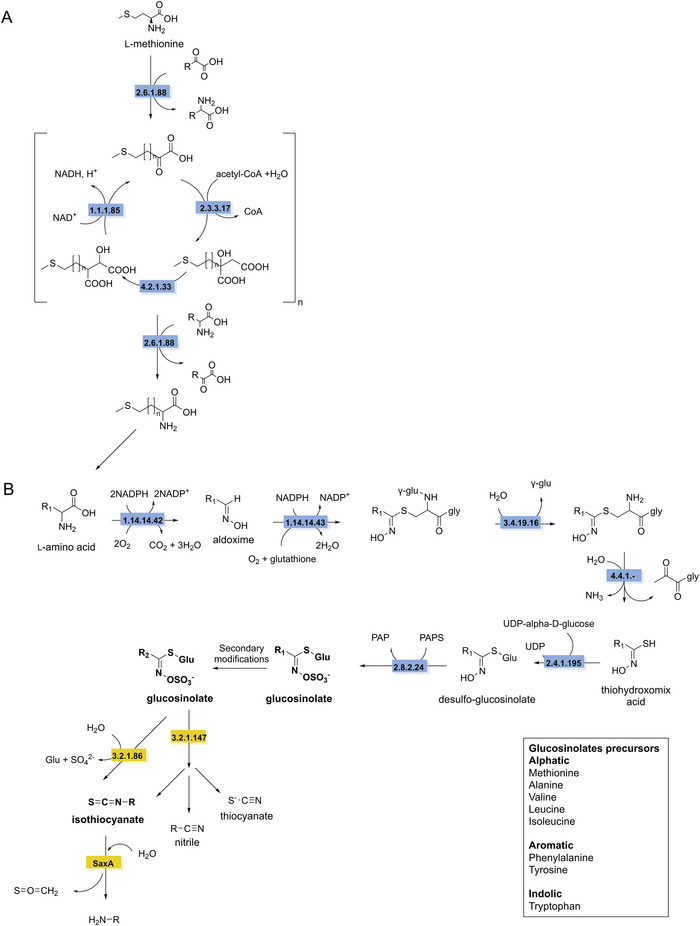
The synthesis and degradation pathways of glucosinolates (in bold) are shown. EC numbers are given for the known enzymes involved in synthesis (blue) and degradation (yellow). Chain elongation machinery shown for l‐methionine (A). Synthesis of the core glucosinolate structure (B). EC 2.6.1.88: methionine transaminase. EC 2.3.3.17: methylthioalkylmalate synthase. EC 4.2.1.33: 3‐isopropylmalate dehydratase. EC 1.1.1.85: 3‐isopropylmalate dehydrogenase. EC 1.14.14.42: homomethionine *N*‐hydroxylase. EC 1.14.14.43: (methylsulfanyl)alkanaldoxime *N*‐monooxygenase EC 3.4.19.16: γ‐glutamyl hydrolase. 4.4.1.‐: C–S lyase. EC 2.4.1.195: *S*‐β‐glucosyltransferase. EC 2.8.2.24: desulfoglucosinolate sulfotransferase. EC 3.2.1.147: myrosinase. EC 3.2.1.86: 6‐phospho‐β‐glucosidase. SaxA: isothiocyanate hydrolase. PAPS: 3′phospoadenylyl sulfate. PAP: adenosine 3′,5′‐bisphosphate.

GSLs can be classified, depending on their side chain, into aliphatic (derived from l‐alanine, l‐leucine, l‐isoleucine, l‐methionine, and l‐valine), benzenic (derived from l‐phenylalanine and l‐tyrosine), and indolic (derived from l‐tryptophan) (Sønderby et al. 2010). The biosynthesis of GSLs in plants has been investigated in depth in the model organism *Arabidopsis thaliana* and was found to comprise three independent stages: (i) chain elongation of the precursor amino acids (Figure 10A), (ii) formation of the core GSLs structure, and (iii) secondary modification of the side‐chain which is responsible for the variety of GSLs classes and subclasses (Figure 10B) (Fahey et al. 2001; Sønderby et al. 2010).

Elongation of the amino acid side chain begins with the deamination of an amino acid to form a 2‐oxo acid, catalyzed by branched‐chain amino acid aminotransferase (EC 2.6.1.88). The resulting 2‐oxo acid enters the elongation cycle, where it undergoes condensation with acetyl‐CoA catalyzed by methylthioalkylmalate synthase (EC 2.3.3.17). This is followed by an isomerization step mediated by isopropylmalate isomerase (EC 4.2.1.33), and finally, oxidation of the hydroxy group catalyzed by isopropylmalate dehydrogenase (EC 1.1.1.85) resulting in the 2‐oxo acid elongated by one methylene group (Chen et al. 2021; Sønderby et al. 2010; Textor et al. 2004). The number of cycles determines the length of the amino acid side chain (Figure 10A).

The biosynthesis of the core GLS structure begins with the conversion of amino acids, including chain‐elongated derivatives, into their corresponding aldoximes. This step is catalyzed by cytochrome P450 *N*‐monooxygenases (e.g., EC 1.14.14.43 for methionine‐derived substrates). Next, the aldoximes are oxidized to reactive intermediates (nitrile oxides or *aci*‐nitro compounds) by cytochrome P450 enzymes, such as methylsulfanylalkanaldoxime *N*‐monooxygenase (EC 1.14.14.43 for methionine‐derived aldoximes). These intermediates are then conjugated to glutathione (GSH). Following conjugation, the γ‐glutamyl group of the GSH moiety is cleaved by a γ‐glutamyl hydrolase (EC 3.4.19.16). The remaining C–S bond is then cleaved by a C–S lyase (EC 4.4.1.‐), resulting in the formation of a thiohydroximic acid. Thiohydroximic acid undergoes *S*‐glycosylation catalyzed by *S*‐β‐glucosyltransferase (EC 2.4.1.195) forming desulfoglucosinolates. The final step in the biosynthesis of the core structure is the sulfation by desulfoglucosinolate sulfotransferase (EC 2.8.2.24) to form the GSLs (Geu‐Flores et al. 2009; Piotrowski et al. 2004). Additionally, GSLs can undergo a variety of secondary modifications. These include oxygenations, hydroxylations, alkenylations, or benzoylations, contributing to their structural diversity (Figure 10B) (Sønderby et al. 2010).

### 11.2 Production of GSLs in Plant‐Based Products

GSLs are naturally occurring compounds found in plant species with varying total content depending on the plant species. In *Brassica rapa* species, GSLs content, among 113 *B*. *rapa* cultivars, ranges from 11.8 to 74 µmol/g (Padilla et al. 2007). In *Brassica napus*, the total GSLs content, among 33 cultivars, varied from 14 to 24 µmol/g (Padilla et al. 2007). In addition to the differences in GSLs content among crop species, particular classes of GSLs are predominant in a specific crop. For example, rapeseed meal contains high levels of aliphatic GSLs with sinigrin being the most abundant GSLs at 6 µmol/g (Xie et al. 2022).

### 11.3 Degradation of GSLs

GSLs are naturally present in plants and synthetized as part of the plant's defense system against pathogens and herbivores (Clay et al. 2009; Textor and Gershenzon 2009). GLSs are translocated to edible tissues and seeds (Nour‐Eldin et al. 2012; Xu et al. 2023). When plant tissues are damaged (e.g., during chewing or food preparation), GSLs are degraded by endogenous myrosinases (EC 3.2.1.147) into the bitter active compounds ITCs, nitrile, thiocyanate, or cyclic compounds (Agerbirk and Olsen 2015; Angelino et al. 2015). GSLs can also be degraded into ITCs by microorganisms. For example, *Companilactobacillus farciminis* metabolizes sinigrin in the absence of glucose through the activity of a 6‐phospho‐β‐glucosidase (EC 3.2.1.86) (Watanabe et al. 2021). Many of these ITCs have herbicidal and antimicrobial activities. Interestingly, some insect species, such as the diamond back moth (*Plutella xylostella*) and the desert locusts (*Schistocerca gregaria*), express GSL sulfatases in their digestive systems. These sulfatases convert GLSs to their desulfonated forms, preventing the hydrolysis of GLSs to form ITCs (Falk and Gershenzon 2007; Ratzka et al. 2002). Another strategy for ITC detoxification involves their enzymatic degradation. The plant pathogen *Pectobacterium* sp., which infects a wide range of crops, produces an enzyme called ITC hydrolase that catalyzes the breakdown of 2‐phenylethyl ITC into carbonyl sulfide and phenylethylamine (Welte et al. 2016). Homologous *saxA* genes have been identified in the genomes of 41 other bacterial species. These SaxA (ITC hydrolases) homologs showed varying levels of activity depending on the side chain structure of the ITC being degraded (Figure 10B) (van den Bosch et al. 2018).

### 11.4 Degradation of GSLs in Plant‐Based Products

Food processing, such as cooking, affects GSL degradation by inactivating myrosinase, thereby reducing ITC formation (Deng et al. 2015). In rapeseed seeds, roasting decreased the GSLs content by 20%–30% (Jing et al. 2020). GSLs can also be degraded by microorganisms. For example, fermentation of rapeseed meal by *Lactiplantibacillus pentosus* reduced ITCs by 60% after 24 h (Chen et al. 2024). A mixed culture of *Erwinia tasmaniensis, Enterococcus gallinarum, Bacillus subtilis*, and *L. plantarum* decreased GSLs content by 86% (Hong et al. 2025). *Aspergillus terreus* and the thermophilic fungus *Lichtheimia* sp. JN3C reduced GSLs content by 96% after 96 h in rapeseed meal (Hong et al. 2025). However, the specific enzymes responsible for GSL and ITC degradation during these fermentations remain unknown. Although extensive research has focused on the biosynthesis of GSLs and ITCs, only one enzyme (SaxA) has been characterized for ITC degradation. Discovering such enzymes in food‐grade microbes could be valuable for improving seed‐derived protein isolates.

Overall, the biosynthesis and degradation of GSLs are well established. However, identifying ITC hydrolases in food grade organisms could be useful to improve flavor of seed products.

## 12 Phytic Acid

### 12.1 Biosynthesis of PA

**FIGURE 11:**
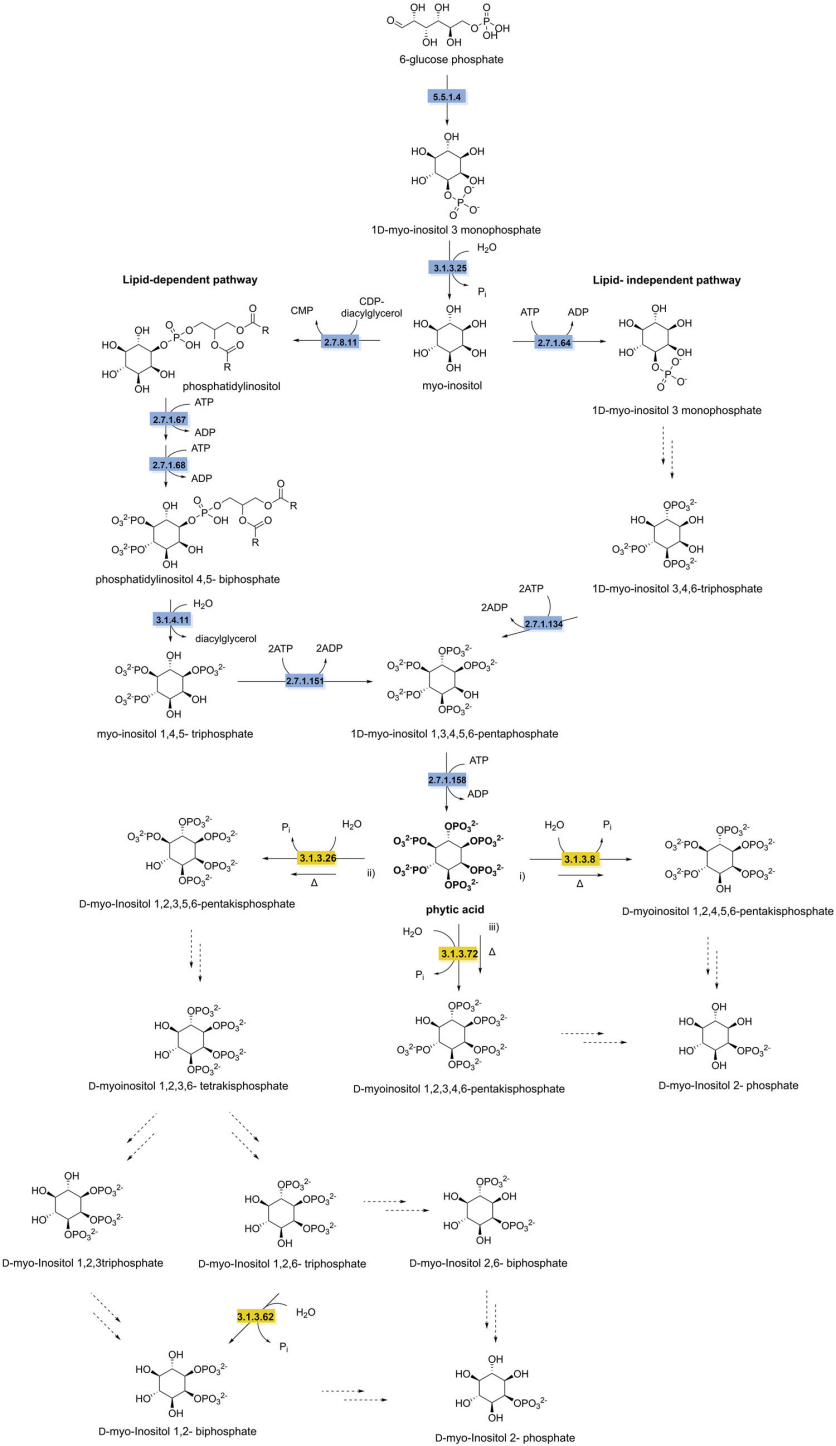
The synthesis and degradation pathways of phytic acid (in bold) are shown. EC numbers are given for the known enzymes involved in synthesis (blue) and degradation (yellow). Dashed arrows indicate unknown steps. EC 5.5.1.4: *myo*‐inositol‐3‐P1 synthase. EC 3.1.3.25: inositol monophosphatase. EC 2.7.8.11: phosphatidylinositol transferase. EC 2.7.1.67: phosphatidylinositol 4‐kinase. EC 2.7.1.68: phosphatidylinositol 4‐phosphate 5‐kinase 7. EC 3.1.4.11: phosphoinositide phospholipase C. EC 2.7.1.151: inositol‐polyphosphate multikinase. EC 2.7.1.64: inositol 3‐kinase. EC 2.7.1.134 inositol 1,3,4‐triskisphosphate 5‐6‐kinase EC 2.7.1.158: inositol‐pentakisphosphate 2‐kinase. EC 3.1.3.8: 3‐phytases. EC 3.1.3.72: 5 phytases. EC 3.1.3.26: 4/6 phytases. EC 3.1.3.62: multiple inositol‐phosphate phosphohydrolase.

PA, or *myo*‐inositol 1,2,3,4,5,6‐hexakisphosphate, is a phosphorus‐containing compound that serves as the primary phosphorus storage in seeds (Pramitha et al. 2021). It is most abundant in cereals, oilseeds, and nuts (Silva et al. 2021). Due to its strong negative charge, PA binds to divalent cations such as Fe^2+^ and Ca^2+^, thereby reducing the availability of these minerals, making it an antinutritional factor for humans (Chen and Xu 2023). PA biosynthesis starts with the conversion of 6‐glucose phosphate to *myo*‐inositol monophosphate in the position 3 (Ins(3)P_1_) catalyzed by *myo*‐inositol‐3‐P1 synthase (EC 5.5.1.4) (Suzuki et al. 2007). Ins(3)P_1_ is hydrolyzed to *myo*‐inositol (Ins) and inorganic phosphorous by Ins monophosphatase (EC 3.1.3.25) (Raboy 2009). Ins can subsequently enter one of two distinct biosynthetic routes for PA synthesis (i) lipid‐independent and (ii) lipid dependent pathway. The lipid dependent pathway is the primary route for PA synthesis in vegetative tissue of plants (Raboy 2009; Silva et al. 2021). This pathway begins with the formation of phosphatidylinositol (PtdIns), a lipid containing inositol in its headgroup, catalyzed by phosphatidylinositol transferase (EC 2.7.8.11). PtdIns is then phosphorylated to PtdIns(4,5)P_2_ catalyzed by 1‐phosphatidyl inositol 4‐kinase (EC 2.7.1.67) and catalyzed by 1‐phosphatidylinositol 4‐phosphate 5‐kinase (EC 2.7.1.68). PtdIns(4,5)P_2_ is subsequently converted to Ins(1,4,5)P_3_ by phosphoinositide phospholipase C (EC 3.1.4.11) which is further phosphorylated by inositol‐phosphate multikinase (EC 2.7.1.151), producing Ins(1,3,4,5,6)P_5_. Finally, Ins(1,3,4,5,6)P_5_ is phosphorylated by inositol polyphosphate 2‐kinases (EC 2.7.1.158) to produce PA (Figure 11) (Raboy 2009).

The lipid independent pathway starts with the phosphorylation of Ins to Ins(3)P_1_ catalyzed by Ins 3‐kinase (EC: 2.7.1.64). Ins(3)P_1_ is subsequently converted to Ins(3,4)P_2_ and to Ins(3,4,6)P_3_ by unknown kinases. Ins(3,4,6)P_3_ is phosphorylated to Ins(1,3,4,5,6)P_5_ by Ins 1,3,4‐triskisphosphate 5‐6‐kinase (EC: 2.7.1.134) which P_5_ is phosphorylated to PA by Ins pentakisphosphate 2‐kinase (EC 2.7.1.158) (Figure 11) (Raboy 2009; Silva et al. 2021).

### 12.2 Formation of PA in Plant‐Based Products

PA naturally occurs in a wide variety of plant‐based foods. For example, an average total PA content of 4.34 mg/g was found in 120 different plant‐based meat alternatives (Zhu et al. 2025). The PA content can also vary depending on food processing. In soft wheat, for instance, an average of 4 mg/g of PA was detected, whereas the milling product derived from soft wheat contained an average of 23 mg/g PA (García‐Estepa et al. 1999).

### 12.3 Degradation of PA

Degradation of PA is catalyzed by phytases (3.1.3.‐) and is based on sequential dephosphorylation of PA. Endogenous phytases are present in varying quantities depending on the crop species. For instance, legumes are low in phytase content (262–324 U/kg) compared to cereals, such as wheat (2886 U/kg) or barley (2323 U/kg) (Steiner et al. 2007). Plant phytases can be classified according to different criteria. One classification is based on the specific order in which phosphate groups are hydrolyzed from PA, resulting in three main classes: (i) inositol‐hexakisphosphate 3‐phosphohydrolases, or 3‐phytases (EC 3.1.3.8), (ii) inositol‐hexakisphosphate 5‐phosphohydrolases, or 5‐phytases (EC 3.1.3.72); and (iii) 4/6‐phytases (EC 3.1.3.26) (Figure 11). Phytases can be further classified according to their catalytic mechanism into (i) cysteine phosphatase, (ii) histidine acid phosphatase, (iii) purple acid phosphatases (PAP), and (iv) β‐propeller alkaline phytases (BPP) (Greiner et al. 2002). The hydrolysis pathways vary between species and enzymes, producing different Ins phosphate intermediates and final products. For example, in legume seeds, the degradation pathway begins with the hydrolysis of PA to D‐Ins(1,2,3,5,6)P_5_ by 4/6‐phytases (EC 3.1.3.26). Following the hydrolysis of D‐Ins(1,2,3,5,6)P_5_ to D‐Ins(1,2,3,6)P_4_ catalyzed by an unknown phytase. D‐Ins(1,2,3,6)P_5_ can be hydrolyzed into D‐Ins(1,2,6)P_3_ or D‐Ins(1,2,3)P_3_ catalyzed by unknown phytases. D‐Ins(1,2,6)P_3_ or D‐Ins(1,2,3)P_3_ are hydrolyzed into D‐Ins(1,2)P_2_ and D‐Ins(2,6)P_2_ by unknown phytases.Finally, D‐Ins(1,2)P_2_ and D‐Ins(2,6)P_2_ are converted to Ins(2)P by unknown phytases (Figure 11) (Greiner et al. 2002).

### 12.4 Degradation of PA in Plant‐Based Products

The degradation of PA varies depending on the food source and processing methods, which influence how much PA is broken down into less phosphorylated forms. Thermal processing can reduce PA content by up to 50% in legumes (Rehman and Shah 2005). Fermentation is another effective method for PA reduction. For example, fermentation with bacteria like *B. subtilis*, *L. plantarum*, *E. tasmaniensis*, and *E. gallinarum* decreased PA in rapeseed meal by nearly 60% (Hong et al. 2025). Yeasts, such as *S. cerevisiae*, also produce phytases and can further reduce PA when co‐cultured with bacteria, achieving a 79% reduction in wholemeal bread (Caputo et al. 2015). Filamentous fungi like *Aspergillus* spp., *Mucor* spp., and *Penicillium* spp. produce stable phytases across various temperatures and pHs (Joudaki et al. 2023). For example, *A. oryzae* reduced PA by 57% (Selle and Ravindran 2008). Another approach is to use purified (commercially available) phytases to degrade PA in a product. This method is widely used in feed to enhance its nutritional value (Haefner et al. 2005).

Overall, fermentation using LAB, yeasts, and fungi shows great potential to lower PA in plant‐based foods. However, the metabolic pathways behind PA degradation are only partially understood.

## 13 Oxalate

### 13.1 Biosynthesis of Oxalate

Oxalic acid is the simplest dicarboxylic acid and is found in a great number of photosynthetic organisms. Due to the high acidity of the compound (p*K*_a1_ = 1.25, p*K*_a2_ = 4.27), it is deprotonated at the physiological pH of most plant species, forming oxalate. In the environment of the plant cell, this anion is found in two configurations. In its soluble state, it is bound to sodium, ammonium, or potassium, whereas it becomes insoluble when bound to magnesium, calcium, or iron (Li et al. 2022). Oxalate is an anti‐nutritional factor (ANF), similar to PA, as its strong chelating nature reduces the bioavailability of various minerals, such as the aforementioned calcium, magnesium, and iron (Salgado et al. 2023). Additionally, the ingestion of excess oxalate can increase the risk of kidney stone formation, through the crystallization of calcium oxalate (Cai et al. 2018; Franceschi 2001).

Multiple pathways have been identified for the biosynthesis of oxalic acid in plants. Within these pathways, three compounds have been identified as direct precursors of oxalate. These are oxalyl‐l‐threonates, oxaloacetate, and glyoxylate (Cai et al. 2018; Li et al. 2022). The latter two are found in the glyoxylate cycle. Oxaloacetate is formed as a product of the oxidation of malate, facilitated by malate dehydrogenase (EC. 1.1.1.37). It is converted to oxalate enzymatically by oxalacetate acetyl hydrolase (EC 3.7.1.1). Glyoxylate is generated through the cleavage of isocitrate into succinate and glyoxylate by the enzyme isocitrate lyase (EC 4.1.3.1). Additionally, glyoxylate is formed through one of the by‐products of photorespiration, glycolate, which is converted to glyoxylate by glycolate oxidase (EC 1.1.3.15). This same enzyme is responsible for the conversion of glyoxylate to oxalate (EC 1.2.3.5) (Horner et al. 2000; Li et al. 2022).

**FIGURE 12:**
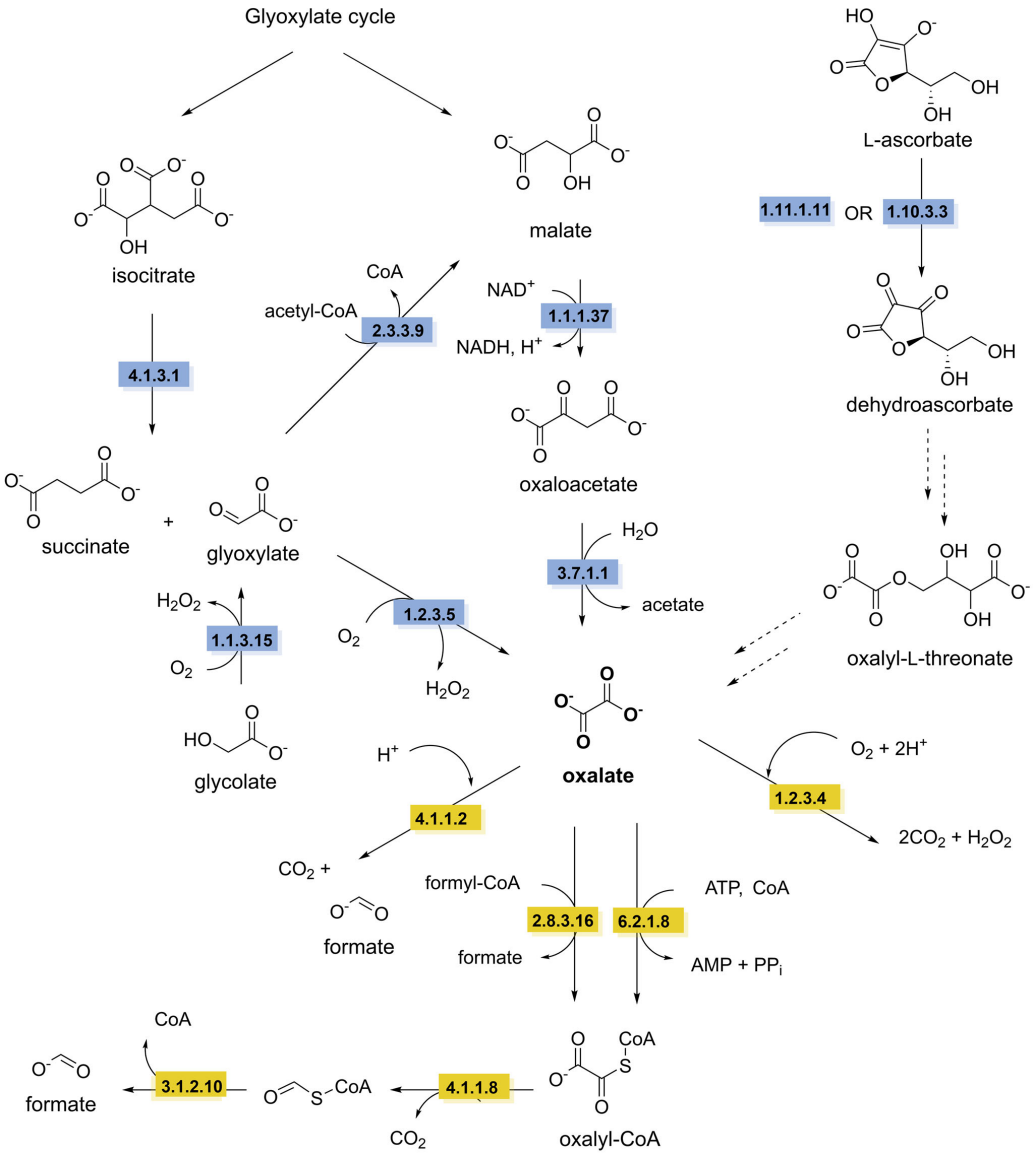
The synthesis and degradation pathways for oxalate. EC numbers are given for the known enzymes involved in synthesis (blue) and degradation (yellow). Dashed arrows indicate unknown steps. EC 1.1.1.37: malate dehydrogenase. EC 4.1.3.1: isocitrate lyase. EC 2.3.3.9: malate synthase. EC 3.7.1.1: oxaloacetate acetylhydrolase. EC 1.1.3.15: (*S*)‐2‐hydroxy‐acid oxidase. EC 1.2.3.5: glyoxylate oxidase. EC 1.11.1.11: l‐ascorbate peroxidase. EC 1.10.3.3: l‐ascorbate oxidase. EC 4.1.1.2: oxalate decarboxylase. EC 1.2.3.4: oxalate oxidase. EC 2.8.3.16: formyl‐CoA transferase. EC 6.2.1.8: oxalate‐CoA synthetase. EC 4.1.1.8: oxalyl‐CoA decarboxylase. EC 3.1.2.10: formyl‐CoA hydrolase.

The conversion of oxalyl‐l‐threonates to oxalate is considered one of the major sources of oxalate in plants (Horner et al. 2000; Khan et al. 2023). Oxalyl‐l‐threonates originate from the disproportionation and subsequent oxidation of l‐ascorbate (vitamin C). The conversion of l‐ascorbate to dehydroascorbate is catalyzed by either ascorbate oxidase (EC 1.10.3.3) or ascorbate peroxidase (EC 1.11.1.11). The remaining conversions can take place non‐enzymatically, but rate increases in vivo indicate that these reactions are also catalyzed by unknown enzymes (Green and Fry 2005; Truffault et al. 2017). The final step is the esterase‐mediated cleavage of oxalyl‐l‐threonates into oxalate and l‐threonates (Figure 12).

### 13.2 Formation of Oxalate in Plant‐Based Products

The highest oxalate content is reported for leafy greens such as spinach, parsley, and amaranth (Radek and Savage 2008; Salgado et al. 2023), but due to the high prevalence of oxalate synthesis in plants, oxalate can be found ubiquitously in vegetables, nuts, grains, and legumes. Therefore, plant‐based products, including meat and dairy alternatives, are often a source of oxalate.

### 13.3 Degradation of Oxalate

In plants, degradation of oxalate occurs primarily through one of two distinct pathways: oxidation of oxalate to carbon dioxide and hydrogen peroxide catalyzed by oxalate oxidase (EC 1.2.3.4) (Svedružić et al. 2005), and a multienzyme pathway resulting in the generation of formate and carbon dioxide. The latter starts with oxalate‐CoA synthetase (EC 6.2.1.8) which catalyzes the ATP‐dependent conversion of oxalate to oxalyl‐CoA (Foster et al. 2012; Foster and Nakata 2014). In turn, oxalyl‐CoA is converted to formyl‐CoA by oxalyl‐CoA decarboxylase (EC 4.1.1.8), which is subsequently broken down to formate and coenzyme A by formyl‐CoA hydrolase; however, genes encoding these enzymes have not been identified yet (EC 3.1.2.10) (Li et al. 2022; Lou et al. 2016). Although this pathway is predominantly found in plants, an oxalyl‐CoA synthetase discovered in *S. cerevisiae* suggests that it also plays a role in oxalate metabolism in yeast (Foster and Nakata 2014). An alternative enzyme able to catalyze the conversion of oxalate to oxalyl‐CoA is the ATP‐independent formyl‐CoA transferase (EC 2.8.3.16) (Berthold et al. 2008). It is mainly found in bacteria, a notable example of which is *Oxalobacter formigenes*, an oxalate‐degrading bacterium found in the human gut that uses oxalate as its primary carbon source (Daniel et al. 2021; Duncan et al. 2002). The next step in the pathway is the same, leading to the generation of formyl‐CoA. Then, the formyl‐CoA is used again by formyl‐CoA transferase to convert a second oxalate molecule into oxalyl‐CoA. Rather than either of these two pathways, oxalate‐degrading fungi, as well as several bacteria, utilize oxalate decarboxylase (EC 4.1.1.2) which directly converts oxalate into formate and carbon dioxide (Figure 12) (Zan et al. 2024).

### 13.4 Degradation of Oxalate in Plant‐Based Products

Fermentation is a promising strategy to generate plant‐based food products with a reduced oxalate content, as several fungal and bacterial species, including food‐grade species, such as *Bifidobacterium animalis* and *L. acidophilus*, are able to degrade oxalate (Grąz et al. 2023; Turroni et al. 2010; Xia et al. 2024).

## 14 Protease and Amylase Inhibitors

### 14.1 Biosynthesis of Protease and Amylase Inhibitors

Proteinaceous enzyme inhibitors include protease inhibitors (PIs) and AIs, which are nonvolatile organic compounds that inhibit proteases or amylases, respectively, by binding to the active site or inducing conformational changes that block substrate access (Farady  and Craik  2010; Lakshmana Senthil et al. 2015). PIs are widely distributed in plants, especially legumes, cereals, and tubers (Garcia‐Olmedo et al. 1987; Mandal et al. 2002; Ryan 1973), where PIs fulfill various biological functions, including defense against herbivores, protective responses to environmental stress, and roles as storage proteins (Bergey et al. 1996; Brzin and Kidrič 1996; Mosolov and Valueva 2005). AIs are also found in cereals and legumes, as well as in brown algae (*Ecklonia cava* and *Sargassum patens*) and microorganisms such as *Streptomyces* and *Actinomycetes* species, where one of their functions is to regulate α‐amylase activity (Franco et al. 2002; Lakshmana Senthil et al. 2015; Sokočević et al. 2011). Despite their beneficial functions in plants, both inhibitor types are considered ANFs in human and animal nutrition, as they reduce digestibility of proteins (PIs) or starch (AIs) (Christeller et al. 1998; Haq et al. 2004; Mareš et al. 1989; Mcewan et al. 2010; Nørgaard et al. 2019). PIs inhibit digestive proteases such as trypsin, chymotrypsin, and pepsin, lowering protein bioavailability, impairing nutrient absorption, and, in some cases, causing pancreatic hypertrophy (Liener 1994). Nevertheless, certain legume‐derived PIs have shown potential health benefits, including anticancer effects (Caccialupi et al. 2010; Magee et al. 2012; Salim et al. 2023). AIs, by inhibiting α‐amylases, delay starch digestion and glucose absorption, which may help moderate postprandial glucose levels and reduce caloric impact, making them of interest for managing type 2 diabetes and obesity (Kalinovskii et al. 2023; Kashtoh and Baek 2023; Kaur et al. 2021; Mahmood 2016; Sales et al. 2012).

PIs and AIs are both structurally diverse, occurring as proteins or molecules (Polya 2003; Svensson et al. 2004). Molecules, such as polyphenols, are addressed elsewhere in this review; here the focus is on proteinaceous enzyme inhibitors. PIs are found in multiple plant organs and cellular compartments (Kidrič et al. 2014; Mosolov and Valueva 2005), with more than 6700 plant members identified (Hellinger and Gruber 2019). Most belong to conserved superfamilies such as Kunitz‐type trypsin inhibitors, Bowman–Birk inhibitors, potato type I/II inhibitors, serpins, and cereal trypsin/α‐AIs (Brady 2003). Proteinaceous AIs are classified by tertiary fold into families including knottin‐like, lectin‐like (e.g., *Phaseolus* α‐AIs), cereal‐type (small albumins such as wheat amylase–trypsin inhibitors [ATIs]), Kunitz‐type (β‐trefoil fold), γ‐thionin‐like, thaumatin‐like, and microbial‐type inhibitors such as tendamistat (Geisslitz et al. 2021; Juge and Svensson 2006; Juhász et al. 2020; Rehm et al. 2009; Shewry and Casey 1999). Some, notably cereal‐type and Kunitz‐type inhibitors, are bifunctional and inhibit both α‐amylases and serine proteases (Barber et al. 1986; Di Maro et al. 2011; Drula et al. 2022). Across families of both PIs and AIs, conserved structural traits such as disulfide bridges, specific active‐site motifs, and stable three‐dimensional folds confer high resistance to heat, detergents, and proteolysis.

In plants, proteinaceous PIs and AIs are synthesized as pre(pro)proteins on the rough endoplasmic reticulum (ER), where they enter the secretory pathway. This synthesis involves the co‐translational removal of an N‐terminal signal peptide, followed by posttranslational modifications such as disulfide bond formation and, in some cases, *N*‐glycosylation. In PIs, these modifications promote correct folding, thermostability, and resistance to proteolysis in the gastrointestinal tract; for example, Bowman–Birk inhibitors are stabilized by seven disulfide bridges, conferring high resistance to heat, acidic conditions, and proteolytic enzymes (Gitlin‐Domagalska et al. 2020). It has been suggested that daily consumption of ∼100 g raw soybean or 200 g lentils could inhibit nearly all trypsin and chymotrypsin activity in the small intestine (Belitz and Weder 1990; Lajolo and Genovese 2002). In AIs, accumulation occurs predominantly in the endosperm and aleurone layers during seed development (Nielsen et al. 2004), and some, such as *Phaseolus vulgaris* α‐AI1, undergo proteolytic cleavage to yield α‐ and β‐subunits (Maczó et al. 2015; Pueyo et al. 1993). As with PIs, these structural features contribute to stability and protease resistance (Svensson et al. 2004), and extensive structural studies have clarified the folding pathways and inhibitory mechanisms of various AI classes (Shewry and Casey 1999; Svensson et al. 2004).

### 14.2 Formation of Protease and Amylase Inhibitors in Plant‐Based Products

PIs and AIs are produced in various plant tissues but accumulate most prominently in seeds, where they protect storage proteins (PIs) or starch reserves (AIs) and contribute to defense against pests and pathogens (Giri and Kachole 1998; Juge and Svensson 2006; Mosolov and Valueva 2005; Ryan 1973; Shewry and Casey 1999; Sivakumar et al. 2006; Svensson et al. 2004; Weselake et al. 1983). In legumes, PIs can comprise up to 10% (w/w) of total seed protein (Srikanth and Chen 2016), whereas in cereals ATIs constitute ∼2%–4% (w/w) of grain protein, and in *P. vulgaris*, α‐AI1 can represent 9%–11% (w/w) (Geisslitz et al. 2021; Moreno and Chrispeels 1989). Rich PI sources include beans, potatoes, barley, squash, millet, wheat, buckwheat, groundnut, chickpea, pigeon pea, corn, and pineapple (Salim et al. 2023), with legumes such as soybean, chickpea, and lupin particularly important for food applications (Clemente 2014). Two PI families, Bowman–Birk and Kunitz‐type inhibitors, dominate in these crops (Salim et al. 2023) and account for the high PI content in soy protein isolates and flours (Dipietro and Liener 1989). AIs occur widely in cereals including wheat, barley, oats, rye, rice, corn, sorghum, finger millet, and barnyard millet (Altenbach et al. 2011; Chen et al. 1992; Feng et al. 1991; Gadge et al. 2015; Garcia‐Olmedo et al. 1987; Maskos et al. 1996; Panwar et al. 2018; Sagu et al. 2020; Wang et al. 2014). In seeds, AIs can also act as reserve proteins (Yamada et al. 2001). PI biosynthesis is dynamically regulated, with several Bowman–Birk inhibitor isoforms showing peak expression during intermediate stages and declining as seeds mature, supporting their role in protecting developing storage proteins from premature degradation and contributing to seed defense (Vorster et al. 2023). In AIs, expression is closely linked to developmental stages such as grain filling (Nielsen et al. 2004). Abiotic and biotic stresses such as UV exposure, wounding, or herbivory can further induce PI biosynthesis via jasmonic acid signaling within the octadecanoid pathway (Koiwa et al. 1997).

### 14.3 Degradation Protease and Amylase Inhibitors

Both PIs and AIs can undergo enzymatic degradation in plants and by microbes. In plants, seed germination activates endogenous proteases and amylases that mobilize storage reserves and reduce inhibitor levels (Clemente 2014; Savelkoul et al. 1992). In legumes, PI activity can decline by 18%–64% within days, depending on species and duration. For example, a 64% reduction after 48 h in fava bean (Sharma and Sehgal 1992), 18% after 6 days and 45% after 10 days in lentils (Frias et al. 1995), significant decreases only after 10 days in kidney beans (Nielsen and Liener 1988; Pusztai 1972), and 19% after 8 days in cowpea (Kalpanadevi and Mohan 2013). For AIs, reported reductions include 67.1% after 5 days in Great Northern beans (Sathe et al. 1983), 40% after 7 days in cranberry beans (Kotaru 1987), and gradual decline over 15 days in pigeon pea (Ambekar et al. 1996). In cereals, α‐AIs disappear from aleurone and endosperm tissues during early imbibition, coinciding with α‐amylase secretion (Kanzaki et al. 1993). Although germination rarely eliminates PIs or AIs entirely, it improves protein or starch digestibility and amino acid availability while meeting the metabolic needs of the growing seedling. Microbial degradation also plays a role in PI and AI degradation. For instance, rumen microbes fermenting soybean meal in vitro were found to inactivate and degrade trypsin inhibitor proteins over time, with a rapid loss of inhibitory activity followed by slower proteolytic breakdown (Hoffmann et al. 2003). Similarly, microbial fermentation of legumes and cereals reduces AI and ATI bioactivity, as shown with specific *Lactobacillus* strains during wheat fermentation (Caminero et al. 2019).

### 14.4 Degradation of Protease and Amylase Inhibitors in Plant‐Based Products

PIs and AIs are prevalent in plant‐based products due to their abundance in legumes, cereals, and tubers and can be reduced by physical, chemical, and biological processing methods (Avilés‐Gaxiola et al. 2018; Juge and Svensson 2006; Mosolov and Valueva 2005; Ryan 1973; Samtiya et al. 2020; Svensson et al. 2004). Physical approaches such as cooking, extrusion, roasting, autoclaving, milling, and baking can partially inactivate inhibitors, although heat‐stable types such as the Kunitz trypsin inhibitor and Bowman–Birk inhibitor (PIs) and many ATIs (AIs) resist conventional thermal processing due to their disulfide bridge‐stabilized structures (Aoki‐Shioi et al. 2023; Liu 1997; Samtiya et al. 2020; van der Ven et al. 2005). Soaking removes some soluble antinutritional factors but has limited effect on PIs (Avilés‐Gaxiola et al. 2018). Chemical treatments, including acids, bases, reducing agents, or additives such as sodium metabisulfite for soybean PIs and l‐cysteine for chickpea PIs, can further reduce activity (Avilés‐Gaxiola et al. 2018). However, because disulfide‐rich inhibitors are highly resistant, biological strategies such as germination and fermentation often yield the most significant reductions.

Fermentation is particularly effective for both PIs and AIs and often improves nutritional quality in parallel. In legumes, solid‐state fermentation with *A. oryzae* (alone or with *Lactobacillus brevis*) can reduce trypsin inhibitor content in soybean meal by up to 89% under optimized conditions (Gao et al. 2013), and submerged fermentation with *B. licheniformis* lowers activity by ∼75% through secretion of serine proteases (Phengnuam and Suntornsuk 2013). LAB fermentation, including with *L. plantarum*, significantly decreases trypsin inhibitor levels in pulses and cereals, likely via extracellular protease activity (Coda et al. 2015). Fermentation also improves protein digestibility and reduces allergenicity. For instance, fungal fermentation of unfermented soybean meal increased in vitro protein digestibility (IVPD) from 61% to 67%, whereas bacterial fermentation raised it to 76%, with concurrent reductions in allergenic potential and increases in essential amino acids (Hong et al. 2004; Mukherjee et al. 2015; Pi et al. 2019; Song et al. 2008). *B. subtilis* fermentation enhanced in vitro digestibility 1.5‐fold while reducing trypsin inhibitor activity from 27.3 to 2.1 TIU/g (trypsin inhibitor units per gram) (Ketnawa and Ogawa 2019; Ojokoh and Yimin 2011). Combining germination with additional treatments may further improve outcomes. In *Vigna unguiculata*, a 96‐h germination followed by autoclaving resulted in complete degradation of several antinutritional factors and improved protein digestibility (Kalpanadevi and Mohan 2013). For AIs, sourdough fermentation with selected LAB activates microbial aspartic proteases under low‐pH conditions, cleaving ATI polypeptides and reducing inhibitory activity, whereas yeast fermentation leaves ATIs intact (Huang et al. 2020). In cereals such as pearl millet, traditional fermentations (e.g., lahoh bread) reduce AI activity by ∼50.8% in 24 h (Anastasio et al. 2010). These findings highlight microbial fermentation as a promising method to reduce the anti‐nutritional effects of PIs and AIs in plant‐based products.

## 15 Lectins

### 15.1 Biosynthesis of Lectins

Lectins are glycoproteins with the ability to bind carbohydrates or glycoconjugates (Padiyappa et al. 2025; Popova and Mihaylova 2019). In literature they are commonly referred to as agglutinins or hemagglutinins, as they can agglutinate different cell types, including erythrocytes, leukocytes, tumor cells, and bacteria (Padiyappa et al. 2025). Lectins are considered one of the most common antinutritional factors present in plant‐based foods. Although some lectins are more susceptible to digestive enzymes, it has been reported that 90% of orally administered lectins pass through the digestive tract largely intact (Lucius 2020; Peumans and Van Damme 1996). Certain lectins are toxic, as they can cause intestinal damage and impair nutrient absorption by binding to the surface of intestinal epithelial cells. This intestinal damage may allow bacteria from the gastrointestinal tract to enter the bloodstream, bypass the immune system, and circulate throughout the body (Mukherjee et al. 2015; Popova and Mihaylova 2019; Samtiya et al. 2020). To reduce the toxicity cause by lectins, either lectins or their activity should be eliminated or reduced. Although lectins in plant‐based foods are considered antinutritional factors, numerous studies have also highlighted their potential therapeutic properties, including antiangiogenic, antitumor, and antidiabetic activities. These effects depend on the quantity of lectins present and their source (Adamcová et al. 2021; Konozy and Osman 2024; López‐Moreno et al. 2022; Popova and Mihaylova 2019; Sharon 2004).

There are more than 500 different plant lectins that were isolated and characterized, and the majority have been classified into seven families of structurally and evolutionary related proteins. Generally, lectin families are fairly homogenous, both in how they are synthetized and in their tertiary structure, although some differences between the families remain (Peumans et al. 2001). Most plant lectins are synthesized on the ER as pre(pro)proteins that undergo different co‐ and posttranslational modifications, depending on the type of lectin. These modifications often include the removal of an N‐ or C‐terminal signal peptide, followed by *N*‐glycosylation or proteolytic cleavage at a single site, but can be rather specific for individual lectins. Legume lectins are known to form a rigid and strong structure that makes them fairly resistant towards many proteolytic enzymes (Peumans et al. 2001; van Damme et al. 2007).

### 15.2 Formation of Lectins in Plant‐Based Products

Lectins are present in most plant compartments, but their concentration varies, with seeds often containing the highest concentrations of lectins (Lucius 2020). They can be found in variety of plant species, such as wheat, beans, peas, quinoa, potatoes, and nuts (Popova and Mihaylova 2019). The complete biological role of lectins in plants remains unknown, although they are believed to play a role in defense against viral, bacterial, and fungal pathogens, as well as predators, such as insects (Konozy and Osman 2024; Lucius 2020).

### 15.3 Degradation of Lectins

Heat treatment is among the most applied methods used for reducing lectin content in plant‐derived substrates (Cuadrado et al. 2002; López‐Moreno et al. 2022; Padiyappa et al. 2025; Popova and Mihaylova 2019; Samtiya et al. 2020). One of the primary challenges with thermal processing is its high cost, along with the potential degradation of valuable nutrients in the final product (López‐Moreno et al. 2022). Generally, lectins seem to be resistant towards digestive proteolytic enzymes, due to their rigid tertiary structure, which causes their toxicity upon oral administration (Padiyappa et al. 2025; Peumans et al. 2001). In one study it was suggested that a short heat treatment could make lectins more prone to proteolytic degradation due to disruption of the tertiary structure of the protein (Rhodes and Milton 1997). Although proteolytic digestion may not result in complete inactivation of hemagglutinin molecules, it was suggested that the lectins may be less harmful to the intestinal wall (Ojimelukew et al. 1995). Another strategy to reduce lectin activity involves administering complementary carbohydrates, such as simple sugars or oligosaccharides, which lectins can bind to, thereby inactivating them (Freed 1999; Lucius 2020).

### 15.4 Degradation of Lectins in Plant‐Based Products

Fermentation has shown some promise in reducing the content of lectins in plant‐based products. For example, fermentation with LAB of lentil meal completely removed lectin content without the need for heat treatment, with the majority of the strains belonging to *Lactobacillus* and *Pediococcuis* genera (Cuadrado et al. 2002). Another study showed a 95% reduction of lectins after fermentation with different genera and species of fungi and bacteria, although some lectin activity was still observed in samples that were not treated with heat after the fermentation. It is assumed that hydrolysis is the primary mechanism responsible for lectin inactivation during fermentation. It has also been reported that a strain of *Leuconostoc mesenteroides* is capable of hydrolysing lectins in soybeans, navy beans, black beans, and other legumes. The enzymes secreted by this strain, that participate in lectin hydrolysis, consist of a mixture of β‐*N*‐acetylglucosaminidase, α‐d‐mannosidase, and an unspecified protease, though none of the mentioned enzymes have been well characterized (Reddy and Pierson 1994). Nonetheless, the underlying bioconversions and exact pathways involved in lectin degradation in plant‐based products throughout fermentation are not characterized yet.

## 16 Occurrence of Known Enzymes and Pathways

**FIGURE 13:**
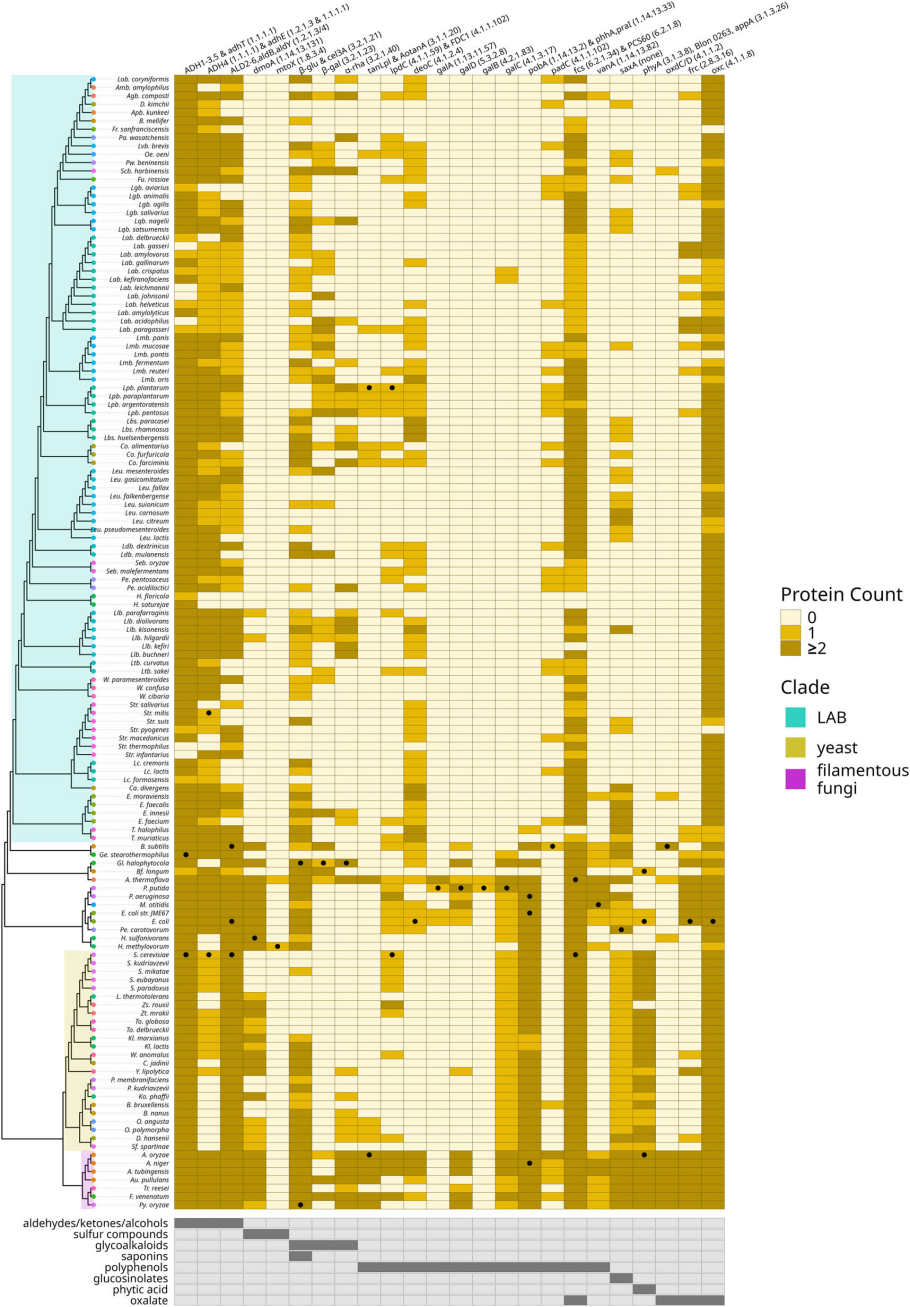
Heatmap of functional groups for known enzymes involved in the biodegradation of the investigated compounds across the proteomes of relevant organisms. To the left is a species tree of the query organisms that were searched for enzymes involved in the degradation of molecules relevant to plant‐based food fermentations including lactic acid bacteria (blue), yeast (yellow), and filamentous fungi (pink). For each species, its reference proteome was downloaded from NCBI‐genomes^1^ (Table S2). The heatmap on the right was generated using OrthoFinder^2,3^ and it shows the orthologous groups for identified enzymes. Each column represents one functional group, and each row corresponds to a species. The heatmap indicates the absence (0) or presence (1: one copy and ≥2: multiple copies) of a protein in a species belonging to the functional group of a target enzyme. Black dots indicate the query organisms used (Table S2). A more detailed description of how this figure was generated can be found in Section S2. The raw data used to generate the figure can be found in Table S3.

This review summarizes the enzymatic reactions known to be involved in the conversion of off‐flavors and antinutrients in plant‐based substrates. All the synthesis and degradation enzymes described in this review are listed in Table S1. To check whether these enzymes are present in microorganisms associated with food fermentation, a bioinformatic analysis was performed to identify orthologous enzymes. The presence of such enzymes could suggest that these reactions may take place during the fermentation of plant‐based substrates (Figure 13).

Figure 13 shows that many of the enzymes identified in this review are absent from microorganisms relevant for food fermentation. This does not necessarily exclude the existence of other convergently evolving enzymes with the same catalytic function. However, the enzymes ADH (EC 1.1.1.1), ALDH (EC 1.2.1.4), β‐glucosidase (EC 3.2.21), aldolase (EC 4.1.2.4), feruloyl‐CoA synthase (EC 6.2.1.34), oxalate‐CoA synthetase (EC 6.2.1.8), and oxalyl‐CoA decarboxylase (EC 4.1.1.8) are relatively highly present in bacteria and yeast. The high abundance of aldolase may not be particularly interesting, as this enzyme functions only at the final step of the gallic acid degradation pathway. If other essential enzymes are absent, degradation is unlikely to happen (Figure 8). The high abundance of ADH and ALDH is not surprising since almost all LABs and yeast contain a copy of these enzymes and they form a well known example of how the knowledge on enzymatic conversions of off‐flavors has been widely applied to improve the quality of plant‐based substrates.

The enzymes feruloyl‐CoA synthase (EC 6.2.1.34) and oxalate‐CoA synthetase (EC 6.2.1.8) are in the same orthologous group and catalyze both the addition of coenzyme A in the degradation of polyphenols and oxalate, respectively. Interestingly, research showed the conversion of ferulic acid into vanillin, with feruloyl‐CoA synthase catalyzing the first step (Mostafa and Hashem 2023). Oxalate–CoA synthetase catalyzes one of the initial steps in oxalate detoxification, a pathway that has been well characterized in *S. cerevisiae* (Foster and Nakata 2014). The second step in this pathway is catalyzed by oxalyl‐CoA decarboxylase seems to be also highly abundant in LABs and yeast. These results suggest that oxalate‐degrading enzymes may be present in a broader range of organisms. This hypothesis is in line with a study where oxalate‐degrading activity among LABs strains was studies. A screening of 79 LAB strains isolated from food sources showed that 31 of these stains were able to degrade oxalate (Murru et al. 2017). Another screening also identified more 17 LABs to degrade oxalate (Gomathi et al. 2014). β‐Glucosidases are also commonly found in LABs and yeast, which is promising for the cleavage of glycosidic bonds in saponins and GAs. However, experimental validation of the substrate specificity of these enzymes is necessary to confirm this reaction.

In yeast, additional enzymes like DMS monooxygenase (EC 1.14.13.131), CHA aldolase (EC 4.1.3.17), 4‐hydroxybenzoate 3‐hydroxylase (EC 1.14.13.2, 1.14.13.33), ITC hydrolase (SaxA), 3‐phytases (EC 3.1.3.8), and 4/6‐phytases (EC 3.1.3.26) were found to be present in a large range of genera. DMS monooxygenase catalyzes the conversion of DMS to methanethiol. Although most of the selected yeasts possess a copy of this enzyme in their genome, experimental evidence for this conversion in yeast is lacking. In contrast, a study reports the formation of DMDS in *Kluyveromyces lactis* (Lu et al. 2018). On the other hand, enzymes such as phytases are widely distributed and expressed among different yeasts (Capusoni et al. 2021). Isothiocyanate hydrolases have not been experimentally characterized in yeast, but they represent an interesting target for the degradation of bitter ITC compounds found in seed‐derived products by food‐grade microorganisms.

Although certain enzymes are widespread among LABs and yeasts, the presence of many others is limited (Figure 13).

## 17 Conclusion

Understanding the synthesis and degradation pathways of off‐flavors and antinutrients in plant‐based substrates is important to improve the quality of these products. We found that only few molecule classes, aldehydes, alcohols, acids, and oxalate, have well‐characterized degradation pathways in food‐grade organisms. For other classes like sulfur compounds (DMS and DMDS) and ITCs derived from GSLs the pathways are well described, however not in food‐grade organisms. To degrade these molecules in plant‐based substrates through fermentation, it is of interest to investigate if these or similar enzymes can be identified in food‐grade organisms or if other degradation pathways in food‐grade organisms exist. Other research targets of interest are the compound classes where the degradation or conversion pathways are completely unknown, like furans, alkyl‐methoxypyrazines, and sulfur compounds (DMTS). PA, saponins, pyrimidine glycosides, and polyphenols have only partially described degradation pathways. For example, deglycosylation of GSLs, saponins, and pyrimidine glycosides is well understood; however, the further degradation of their aglycones remains unknown. The sensory properties of these intermediate products are often also unknown; therefore, sensory analysis or degradation of these aglycones is interesting for future research. The challenge with lectins, amylase, and PIs lies in making these proteins more susceptible to microbial proteolytic activity. Although boiling can achieve this, a method that preserves protein functionality is unknown. Many studies show the degradation or conversion of off‐flavors or antinutrients with unknown pathways, this suggests that many (enzymatic) reactions possibly responsible for the observed reduction of off‐flavors and antinutrients during fermentation remain unknown. Experimental studies are therefore essential to characterize enzymes or mechanisms that drive these conversions. As shown for aldehyde conversion, a better understanding of such pathways can improve the flavor quality of plant‐based substrates.

## 18 Outlook

To address this knowledge gap in future research, the application of advanced bioinformatics tools focused on enzyme function (biotransformation) prediction is crucial. Approaches based on evolutionary principles (Altschul et al. 1990), structure‐based function inference such as Alphafold (Abramson et al. 2024), comparative genome analysis with context‐aware algorithms (Jha et al. 2025), and machine learning‐guided annotation, such as contrastive learning (Sanderson et al. 2023; Yu et al. 2023), have shown promise in predicting enzymatic activities. These computational approaches allow for the prioritization of candidate enzymes for experimental validation and offer insights into potentially novel biotransformation. Efforts such as InterPro build on this by integrating various computational approaches to accurately classify enzyme families (Blum et al. 2025). Once metabolic pathways have been reconstructed through predicted and experimentally validated enzyme activities, computational analyses, such as those assessing the presence and distribution of pathway‐encoding genes across species, can be applied to guide the selection of species and strains for targeted fermentation applications for removing antinutrients or off‐flavors, or to produce desired flavors. It should, however, be noted that even though these computational approaches show clear promise, their predictive power has limitations particularly for poorly studied enzyme families involved in secondary metabolism, hence antinutrient and off‐flavor utilization and flavor biosynthesis. Therefore, functional characterization through (medium‐ to high‐throughput) biochemical assays remains crucial for not only confirming (predicted) enzyme activities but also uncovering novel catalytic functions (Finnigan et al. 2021; Kuznetsova et al. 2006). Assay‐based validation enables the discovery of unknown enzyme promiscuities, substrate specificities, and reaction mechanisms that may not be predictable computationally. In addition analytical techniques to identify volatile and nonvolatile compounds need to be improved and links between flavor active compounds and their sensory perception established. This counts especially for molecules with low flavor thresholds. The importance of generating empirical data not only drives discoveries but also allows improvements to computational approaches, especially those driven by AI technology. Taken together, future efforts focused on the development of strategies that integrate state‐of‐the‐art computational approaches with systematic high‐throughput enzyme assays offer a synergistic framework needed to advance the discovery of enzyme function and the reconstruction of secondary metabolism of flavor metabolism that could guide novel food fermentation strategies.

## Author Contributions

**Robin I. Kuijpers**: conceptualization, writing – original draft, methodology, visualization, writing – review and editing. **Isabel O. de Moya Clark**: writing – original draft, visualization, methodology. **Tomás Cavaco**: writing – original draft. **Vivian Nemanič**: writing – original draft. **Beatrice Tagliabue**: writing – original draft. **Ainhoa Valero Abad**: writing – original draft. **Wiebe M. Wennekers**: writing – original draft. **Mengqiu Zhang**: writing – original draft. **Koen Van Zwet**: writing – original draft. **Sanne Abeln**: methodology, visualization, writing – review and editing. **Sofia Moco**: writing – review and editing, supervision, funding acquisition. **Caroline E. Paul**: writing – review and editing, visualization, supervision, funding acquisition. **Halima Mouhib**: writing – review and editing, supervision, visualization, funding acquisition, methodology. **Richard A. Notebaart**: writing – review and editing, supervision, funding acquisition. **Eddy J. Smid**: writing – review and editing, supervision, funding acquisition. **Bas Teusink**: writing – review and editing, supervision, funding acquisition. **Herwig Bachmann**: writing – review and editing, supervision, conceptualization, funding acquisition, project administration, methodology.

## Conflicts of Interest

H.B. is part time employed by NIZO Food Research. H.B. is part time employed by NIZO Food Research. The other authors declare no conflicts of interest.

## Supporting information




**Supplementary Materials**: crf370449‐sup‐0001‐SuppMat.docx

**Supplementary Table S2**: crf370449‐sup‐0002‐TableS2.xlsx

**Supplementary Table S3**: crf370449‐sup‐0003‐TableS3.xls
