# Supplementary material for: Gap Analysis of Metabolic Conversions of Off‐Flavors and Antinutrients in Plant‐Based Substrates
Source: Compr Rev Food Sci Food Saf. 2026 Mar 31;25(3):e70449. doi: 10.1111/1541-4337.70449 (PMC13039779; doi:10.1111/1541-4337.70449)
Supplement: Supplementary file 1 — Supplementary Materials: crf370449‐sup‐0001‐SuppMat.docx [file CRF3-25-e70449-s003.docx]

## Supplementary materials

### Gap Analysis of Metabolic Conversions of Off-flavors and Antinutrients in Plant-Based Substrates

Robin I. Kuijpers^a^, Isabel O. de Moya Clark^b^, Tomás Cavaco^a^, Vivian Nemanič^c^, Beatrice Tagliabue^d^, Ainhoa Valero Abad^c^, Wiebe M. Wennekers^c^, Mengqiu Zhang^e^, Koen van Zwet^c^, Sanne Abeln^f^, Sofia Moco^e^, Caroline E. Paul^d^, Halima Mouhib^b^, Richard A. Notebaart^c^, Eddy J. Smid^c^, Bas Teusink^a^, Herwig Bachmann^a, g,^*.

^a^ Systems Biology Lab, A-LIFE, AIMMS, Vrije Universiteit Amsterdam, Amsterdam, The Netherlands
^b^ Department of Computer Science, VU Bioinformatics Group, Vrije Universiteit Amsterdam, De Boelelaan 1105, 1081 HV Amsterdam, The Netherlands.
^c^ Food Microbiology, Wageningen University & Research, The Netherlands
^d^ Biocatalysis section, Department of Biotechnology, Delft University of Technology, Delft, The Netherlands.
^e^ Chemistry and Pharmaceutical Sciences, AIMMS, Vrije Universiteit Amsterdam, Amsterdam, The Netherlands
^f^ Department of Computer Science, AI Technology for Life, Universiteit Utrecht, Heidelberglaan 8, 3584 CS Utrecht, The Netherlands
^g^ NIZO Food Research, Ede, The Netherlands

***Contact information for Corresponding author**  
Systems Biology Lab, A-LIFE, AIMMS, Vrije Universiteit Amsterdam, Amsterdam, The Netherlands 
h.bachmann@vu.nl

### S1 Enzyme table

Table S1: Enzymes involved in synthesis (**O**) and degradation (**O**) of off-flavor compounds and antinutrients in plant-based substrates.

| **Compound class** | **EC number** | **Gene name** | **Protein name** | **Uniprot ID** | **RHEA** | **Genome accession** | **Protein accession** | **Reference** |
| --- | --- | --- | --- | --- | --- | --- | --- | --- |
| **Aldehydes/ketones/alcohols** | **O** 1.13.11.12 | *LOX2* | Linolenate hydroperoxide lyase | P38418 | 22780 | - | - | (Bannenberg et al., 2009) |
|  | **O** 1.13.11.58 | *LOX5* | Linoleate 9S-lipoxygenase 5 | Q9LUW0 | 30291 | - | - | (Bannenberg et al., 2009) |
|  | **O** 4.2.99.- | *CYP74B2 (HPL)* | Linolenate hydroperoxide lyase | Q9ZSY9 | - | - | - | (Duan et al., 2005) |
|  | **O O** 1.1.1.1 | *ADH1* | Alcohol dehydrogenase 1 | P00330 | 10736 | GCF_000146045.2 | NP_014555.1 | (Bennetzen & Hall, 1982) |
|  | **O O** 1.2.1.4 | *ALD5* | Aldehyde dehydrogenase 5 | P40047 | 25298 | GCF_000146045.2 | NP_010996.2 | (X. Wang et al., 1998) |
| **Sulfur compounds** | **O** 4.4.1.11 | *mdeA* | L-methionine gamma-lyase | P13254 | 23800 | - | - | (Inoue et al., 1995) |
|  | **O** 4.4.1.13 | *metC* | Cystathionine beta-lyase | A2RM21 | 23800/24931 | - | - | (Dobric et al., 2000) |
|  | **O** 4.4.1.1 | *cys3* | Cystathionine gamma-lyase | P31373 | 23800/24931 | - | - | (Ono et al., 1992) |
|  | **O** 2.6.1.57 | *tyrB* | Aromatic-amino-acid aminotransferase | P04693 | 17533 | - | - | (Fotheringham et al., 1986) |
|  | **O** 2.6.1.42 | *ilvE* | Branched-chain-amino-acid aminotransferase | P0AB80 | 17533 | - | - | (Kuramitsu et al., 1985) |
|  | **O** 2.1.1.334 | *mddA* | Methanethiol S-methyltransferase | A0A0F6P9C0 | 50428 | - | - | (Carrión et al., 2015) |
|  | **O** 4.4.1.28 | *MJ1025* | L-cysteine desulfidase | Q58431 | 24931 | - | - | (Tchong et al., 2005) |
|  | **O** 2.6.1.1 | *aspC* | Aspartate aminotransferase | P00509 | 17444 | - | - | (Kondo et al., 1984) |
|  | **O** 2.6.1.3 | *-* | - | - | 17444 | - | - | (Ubuka et al., 1992) |
|  | **O** 2.8.1.2 | sseA | 3-mercaptopyruvate sulfurtransferase | P31142 | 21740 | - | - | (Colnaghi et al., 2001) |
|  | **O O** 1.8.1.21 | - | dimethyldisulfide reductase | - | - | - | - | (De Bont et al., 1981) |
|  | **O O** 1.8.3.4 | *mtoX* | Methanethiol oxidase | A0A291P0C1 | 11812 | GCF_013626205.1 | WP_181338307.1 | (Eyice et al., 2018)) |
|  | **O O** 1.14.13.131 | *dmoA* | Dimethyl-sulfide monooxygenase | E9JFX9 | 31355 | GCF_013306565.1 | WP_173954302.1 | (Boden et al., 2011) |
| **Pyrazines** | **O** 2.1.1.68 | *OMT2* | O-methyltransferase | D9YKY5 | - | - | - | (Dunlevy et al., 2010) |
| **Glycoalkaloids** | **O** 3.2.1.21 | *β-glu* | beta-glucosidase | A0AA94XZP8 | - | GCF_024662055.1 | WP_131090259.1 | (W. Wang et al., 2022) |
|  | **O** 3.2.1.23 | *β-gal* | Beta-galactosidase | A0AA95BQ52 | - | GCF_024662055.1 | WP_257745710.1 | (W. Wang et al., 2022) |
|  | **O** 3.2.1.40 | *α-rha* | alpha-L-rhamnosidase | A0AA95BR09 | - | GCF_024662055.1 | WP_257745621.1 | (W. Wang et al., 2022) |
| **Glycosides** | **O** 3.2.1.21 | *β-glu* | beta-glucosidase | A0AAV0ZI92 | - | - | - | (Pulkkinen et al., 2016) |
| **Saponins** | **O** 5.4.99.39 | *BAS* | β-amyrin synthase | B6EXY6 | 31007 | - | - | (Shibuya et al., 2009) |
|  | **O** 1.14.14.134 | *CYP93E1* | Beta-amyrin 24-hydroxylase | Q9XHC6 | 30991 | - | - | (Shibuya et al., 2006) |
|  | **O** 3.2.1.188 | *P60A* | Avenacosidase 1 | Q38786 | 38911 | - | - | (Gus-Mayer et al., 1994) |
|  | **O** 3.2.1.21 | *cel3A* | Beta-glucosidase cel3A | G4NI45 | - | GCF_000002495.2 | XP_003720272.1 | (Takahashi et al., 2017) |
| **Polyphenols** | **O** 2.5.1.54 | *aroG* | 3-deoxy-7-phosphoheptulonate synthase | P0AB91 | 14717 | - | - | (Davies & Davidson, 1982) |
|  | **O** 4.2.3.4 | *aroB* | 3-dehydroquinate synthase | P07639 | 21968 | - | - | (Millar & Coggins, 1986) |
|  | **O** 4.2.1.10 | *aroD* | 3-dehydroquinate dehydratase | P05194 | 21096 | - | - | (Duncan et al., 1986) |
|  | **O** 1.1.1.25 | *aroE* | shikimate dehydrogenase (NADP+) | P15770 | 17737 | - | - | (Davies & Davidson, 1982) |
|  | **O** 2.4.1.136 | *UGT84A13* | gallate 1-β-glucosyltransferase | V5LLZ9 | 15249 | - | - | (Mittasch et al., 2014) |
|  | **O** 4.3.1.24 | *AvPAL* | phenylalanine ammonia-lyase | Q3M5Z3 | 21384 | - | - | (L. Wang et al., 2008) |
|  | **O** 1.14.14.91 | *CYP73A5* | trans-cinnamate 4-monooxygenase | P92994 | 10608 | - | - | (Lee et al., 1997) |
|  | **O** 6.2.1.12 | *4CL1* | 4-coumarate—CoA ligase | Q42524 | 19641 | - | - | (Bell-Lelong et al., 1997) |
|  | **O** 2.1.1.68 | *COMT1* | caffeate O-methyltransferase | P28002 | 20225 | - | - | (Gowri et al., 1991) |
|  | **O** 2.3.1.74 | *CHS* | chalcone synthase | P13114 | 11128 | - | - | (Liou et al., 2018) |
|  | **O** 5.5.1.6 | *CHI* | chalcone isomerase | P41088 | 79891 | - | - | (Jez et al., 2000) |
|  | **O** 3.1.1.20 | *tanLpl* | Tannase | B3Y018 | 16365 | GCF_009913655.1 | WP_087614957.1 | (Ren et al., 2013) |
|  | **O** 4.1.1.59 | *lpdC* | Gallate decarboxylase | F9US27 | 12749 | GCF_009913655.1 | WP_087614958.1 | (Jiménez et al., 2013) |
|  | **O** 1.97.1.2 | *athL* | Pytogallol hydroxytransferase | P80563 | - | - | - |  |
|  | **O** 1.3.1.57 | *phgR* | Phloroglucinol reductase | P57793 | 10080 | GCF_900104415.1 | WP_090173699.1 | (Haddock & Ferry$, 1989) |
|  | **O** 4.1.2.4 | *deoC* | Deoxyribose-phosphate aldolase | P0A6L0 | - | - | - |  |
|  | **O** 1.13.11.57 | *galA* | Gallate dioxygenase | Q88JX5 | 28927 | GCF_045571375.1 | WP_010953452.1 | (Nogales et al., 2011) |
|  | **O** 5.3.2.8 | *galD* | 4-oxalomesaconate tautomerase | Q88JY0 | 28931 | GCF_045571375.1 | WP_010953447.1 | (Nogales et al., 2011) |
|  | **O** 4.2.1.83 | *galB* | 4-oxalmesaconate hydratase | Q88JX8 | 17401 | GCF_045571375.1 | WP_010953449.1 | (Nogales et al., 2011) |
|  | **O** 4.1.3.17 | *galC* | 4-carboxy-4-hydroxy-2-oxoadipic acid aldolase | Q88JX9 | 28935 | GCF_045571375.1 | WP_010953448.1 | (Nogales et al., 2011) |
|  | **O** 1.14.13.2 | *pobA* | p-hydroxybenzoate hydroxylase | P20586 | 19477 | GCF_038397815.1 | WP_341987052.1 | (Entsch et al., 1988) |
|  | **O** 4.1.1.102 | *FDC1* | Ferulic acid decarboxylase 1 | Q03034 | 33227 | GCF_000146045.2 | NP_010828.1 | (Mukai et al., 2010) |
|  | **O** 6.2.1.34 | fcs | Feruloyl-CoA synthase | Q9EY88 | 36251 | GCF_003752125.1 | WP_123685744.1 | (Achterholt et al., 2000) |
|  | **O** 1.14.13.82 | *vanA* | Vanillate O-demethylase oxygenase subunit | P12609 | 13021 | GCF_039718995.1 | WP_346840306.1 | (Brunel & Davison, 1988) |
| **Glucosinolates** | **O** 2.6.1.88 | *BCAT4* | Methionine aminotransferase | Q9LE06 | 31763 | - | - | (Sønderby et al., 2010) |
|  | **O** 1.1.1.85 | *IMDH1* | 3-isopropylmalate dehydrogenase 1, chloroplastic | Q9FMT1 | 32271 | - | - | (Sønderby et al., 2010) |
|  | **O** 2.3.3.17 | *MAM1* | Methylthioalkylmalate synthase 1, chloroplastic | Q9FG67 | 50624 | - | - | (Sønderby et al., 2010) |
|  | **O** 4.2.1.33 | *IIL1* | 3-isopropylmalate dehydratase | Q94AR8 | 32287 | - | - | (Sønderby et al., 2010) |
|  | **O** 1.14.14.42 | *CYP79F1* | Dihomomethionine N-hydroxylase | Q949U1 | 51972 | - | - | (Sønderby et al., 2010) |
|  | **O** 1.14.14.43 | *CYP83A1* | Cytochrome P450 83A1 | P48421 | 51992 | - | - | (Sønderby et al., 2010) |
|  | **O** 3.4.19.16 | *GGP1* | *Gamma-glutamyl peptidase* | Q9M0A7 | 52756 | - | - | (Geu-Flores et al., 2009) |
|  | **O** 4.4.1.- | *SUR1* | S-alkyl-thiohydroximate lyase | Q9SIV0 | - | - | - | (Sønderby et al., 2010) |
|  | **O** 2.4.1.195 | *UGT74B1* | UDP-glycosyltransferase 74B1 | O48676 | 52148 | - | - | (Sønderby et al., 2010) |
|  | **O** 2.8.2.24 | *SOT16* | Cytosolic sulfotransferase 16 | Q9C9D0 | 52736 | - | - | (Piotrowski et al., 2004) |
|  | **O** 3.2.1.147 | *TGG1* | Myrosinase 1 | P37702 | - | GCF_000001735.4 | NP_851077.1 | (Angelino et al., 2015) |
|  | **O** 3.2.1.86 | *CfPbgS* | 6-phospho-beta-glucosidase | A0A921HU83 | - | - | - | (Watanabe et al., 2021) |
|  | **O** none | *saxA* | Isothiocyanate hydrolase | UPI000505CD2A* | - | GCF_037136885.1 | WP_336826373.1 | (van den Bosch et al., 2018) |
| **Phytic acid** | **O** 5.5.1.4 | *MIPS* | Inositol-3-phosphatesynthase | Q9S7U0 | 10716 | - | - | (Sharma et al., 2020) |
|  | **O** 3.1.3.25 | *IMP1* | Inositol monophosphatase 1 | P54926 | 24056 | - | - | (Styer et al., 2004) |
|  | **O** 2.7.1.64 | *MIK* | myo-inositol 3-kinase | Q93Z01 | 21804 | - | - | (Silva et al., 2021) |
|  | **O** 2.7.1.134 | *ITPK3* | Inositol-tetrakisphosphate 1-kinase 3 | Q9SUG3 | 12452 | - | - | (Silva et al., 2021) |
|  | **O** 2.7.8.11 | *PIS2* | phosphatidyl inositol synthase | Q8GUK6 | 11580 | - | - | (Silva et al., 2021) |
|  | **O** 2.7.1.67 | *PI4KB1* | Phosphatidylinositol 4-kinase, | Q9FMJ0 | 19877 | - | - | (Silva et al., 2021) |
|  | **O** 2.7.1.68 | *PIP5K4* | Phosphatidylinositol 4-phosphate 5-kinase | Q9M1K2 | 14425 | - | - | (Silva et al., 2021) |
|  | **O** 3.1.4.11 | *PLC1* | phospholipase C | Q39032 | 33179 | - | - | (Silva et al., 2021) |
|  | **O** 2.7.1.158 | *IPK1* | Inositol-pentakisphosphate 2-kinase | B8AVX5 | 20313 | - | - | (Silva et al., 2021) |
|  | **O** 3.1.3.8 | *phyA* | phytase A | Q9C1T1 | 16989 | GCF_000002855.4 | *XP_001821210.1* | (van Hartingsveldt et al., 1993) |
|  | **O** 3.1.3.72 | *phyA* | 5-phytase | Q7WUJ1 | - | - | - | (Greiner et al., 2002) |
|  | **O** 3.1.3.26 | *appA* | 4-phytase | P07102 | 68308 | - | - | (Greiner et al., 2002) |
|  | **O** 3.1.3.62 | *PAP02* | multiple inositol-polyphosphate phosphatase | G7YZH4 | 77115 |  |  | (Greiner et al., 2002) |
| **Oxalate** | **O** 4.1.3.1 | aceA | Isocitrate lyase | P0A9G6 | 13245 | - | - | (Hoyt et al., 1988) |
|  | **O** 1.1.3.15 | GLO2 | Glycolate oxidase | Q9LRS0 | 25311 | - | - | (Sakuraba et al., 2012) |
|  | **O** 2.3.3.9 | MLS | Malate synthase | Q9LZC3 | 18181 | - | - | (Cornah et al., 2004) |
|  | **O** 1.1.1.37 | MDH1 | Malate dehydrogenase 1, cytoplasmic | P93819 | 21432 | - | - | (Huang et al., 2018) |
|  | **O** 3 .7.1.1 | PDP | Petal death protein | Q05957 | 24432 | - | - | (Lu et al., 2005) |
|  | **O** 4.1.1.3 | yisK | Oxaloacetate tautomerase | O06724 | 15641 | - | - | (Guo et al., 2024) |
|  | **O** 1.11.1.11 | APX1 | L-ascorbate peroxidase 1, cytosolic | Q05431 | 22996 | - | - | (Davletova et al., 2005) |
|  | **O** 1.10.3.3 | AAO | L-ascorbate oxidase | Q8LPL3 | 30243 | - | - | (Yamamoto et al., 2005) |
|  | **O** 4.1.1.2 | *oxdC* | Oxalate decarboxylase | O34714 | RHEA:16509 | GCF_000009045.1 | NP_391204.1 | (Tanner et al., 2001) |
|  | **O** 2.8.3.16 | *frc* | Formyl-CoA:oxalate CoA-transferase | P69902 | RHEA:16545 | GCF_000005845.2 | NP_416875.1 | (Toyota et al., 2008) |
|  | **O** 6.2.1.8 | *PCS60* | Oxalate-CoA ligase | P38137 | RHEA:18293 | GCF_000146045.2 | NP_009781.3 | (Foster & Nakata, 2014) |
|  | **O** 4.1.1.8 | *oxc* | Oxalyl-CoA decarboxylase | P0AFI0 | RHEA:19333 | GCF_000005845.2 | NP_416874.1 | (Werther et al., 2010) |
|  | **O** 3.1.2.10 | - | - | - | - | - | - | (Li et al., 2022) |
|  | **O** 1.2.3.4 | oxO | Oxalate oxidase GF-2.8 | P15290 | 21880 | GCF_018294505.1 | NM_001428001.1 | (Dratewka-Kos et al., 1989) |

*UniParc ID

### S2 Methods

**Figure considerations.** OrthoFinder is a comparative genomics tool that enables the inference of orthogroups. It does so by performing an all-vs-all similarity search (DIAMOND), normalizing for sequence-length bias and phylogenetic distance, constructing a protein graph, and then clustering and partitioning the graph into groups using the MCL algorithm (each protein belongs to one orthogroup per run). Orthogroups (OGs) can be understood as a set of genes derived from a single gene in the last common ancestor of all the species being considered. OrthoFinder extends this concept from genes to proteins using proteomes instead of genomes, where its predicted OGs can be used to look at protein presence/absence across different taxa. Specifically, OrthoFinder was designed to be “fast and scalable” across hundreds of genomes and to work across large evolutionary distances, which is why it was chosen to look at fungi and bacteria simultaneously. These OGs are therefore useful to answer the question of whether a given proteome encodes a member of an enzyme's homologous family in a more refined way than a simple blast. However, it is important to note that these OGs are dataset dependent and are specific to the species and proteomes being used (adding/removing species can split/merge groups). Additionally, these groups can include paralogs (so a single OG may mix subfunctions). Therefore, for the purpose of this review, we will be naming these OGs as functional groups. A schematic overview of the steps followed can be found in Figure S1.

**Enzyme and species list*.*** A curated enzyme list was made for the known enzymes involved in the degradation of compounds covered in this review (Table S1). Enzymes were included only if they were annotated on UniProt (Bateman et al., 2025) with evidence at the protein or transcript level or if there was a paper available experimentally validating the function of the enzyme. A species list was made covering relevant Lactic Acid Bacteria (LAB) (Qiao et al., 2022; Rossi, 2023), yeast, and filamentous fungi relevant for food fermentation (Table S2). Species from the Qualified Presumption of Safety (Allende et al., 2025) (QPS) list were also included.

***Species Proteome sequences retrieval.*** The NCBI Datasets software (O’Leary et al., 2024) (v18.1.0) was used to download each species' summary and proteome file. Only species with a RefSeq entry were included. Additionally, bacterial genomes were filtered, using each species' NCBI summary.json metadata file, to only include those with a CheckM (Parks et al., 2015) completeness value greater than 95% and a contamination value lower than 5%. Fungal species were manually checked using BUSCO (Simão et al., 2015; Tegenfeldt et al., 2025) (v6.0.0, mode protein) to ensure a completeness greater than 90% and a duplication level lower than 5%.

***Enzyme sequences.*** The UniProt website API (Bateman et al., 2025)was used to retrieve, for each enzyme entry, its source organism, and its associated genomic NCBI protein accession number. For sequences with no associated genomic NCBI protein accession numbers, the NCBI BLAST+ suite (Camacho et al., 2009) (v2.14.1) was used to identify a genome containing the given enzyme. Only matches with an identity greater than 97 were considered. For each enzyme, both their proteome and genomic NCBI protein accession number needed to be obtained, as this data is required to both run and parse the output of OrthoFinder.

***Inference of functional groups.*** OrthoFinder (Emms & Kelly, 2015) (v3.0.1b1) was used for orthology inference. OrthoFinder is a comparative genomics program that takes species proteomes as input and automatically infers orthogroups, among other outputs. The proteomes of all species and a species phylogenetic tree generated using PhyloT (Ivica Letunić, n.d.; Schoch et al., 2020) were provided to OrthoFinder as input. The following parameters were used: -S diamond -M msa -A mafft -T fasttree. The N0.tsv Orthologous Group output file was filtered to retain only the orthogroups (OGs) containing the NCBI protein accession number of enzymes in our list. Additionally, for the main figure, the OG columns corresponding to both tannases and phytases were merged. For tannases, the OG of the bacterial tanLpl was merged with the OG of the fungal AotanA. For phytases, the OG phyA and Blon_0263 were merged with the OG of appA. The final figure was generated in R using ggplot2 (Hadley Wickham, 2016) (v3.5.2) and ggtree (Yu et al., 2017) (v3.17.1.1).


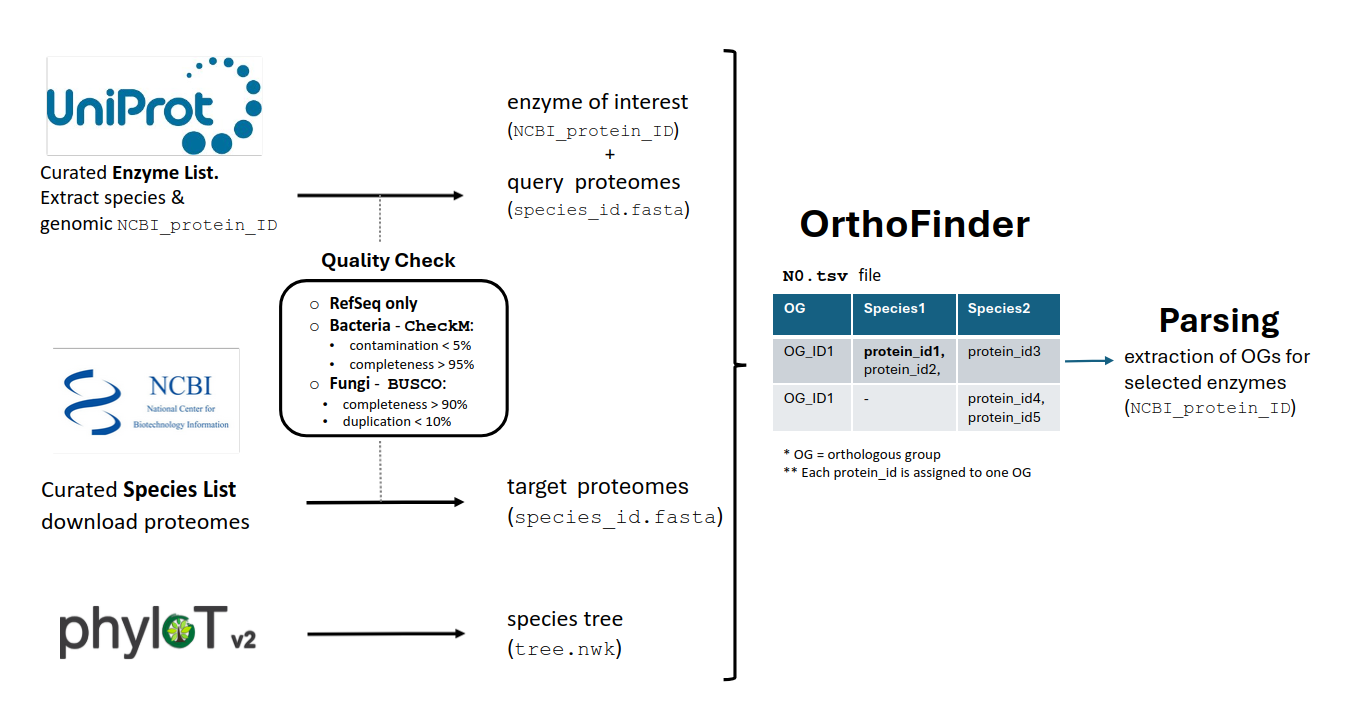


Figure S1: Data extraction and parsing steps are used to generate the main figure. For each target enzyme in UniProt, their source organism and associated NCBI genomic protein accession number were downloaded using the UniProt API. For the selected fermentative species, their proteomes were downloaded from NCBI Genomes. A species tree was generated using phyloT. Orthologous groups were generated using OrthoFinder.

### References

Achterholt, S., Priefert, H., & Steinbüchel, A. (2000). Identification of Amycolatopsis sp. strain HR167 genes, involved in the bioconversion of ferulic acid to vanillin. *Applied Microbiology and Biotechnology*, *54*(6), 799–807. https://doi.org/10.1007/s002530000431

Allende, A., Alvarez‐Ordóñez, A., Bortolaia, V., Bover‐Cid, S., De Cesare, A., Dohmen, W., Guillier, L., Jacxsens, L., Nauta, M., Mughini‐Gras, L., Ottoson, J., Peixe, L., Perez‐Rodriguez, F., Skandamis, P., Suffredini, E., Cocconcelli, P. S., Fernández Escámez, P. S., Maradona, M. P., Querol, A., … Herman, L. (2025). Update of the list of qualified presumption of safety (QPS) recommended microbiological agents intentionally added to food or feed as notified to EFSA 21: Suitability of taxonomic units notified to EFSA until September 2024. *EFSA Journal*, *23*(1). https://doi.org/10.2903/j.efsa.2025.9169

Angelino, D., Dosz, E. B., Sun, J., Hoeflinger, J. L., Van Tassell, M. L., Chen, P., Harnly, J. M., Miller, M. J., & Jeffery, E. H. (2015). Myrosinase-dependent and –independent formation and control of isothiocyanate products of glucosinolate hydrolysis. *Frontiers in Plant Science*, *6*. https://doi.org/10.3389/fpls.2015.00831

Bannenberg, G., Martínez, M., Hamberg, M., & Castresana, C. (2009). Diversity of the Enzymatic Activity in the Lipoxygenase Gene Family of Arabidopsis thaliana. *Lipids*, *44*(2), 85–95. https://doi.org/10.1007/s11745-008-3245-7

Bateman, A., Martin, M.-J., Orchard, S., Magrane, M., Adesina, A., Ahmad, S., Bowler-Barnett, E. H., Bye-A-Jee, H., Carpentier, D., Denny, P., Fan, J., Garmiri, P., Gonzales, L. J. da C., Hussein, A., Ignatchenko, A., Insana, G., Ishtiaq, R., Joshi, V., Jyothi, D., … Zhang, J. (2025). UniProt: the Universal Protein Knowledgebase in 2025. *Nucleic Acids Research*, *53*(D1), D609–D617. https://doi.org/10.1093/nar/gkae1010

Bell-Lelong, D. A., Cusumano, J. C., Meyer, K., & Chapple, C. (1997). Cinnamate-4-Hydroxylase Expression in Arabidopsis (Regulation in Response to Development and the Environment). *Plant Physiology*, *113*(3), 729–738. https://doi.org/10.1104/pp.113.3.729

Bennetzen, J. L., & Hall, B. D. (1982). The primary structure of the Saccharomyces cerevisiae gene for alcohol dehydrogenase. *Journal of Biological Chemistry*, *257*(6), 3018–3025. https://doi.org/10.1016/S0021-9258(19)81067-0

Boden, R., Borodina, E., Wood, A. P., Kelly, D. P., Murrell, J. C., & Schäfer, H. (2011). Purification and Characterization of Dimethylsulfide Monooxygenase from Hyphomicrobium sulfonivorans. *Journal of Bacteriology*, *193*(5), 1250–1258. https://doi.org/10.1128/JB.00977-10

Brunel, F., & Davison, J. (1988). Cloning and sequencing of Pseudomonas genes encoding vanillate demethylase. *Journal of Bacteriology*, *170*(10), 4924–4930. https://doi.org/10.1128/jb.170.10.4924-4930.1988

Camacho, C., Coulouris, G., Avagyan, V., Ma, N., Papadopoulos, J., Bealer, K., & Madden, T. L. (2009). BLAST+: architecture and applications. *BMC Bioinformatics*, *10*(1), 421. https://doi.org/10.1186/1471-2105-10-421

Carrión, O., Curson, A. R. J., Kumaresan, D., Fu, Y., Lang, A. S., Mercadé, E., & Todd, J. D. (2015). A novel pathway producing dimethylsulphide in bacteria is widespread in soil environments. *Nature Communications*, *6*(1), 6579. https://doi.org/10.1038/ncomms7579

Colnaghi, R., Cassinelli, G., Drummond, M., Forlani, F., & Pagani, S. (2001). Properties of the Escherichia coli rhodanese-like protein SseA: Contribution of the active-site residue Ser240 to sulfur donor recognition. *FEBS Letters*, *500*(3), 153–156. https://doi.org/10.1016/S0014-5793(01)02610-2

Cornah, J. E., Germain, V., Ward, J. L., Beale, M. H., & Smith, S. M. (2004). Lipid Utilization, Gluconeogenesis, and Seedling Growth in Arabidopsis Mutants Lacking the Glyoxylate Cycle Enzyme Malate Synthase. *Journal of Biological Chemistry*, *279*(41), 42916–42923. https://doi.org/10.1074/jbc.M407380200

Davies, W. D., & Davidson, B. E. (1982). The nucleotide sequence of aroG, the gene for 3-deoxy-D-arabinobeptulosonate-7-phosphate synthetase (phe) in Escherichia coli K12. *Nucleic Acids Research*, *10*(13), 4045–4058. https://doi.org/10.1093/nar/10.13.4045

Davletova, S., Rizhsky, L., Liang, H., Shengqiang, Z., Oliver, D. J., Coutu, J., Shulaev, V., Schlauch, K., & Mittler, R. (2005). Cytosolic Ascorbate Peroxidase 1 Is a Central Component of the Reactive Oxygen Gene Network of Arabidopsis. *The Plant Cell*, *17*(1), 268–281. https://doi.org/10.1105/tpc.104.026971

De Bont, J. A. M., Van Dijken, J. P., & Harder, W. (1981). Dimethyl Sulphoxide and Dimethyl Sulphide as a Carbon, Sulphur and Energy Source for Growth of Hyphomicrobium S. *Microbiology*, *127*(2), 315–323. https://doi.org/10.1099/00221287-127-2-315

Dobric, N., Limsowtin, G. K. Y., Hillier, A. J., Dudman, N. P. B., & Davidson, B. E. (2000). Identification and characterization of a cystathionine beta/gamma-lyase from Lactococcus lactis ssp. cremoris MG1363. *FEMS Microbiology Letters*, *182*(2), 249–254. https://doi.org/10.1111/j.1574-6968.2000.tb08903.x

Dratewka-Kos, E., Rahman, S., Grzelczak, Z. F., Kennedy, T. D., Murray, R. K., & Lane, B. G. (1989). Polypeptide structure of germin as deduced from cDNA sequencing. *The Journal of Biological Chemistry*, *264*(9), 4896–4900. http://www.ncbi.nlm.nih.gov/pubmed/2925674

Duan, H., Huang, M.-Y., Palacio, K., & Schuler, M. A. (2005). Variations in CYP74B2 (Hydroperoxide Lyase) Gene Expression Differentially Affect Hexenal Signaling in the Columbia and Landsberg erecta Ecotypes of Arabidopsis. *Plant Physiology*, *139*(3), 1529–1544. https://doi.org/10.1104/pp.105.067249

Duncan, K., Chaudhuri, S., Campbell, M. S., & Coggins, J. R. (1986). The overexpression and complete amino acid sequence of Escherichia coli 3-dehydroquinase. *Biochemical Journal*, *238*(2), 475–483. https://doi.org/10.1042/bj2380475

Dunlevy, J. D., Soole, K. L., Perkins, M. V., Dennis, E. G., Keyzers, R. A., Kalua, C. M., & Boss, P. K. (2010). Two O-methyltransferases involved in the biosynthesis of methoxypyrazines: grape-derived aroma compounds important to wine flavour. *Plant Molecular Biology*, *74*(1–2), 77–89. https://doi.org/10.1007/s11103-010-9655-y

Emms, D. M., & Kelly, S. (2015). OrthoFinder: solving fundamental biases in whole genome comparisons dramatically improves orthogroup inference accuracy. *Genome Biology*, *16*(1), 157. https://doi.org/10.1186/s13059-015-0721-2

Entsch, B., Nan, Y., Weaich, K., & Scott, K. F. (1988). Sequence and organization of pobA, the gene coding for p-hydroxybenzoate hydroxylase, an inducible enzyme from Pseudomonas aeruginosa. *Gene*, *71*(2), 279–291. https://doi.org/10.1016/0378-1119(88)90044-3

Eyice, Ö., Myronova, N., Pol, A., Carrión, O., Todd, J. D., Smith, T. J., Gurman, S. J., Cuthbertson, A., Mazard, S., Mennink-Kersten, M. A. S. H., Bugg, T. D. H., Andersson, K. K., Johnston, A. W. B., Op Den Camp, H. J. M., & Schäfer, H. (2018). Bacterial SBP56 identified as a Cu-dependent methanethiol oxidase widely distributed in the biosphere. *ISME Journal*, *12*(1), 145–160. https://doi.org/10.1038/ismej.2017.148

Foster, J., & Nakata, P. A. (2014). An oxalyl‐CoA synthetase is important for oxalate metabolism in Saccharomyces cerevisiae. *FEBS Letters*, *588*(1), 160–166. https://doi.org/10.1016/j.febslet.2013.11.026

Fotheringham, I. G., Dacey, S. A., Taylor, P. P., Smith, T. J., Hunter, M. G., Finlay, M. E., Primrose, S. B., Parker, D. M., & Edwards, R. M. (1986). The cloning and sequence analysis of the aspC and tyrB genes from Escherichia coli K12. Comparison of the primary structures of the aspartate aminotransferase and aromatic aminotransferase of E. coli with those of the pig aspartate aminotransferase isoenzymes. *Biochemical Journal*, *234*(3), 593–604. https://doi.org/10.1042/bj2340593

Geu-Flores, F., Nielsen, M. T., Nafisi, M., Møldrup, M. E., Olsen, C. E., Motawia, M. S., & Halkier, B. A. (2009). Glucosinolate engineering identifies a γ-glutamyl peptidase. *Nature Chemical Biology*, *5*(8), 575–577. https://doi.org/10.1038/nchembio.185

Gowri, G., Bugos, R. C., Campbell, W. H., Maxwell, C. A., & Dixon, R. A. (1991). Stress Responses in Alfalfa ( Medicago sativa L.). *Plant Physiology*, *97*(1), 7–14. https://doi.org/10.1104/pp.97.1.7

Greiner, R., Larsson Alminger, M., Carlsson, N. gunnar, Muzquiz, M., Burbano, C., Cuadrado, C., Pedrosa, M. M., & Goyoaga, C. (2002). Pathway of dephosphorylation of myo-inositol hexakisphosphate by phytases of legume seeds. *Journal of Agricultural and Food Chemistry*, *50*(23), 6865–6870. https://doi.org/10.1021/jf025620t

Guo, T., Sperber, A. M., Krieger, I. V., Duan, Y., Chemelewski, V. R., Sacchettini, J. C., & Herman, J. K. (2024). Bacillus subtilis YisK possesses oxaloacetate decarboxylase activity and exhibits Mbl-dependent localization. *Journal of Bacteriology*, *206*(1). https://doi.org/10.1128/jb.00202-23

Gus-Mayer, S., Brunner, H., Schneider-Poetsch, H. A. W., & Rüdiger, W. (1994). Avenacosidase from oat: purification, sequence analysis and biochemical characterization of a new member of the BGA family of β-glucosidases. *Plant Molecular Biology*, *26*, 909–921. https://doi.org/10.1007/BF00028858

Hadley Wickham. (2016). *ggplot2: Elegant Graphics for Data Analysis*. Springer-Verlag New York. https://ggplot2.tidyverse.org

Hoyt, J. C., Robertson, E. F., Berlyn, K. A., & Reeves, H. C. (1988). Escherichia coli isocitrate lyase: properties and comparisons. *Biochimica et Biophysica Acta (BBA) - General Subjects*, *966*(1), 30–35. https://doi.org/10.1016/0304-4165(88)90125-0

Huang, J., Niazi, A. K., Young, D., Rosado, L. A., Vertommen, D., Bodra, N., Abdelgawwad, M. R., Vignols, F., Wei, B., Wahni, K., Bashandy, T., Bariat, L., Van Breusegem, F., Messens, J., & Reichheld, J. P. (2018). Self-protection of cytosolic malate dehydrogenase against oxidative stress in Arabidopsis. *Journal of Experimental Botany*, *69*(14), 3491–3505. https://doi.org/10.1093/jxb/erx396

Ivica Letunić. (n.d.). *phyloT v2: a phylogenetic tree generator based on NCBI/GTDB taxonomy. Available at: https://phylot.biobyte.de/ (Accessed October 2025)*.

Jez, J. M., Bowman, M. E., Dixon, R. A., & Noel, J. P. (2000). Structure and mechanism of the evolutionarily unique plant enzyme chalcone isomerase. *Nature Structural Biology*, *7*(9), 786–791. https://doi.org/10.1038/79025

Jiménez, N., Curiel, J. A., Reverén, I., de las Rivas, B., & Muñoz, R. (2013). Uncovering the Lactobacillus plantarum WCFS1 Gallate Decarboxylase Involved in Tannin Degradation. *Applied and Environmental Microbiology*, *79*(14), 4253–4263. https://doi.org/10.1128/AEM.00840-13

Kondo, K., Wakabayashi, S., Yagi, T., & Kagamiyama, H. (1984). The complete amino acid sequence of aspartate aminotransferase from Escherichia coli: Sequence comparison with pig isoenzymes. *Biochemical and Biophysical Research Communications*, *122*(1), 62–67. https://doi.org/10.1016/0006-291X(84)90439-X

Kuramitsu, S., Ogawa, T., Ogawa, H., & Kagamiyama, H. (1985). Branched-Chain Amino Acid Aminotransferase of Escherichia coli: Nucleotide Sequence of the ilvE Gene and the Deduced Amino Acid Sequence1. *The Journal of Biochemistry*, *97*(4), 993–999. https://doi.org/10.1093/oxfordjournals.jbchem.a135176

Lee, D., Meyer, K., Chapple, C., & Douglas, C. J. (1997). Antisense suppression of 4-coumarate:coenzyme A ligase activity in Arabidopsis leads to altered lignin subunit composition. *The Plant Cell*, *9*(11), 1985–1998. https://doi.org/10.1105/tpc.9.11.1985

Li, P., Liu, C., Luo, Y., Shi, H., Li, Q., PinChu, C., Li, X., Yang, J., & Fan, W. (2022). Oxalate in Plants: Metabolism, Function, Regulation, and Application. *Journal of Agricultural and Food Chemistry*, *70*(51), 16037–16049. https://doi.org/10.1021/acs.jafc.2c04787

Liou, G., Chiang, Y.-C., Wang, Y., & Weng, J.-K. (2018). Mechanistic basis for the evolution of chalcone synthase catalytic cysteine reactivity in land plants. *Journal of Biological Chemistry*, *293*(48), 18601–18612. https://doi.org/10.1074/jbc.RA118.005695

Lu, Z., Feng, X., Song, L., Han, Y., Kim, A., Herzberg, O., Woodson, W. R., Martin, B. M., Mariano, P. S., & Dunaway-Mariano, D. (2005). Diversity of Function in the Isocitrate Lyase Enzyme Superfamily:  The Dianthus caryophyllus Petal Death Protein Cleaves α-Keto and α-Hydroxycarboxylic Acids. *Biochemistry*, *44*(50), 16365–16376. https://doi.org/10.1021/bi051776l

Millar, G., & Coggins, J. R. (1986). The complete amino acid sequence of 3‐dehydroquinate synthase of Escherichia coli K 12. *FEBS Letters*, *200*(1), 11–17. https://doi.org/10.1016/0014-5793(86)80501-4

Mittasch, J., Böttcher, C., Frolova, N., Bönn, M., & Milkowski, C. (2014). Identification of UGT84A13 as a candidate enzyme for the first committed step of gallotannin biosynthesis in pedunculate oak (Quercus robur). *Phytochemistry*, *99*, 44–51. https://doi.org/10.1016/j.phytochem.2013.11.023

Mukai, N., Masaki, K., Fujii, T., Kawamukai, M., & Iefuji, H. (2010). PAD1 and FDC1 are essential for the decarboxylation of phenylacrylic acids in Saccharomyces cerevisiae. *Journal of Bioscience and Bioengineering*, *109*(6), 564–569. https://doi.org/10.1016/j.jbiosc.2009.11.011

Nogales, J., Canales, Á., Jiménez‐Barbero, J., Serra, B., Pingarrón, J. M., García, J. L., & Díaz, E. (2011). Unravelling the gallic acid degradation pathway in bacteria: the gal cluster from Pseudomonas putida. *Molecular Microbiology*, *79*(2), 359–374. https://doi.org/10.1111/j.1365-2958.2010.07448.x

O’Leary, N. A., Cox, E., Holmes, J. B., Anderson, W. R., Falk, R., Hem, V., Tsuchiya, M. T. N., Schuler, G. D., Zhang, X., Torcivia, J., Ketter, A., Breen, L., Cothran, J., Bajwa, H., Tinne, J., Meric, P. A., Hlavina, W., & Schneider, V. A. (2024). Exploring and retrieving sequence and metadata for species across the tree of life with NCBI Datasets. *Scientific Data*, *11*(1), 732. https://doi.org/10.1038/s41597-024-03571-y

Ono, B., Tanaka, K., Naito, K., Heike, C., Shinoda, S., Yamamoto, S., Ohmori, S., Oshima, T., & Toh-e, A. (1992). Cloning and characterization of the CYS3 (CYI1) gene of Saccharomyces cerevisiae. *Journal of Bacteriology*, *174*(10), 3339–3347. https://doi.org/10.1128/jb.174.10.3339-3347.1992

Parks, D. H., Imelfort, M., Skennerton, C. T., Hugenholtz, P., & Tyson, G. W. (2015). CheckM: assessing the quality of microbial genomes recovered from isolates, single cells, and metagenomes. *Genome Research*, *25*(7), 1043–1055. https://doi.org/10.1101/gr.186072.114

Piotrowski, M., Schemenewitz, A., Lopukhina, A., Müller, A., Janowitz, T., Weiler, E. W., & Oecking, C. (2004). Desulfoglucosinolate Sulfotransferases from Arabidopsis thaliana Catalyze the Final Step in the Biosynthesis of the Glucosinolate Core Structure. *Journal of Biological Chemistry*, *279*(49), 50717–50725. https://doi.org/10.1074/jbc.M407681200

Pulkkinen, M., Zhou, X., Lampi, A.-M., & Piironen, V. (2016). Determination and stability of divicine and isouramil produced by enzymatic hydrolysis of vicine and convicine of faba bean. *Food Chemistry*, *212*, 10–19. https://doi.org/10.1016/j.foodchem.2016.05.077

Qiao, N., Wittouck, S., Mattarelli, P., Zheng, J., Lebeer, S., Felis, G. E., & Gänzle, M. G. (2022). After the storm—Perspectives on the taxonomy of Lactobacillaceae. *JDS Communications*, *3*(3), 222–227. https://doi.org/10.3168/jdsc.2021-0183

Ren, B., Wu, M., Wang, Q., Peng, X., Wen, H., McKinstry, W. J., & Chen, Q. (2013). Crystal Structure of Tannase from Lactobacillus plantarum. *Journal of Molecular Biology*, *425*(15), 2737–2751. https://doi.org/10.1016/j.jmb.2013.04.032

Rossi, F. (2023). Special Issue “Functional Characterization of Lactic Acid Bacteria”: Editorial. *Microorganisms*, *11*(5), 1190. https://doi.org/10.3390/microorganisms11051190

Sakuraba, Y., Schelbert, S., Park, S.-Y., Han, S.-H., Lee, B.-D., Andrès, C. B., Kessler, F., Hörtensteiner, S., & Paek, N.-C. (2012). Chlorophyll Catabolic Enzymes Interact at Light-Harvesting Complex II for Chlorophyll Detoxification during Leaf Senescence in Arabidopsis. *The Plant Cell*, *24*(2), 507–518. https://doi.org/10.1105/tpc.111.089474

Schoch, C. L., Ciufo, S., Domrachev, M., Hotton, C. L., Kannan, S., Khovanskaya, R., Leipe, D., Mcveigh, R., O’Neill, K., Robbertse, B., Sharma, S., Soussov, V., Sullivan, J. P., Sun, L., Turner, S., & Karsch-Mizrachi, I. (2020). NCBI Taxonomy: a comprehensive update on curation, resources and tools. *Database*, *2020*. https://doi.org/10.1093/database/baaa062

Sharma, N., Chaudhary, C., & Khurana, P. (2020). Wheat Myo-inositol phosphate synthase influences plant growth and stress responses via ethylene mediated signaling. *Scientific Reports*, *10*(1), 10766. https://doi.org/10.1038/s41598-020-67627-w

Shibuya, M., Hoshino, M., Katsube, Y., Hayashi, H., Kushiro, T., & Ebizuka, Y. (2006). Identification of β‐amyrin and sophoradiol 24‐hydroxylase by expressed sequence tag mining and functional expression assay. *The FEBS Journal*, *273*(5), 948–959. https://doi.org/10.1111/j.1742-4658.2006.05120.x

Shibuya, M., Katsube, Y., Otsuka, M., Zhang, H., Tansakul, P., Xiang, T., & Ebizuka, Y. (2009). Identification of a product specific β-amyrin synthase from Arabidopsis thaliana. *Plant Physiology and Biochemistry*, *47*(1), 26–30. https://doi.org/10.1016/j.plaphy.2008.09.007

Silva, V. M., Putti, F. F., White, P. J., & Reis, A. R. dos. (2021). Phytic acid accumulation in plants: Biosynthesis pathway regulation and role in human diet. *Plant Physiology and Biochemistry*, *164*, 132–146. https://doi.org/10.1016/j.plaphy.2021.04.035

Simão, F. A., Waterhouse, R. M., Ioannidis, P., Kriventseva, E. V., & Zdobnov, E. M. (2015). BUSCO: assessing genome assembly and annotation completeness with single-copy orthologs. *Bioinformatics*, *31*(19), 3210–3212. https://doi.org/10.1093/bioinformatics/btv351

Sønderby, I. E., Geu-Flores, F., & Halkier, B. A. (2010). Biosynthesis of glucosinolates – gene discovery and beyond. *Trends in Plant Science*, *15*(5), 283–290. https://doi.org/10.1016/j.tplants.2010.02.005

Styer, J. C., Keddie, J., Spence, J., & Gillaspy, G. E. (2004). Genomic organization and regulation of the LeIMP-1 and LeIMP-2 genes encoding myo-inositol monophosphatase in tomato. *Gene*, *326*(1–2), 35–41. https://doi.org/10.1016/j.gene.2003.09.048

Takahashi, Y., Li, X.-H., Tsukamoto, C., & Wang, K.-J. (2017). Categories and components of soyasaponin in the Chinese wild soybean (Glycine soja) genetic resource collection. *Genetic Resources and Crop Evolution*, *64*(8), 2161–2171. https://doi.org/10.1007/s10722-017-0506-4

Tanner, A., Bowater, L., Fairhurst, S. A., & Bornemann, S. (2001). Oxalate Decarboxylase Requires Manganese and Dioxygen for Activity. *Journal of Biological Chemistry*, *276*(47), 43627–43634. https://doi.org/10.1074/jbc.M107202200

Tchong, S. I., Xu, H., & White, R. H. (2005). L-cysteine desulfidase: An [4Fe-4S] enzyme isolated from Methanocaldococcus jannaschii that catalyzes the breakdown of L-cysteine into pyruvate, ammonia, and sulfide. *Biochemistry*, *44*(5), 1659–1670. https://doi.org/10.1021/bi0484769

Tegenfeldt, F., Kuznetsov, D., Manni, M., Berkeley, M., Zdobnov, E. M., & Kriventseva, E. V. (2025). OrthoDB and BUSCO update: annotation of orthologs with wider sampling of genomes. *Nucleic Acids Research*, *53*(D1), D516–D522. https://doi.org/10.1093/nar/gkae987

Toyota, C. G., Berthold, C. L., Gruez, A., Jónsson, S., Lindqvist, Y., Cambillau, C., & Richards, N. G. J. (2008). Differential Substrate Specificity and Kinetic Behavior of Escherichia coli YfdW and Oxalobacter formigenes Formyl Coenzyme A Transferase. *Journal of Bacteriology*, *190*(7), 2556–2564. https://doi.org/10.1128/JB.01823-07

Ubuka, T., Ohta, J., Akagi, R., Hosaki, Y., Ishimoto, Y., Kiguchi, S., Ikeda, T., & Ishino, K. (1992). Metabolism ofl-cysteine via transamination pathway (3-mercaptopyruvate pathway). *Amino Acids*, *3*(3), 243–252. https://doi.org/10.1007/BF00805999

van den Bosch, T. J. M., Tan, K., Joachimiak, A., & Welte, C. U. (2018). Functional Profiling and Crystal Structures of Isothiocyanate Hydrolases Found in Gut-Associated and Plant-Pathogenic Bacteria. *Applied and Environmental Microbiology*, *84*(14). https://doi.org/10.1128/AEM.00478-18

van Hartingsveldt, W., van Zeijl, C. M. J., Harteveld, G. M., Gouka, R. J., Suykerbuyk, M. E. G., Luiten, R. G. M., van Paridon, P. A., Selten, G. C. M., Veenstra, A. E., van Gorcom, R. F. M., & van den Hondel, C. A. M. J. J. (1993). Cloning, characterization and overexpression of the phytase-encoding gene (phyA) of Aspergillus niger. *Gene*, *127*(1), 87–94. https://doi.org/10.1016/0378-1119(93)90620-I

Wang, L., Gamez, A., Archer, H., Abola, E. E., Sarkissian, C. N., Fitzpatrick, P., Wendt, D., Zhang, Y., Vellard, M., Bliesath, J., Bell, S. M., Lemontt, J. F., Scriver, C. R., & Stevens, R. C. (2008). Structural and Biochemical Characterization of the Therapeutic Anabaena variabilis Phenylalanine Ammonia Lyase. *Journal of Molecular Biology*, *380*(4), 623–635. https://doi.org/10.1016/j.jmb.2008.05.025

Wang, W., Du, G., Yang, G., Zhang, K., Chen, B., & Xiao, G. (2022). A multifunctional enzyme portfolio for α-chaconine and α-solanine degradation in the Phthorimaea operculella gut bacterium Glutamicibacter halophytocola S2 encoded in a trisaccharide utilization locus. *Frontiers in Microbiology*, *13*. https://doi.org/10.3389/fmicb.2022.1023698

Wang, X., Mann, C. J., Bai, Y., Ni, L., & Weiner, H. (1998). Molecular Cloning, Characterization, and Potential Roles of Cytosolic and Mitochondrial Aldehyde Dehydrogenases in Ethanol Metabolism in Saccharomyces cerevisiae. *Journal of Bacteriology*, *180*(4), 822–830. https://doi.org/10.1128/JB.180.4.822-830.1998

Watanabe, H., Usami, R., Kishino, S., Osada, K., Aoki, Y., Morisaka, H., Takahashi, M., Izumi, Y., Bamba, T., Aoki, W., Suganuma, H., & Ogawa, J. (2021). Enzyme systems involved in glucosinolate metabolism in Companilactobacillus farciminis KB1089. *Scientific Reports*, *11*(1), 23715. https://doi.org/10.1038/s41598-021-03064-7

Werther, T., Zimmer, A., Wille, G., Golbik, R., Weiss, M. S., & König, S. (2010). New insights into structure–function relationships of oxalyl CoA decarboxylase from Escherichia coli. *The FEBS Journal*, *277*(12), 2628–2640. https://doi.org/10.1111/j.1742-4658.2010.07673.x

Yamamoto, A., Bhuiyan, Md. N. H., Waditee, R., Tanaka, Y., Esaka, M., Oba, K., Jagendorf, A. T., & Takabe, T. (2005). Suppressed expression of the apoplastic ascorbate oxidase gene increases salt tolerance in tobacco and Arabidopsis plants. *Journal of Experimental Botany*, *56*(417), 1785–1796. https://doi.org/10.1093/jxb/eri167

Yu, G., Smith, D. K., Zhu, H., Guan, Y., & Lam, T. T. (2017). ggtree: an R package for visualization and annotation of phylogenetic trees with their covariates and other associated data. *Methods in Ecology and Evolution*, *8*(1), 28–36. https://doi.org/10.1111/2041-210X.12628
